# Deciphering Methylation Effects on S_2_(ππ*) Internal Conversion in the Simplest Linear α,β-Unsaturated Carbonyl

**DOI:** 10.1021/acs.jpca.3c02582

**Published:** 2023-06-18

**Authors:** Pratip Chakraborty, Rafael C. Couto, Nanna H. List

**Affiliations:** Department of Chemistry, KTH Royal Institute of Technology, SE-10044 Stockholm, Sweden

## Abstract

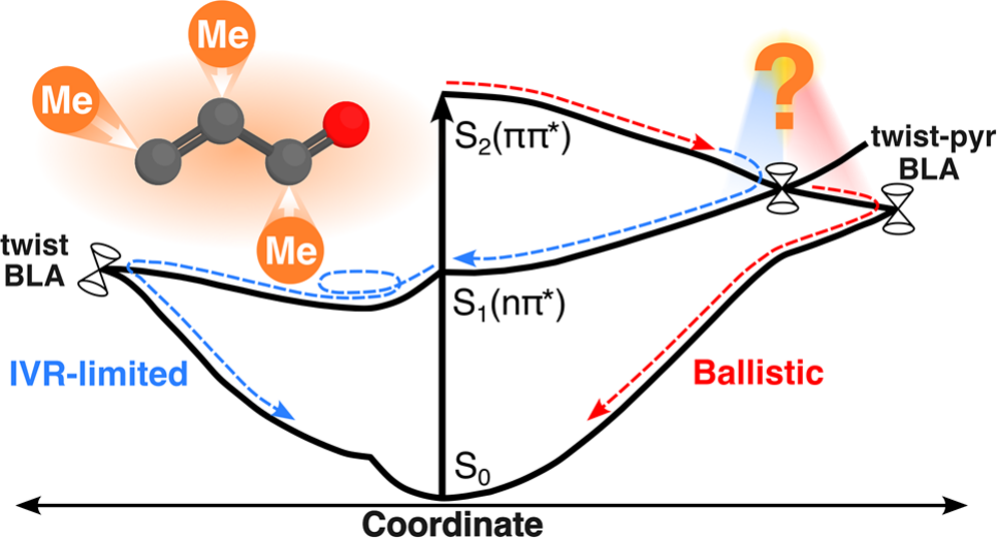

Chemical substituents can influence photodynamics by altering the location of critical points and the topography of the potential energy surfaces (electronic effect) and by selectively modifying the inertia of specific nuclear modes (inertial effects). Using nonadiabatic dynamics simulations, we investigate the impact of methylation on S_2_(ππ*) internal conversion in acrolein, the simplest linear α,β-unsaturated carbonyl. Consistent with time constants reported in a previous time-resolved photoelectron spectroscopy study, S_2_ → S_1_ deactivation occurs on an ultrafast time scale (∼50 fs). However, our simulations do not corroborate the sequential decay model used to fit the experiment. Instead, upon reaching the S_1_ state, the wavepacket bifurcates: a portion undergoes ballistic S_1_ → S_0_ deactivation (∼90 fs) mediated by fast bond-length alternation motion, while the remaining decays on the picosecond time scale. Our analysis reveals that methyl substitution, generally assumed to mainly exert inertial influence, is also manifested in important electronic effects due to its weak electron-donating ability. While methylation at the β C atom gives rise to effects principally of an inertial nature, such as retarding the twisting motion of the terminal −CHCH_3_ group and increasing its coupling with pyramidalization, methylation at the α or carbonyl C atom modifies the potential energy surfaces in a way that also contributes to altering the late S_1_-decay behavior. Specifically, our results suggest that the observed slowing of the picosecond component upon α-methylation is a consequence of a tighter surface and reduced amplitude along the central pyramidalization, effectively restricting the access to the S_1_/S_0_-intersection seam. Our work offers new insight into the S_2_(ππ*) internal conversion mechanisms in acrolein and its methylated derivatives and highlights site-selective methylation as a tuning knob to manipulate photochemical reactions.

## 1 Introduction

Photoexcited dynamics of polyatomic molecules are of significant interest in a multitude of natural processes, such as vision,^1,2^ light harvesting,^3−6^ and the photostability of DNA/RNA nucleobases.^7−9^ In the vicinity of conical intersections, the nonadiabatic coupling becomes significant, driving transitions between electronic states.^10−12^ Chemical substituents can drastically affect such nonadiabatic dynamics.^13−22^ Schuurman and Stolow have separated substituent effects into electronic and inertial components.^23^ The former accounts for the imprints on the electronic structure, i.e., changes in the location of critical points and the topography of the potential energy surfaces (PESs), whereas the latter refers to changes in the inertia of specific nuclear modes that will affect the direction and velocity of the wavepacket on the PESs. The intricate details of how these effects play their part in the nonadiabatic dynamics are not obvious.

Schuurman, Stolow, and co-workers have adopted a systematic approach combining time-resolved photoelectron spectroscopy (TRPES) with electronic-structure calculations (and recently, *ab initio* nonadiabatic dynamics simulations) to study substituent effects on excited-state dynamics, focusing on unsaturated hydrocarbons.^14−19,21−23^ Introducing a methyl group at different positions of unsaturated hydrocarbons is expected to cause specific inertial effects. In allenes, increasing methylation at the terminal C atom gradually slows the twisting motion. This leads to a faster and unconstrained bending motion of the main allene moiety, modifying the pathway of excited-state deactivation.^15^ In cyclopentadienes, an increase in the inertia at the C5 position slows down the dynamics on S_1_ by inhibiting the out-of-plane vibration at that center.^14^

**Figure 1:**
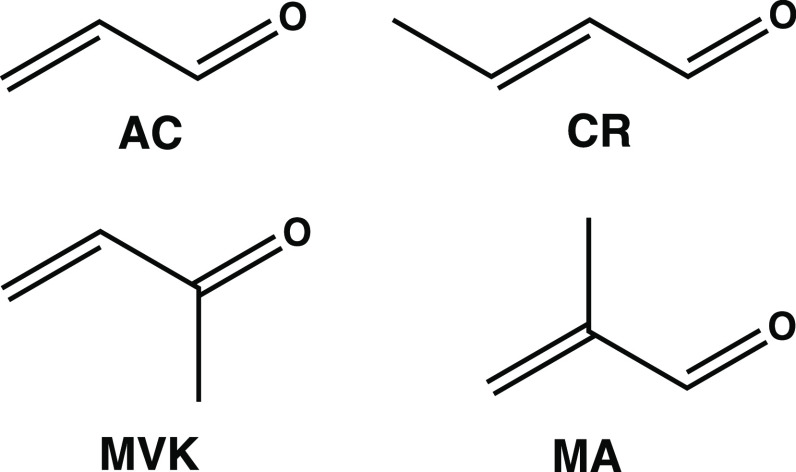
Molecular structures of *s*-trans acrolein (AC), crotonaldehyde (CR), methylvinylketone (MVK), and methacrolein (MA).

Another interesting class of unsaturated hydrocarbons is α,β-unsaturated carbonyls. Acrolein (AC) is the simplest example with its *s*-trans form (see Figure 1), being the most stable conformer at room temperature.^24−28^ Its multifunctional nature has made it a prototype molecule for studying photoexcitation involving both nπ* and ππ* excited states.^25,26,29−42^ Methylation at the α,β and carbonyl C atoms of AC can be expected to slow important nuclear motions associated with internal conversion dynamics. Lee et al. combined femtosecond TRPES and static electronic-structure calculations to investigate methylation effects on the S_2_(ππ*) photodynamics of AC, i.e., considering the methylated derivatives crotanaldehyde (CR), methylvinylketone (MVK), and methacrolein (MA) (see Figure 1).^22^ Their study demonstrated distinct methylation effects on the experimental time scale interpreted to be associated with S_1_ decay. This time scale was found to be significantly faster for CR but almost a factor of-two slower for MA. Static electronic structure calculations and first-order branching space analyses were not enough to explain the origin of these differences. In the absence of dynamical simulations, it was conjectured that specific inertial effects play a major role in accelerating and decelerating dynamics near intersection seams. In CR, it was speculated that the wavepacket might spend more time near the S_1_/S_0_-intersection seam because of a retarded torsional motion of the terminal −CHCH_3_ group, resulting in faster S_1_ decay. On the other hand, in MA, central methylation was proposed to retard the motion along the nonadiabatic coupling vector dominated by pyramidalization at the α C atom, leading to a less efficient and hence slower decay. Recently, the faster TRPES behavior of CR was reinterpreted in a multimode picture.^43^ Specifically, the efficient nonadiabatic transfer in CR was rationalized on the basis of a strong coupling (due to lack of symmetry at the conical intersection) between the torsion and β C atom pyramidalization modes at the intersection. However, these theory-based hypotheses have, to the best of our knowledge, never been examined from a dynamical perspective.

Furthermore, a recent study on the photexcited dynamics of methylated butadienes highlighted the importance of electronic effects of the weakly electron-donating methyl group, influencing nonradiative decay time scales and branching ratios.^19^ Electronic effects of the methylation have also been found to accelerate the photoisomerization of a methylated derivative of all-trans retinal (methylated at the C10 position) in solution.^44−47^ In light of such developments, we aim to resolve the mechanistic details of S_2_(ππ*) internal conversion in AC and the role of inertial and electronic effects in its methylated derivatives using nonadiabatic dynamics simulations.

AC and its methylated derivatives are all present in the atmosphere as volatile organic compounds, being both the precursors and intermediates in several chemical processes.^48−52^ Despite their small size, characterizing the electronic structure of their excited states has proven challenging. An early experimental study on AC from Walsh assigned absorption bands at 412, 387, and 193.5 nm to S_0_ → T_1_(nπ*), S_0_ → S_1_(nπ*), and S_0_ → S_2_(ππ*) transitions, respectively.^29^ In addition, Birge and Leermakers assigned S_0_ → S_1_(nπ*) and S_0_ → T_1_(nπ*) transitions using vibrational electronic spectroscopy along with molecular orbital and spin–orbit coupling calculations for the methylated analogs of AC.^53^ Substantial attention has been devoted to S_1_(nπ*) photodynamics of AC, accessible through UV-A radiation, with a focus on relaxation, isomerization, and dissociation pathways.^30,32,34−37,54^ As such, the S_1_, T_1_, and T_2_ states of AC have been characterized both experimentally and theoretically, and they agree well.^36,37^ On the other hand, the character and vertical excitation energy of the S_2_(ππ*) state of AC have been a contentious topic. Moreover, there has, to our knowledge, been no experimental characterization of the S_0_ → S_2_ transition in CR, MVK, and MA. Several theoretical studies^38−40^ have found S_0_ → S_2_ excitation energies of AC that overestimate the experimental values^29^ by ∼1 eV. This has been attributed to the double-excited character and unresolved mixing between valence and Rydberg states in the Franck–Condon (FC) region.^41,55^ Using the multistate complete active space second-order perturbation theory (MS-CASPT2)^56^ and specially designed active spaces and basis sets, Aquilante et al. were able to characterize the S_2_ state of AC as a valence ππ* excitation.^41^ They also showed that S_1_ through S_3_(nπ*) are the only valence excited states below the Rydberg states. Since then, several theoretical studies have characterized the S_2_ state of AC at the FC point employing high-level theories.^55,57−59^

Experimentally, photoexcitation (∼193 nm) to the S_2_ state of AC has predominantly been pursued to investigate photodissociation and photofragment formation.^42,60−65^ However, the photochemical details of the internal conversion dynamics following S_2_ photoexcitation and the characterization of the conical intersections for nonradiative relaxation have received much less attention. Recently, nonadiabatic *ab initio* multiple spawning (AIMS) simulations^66^ (employing hole–hole Tamm–Dancoff-approximated (*hh*-TDA) density functional theory^67−70^) have been performed for AC initiated from the S_2_ excited state for the prediction of the time-resolved near-edge X-ray absorption fine structure spectrum.^71^ However, the underlying mechanistic details have not been discussed.

In this work, we aim to decipher the mechanistic details of the S_2_(ππ*) internal conversion dynamics in AC and investigate the inertial and electronic effects of methylation. We tackle this by performing and analyzing *hh*-TDA AIMS dynamics simulations across AC and its methylated derivatives. Our resulting dynamical picture provides new insights into the mechanisms underlying the time scales associated with the previous TRPES measurements.^22^

## 2 Computational Methods

To investigate the effects of methylation in the α,β-unsaturated carbonyls, we employed the *hh*-TDA method which provides an effective means of including both static and dynamical electron correlation. In this method, the starting point is an (N + 2) electron reference state, and the ground- and excited-state wave functions are constructed by applying a pair of annihilation operators, which allows for a proper description of excited electronic states characterized by transitions into the lowest unoccupied molecular orbital. Unlike time-dependent density functional theory,^72^ this method can also describe the ground and excited states on an equal footing, allowing for the correct description of the conical intersections between them. All *hh*-TDA calculations employed the 6-31G(d,p) basis set^73−75^ and the ωPBEh^76^ exchange-correlational functional, with a range separation parameter of 0.3 Bohr^–1^ and short-range Hartree–Fock exchange scaled by 0.3 au. In a previous study, these parameters were found to produce relative energetics of the S_0_–S_2_ states that compare favorably with higher-level electronic-structure methods.^71^ Here, we extend this benchmark to consider both relative energies and geometries at selected critical points by comparing to extended multistate complete active space second-order perturbation theory (XMS-CASPT2).^56,77,78^ Further details on the reference calculations and benchmark results are provided in section S1 of the Supporting Information (SI). In short, we find overall good agreement between *hh*-TDA and XMS-CASPT2.

Initial conditions (ICs) (i.e., coordinates and velocities) for computing absorption spectra and AIMS simulations were generated by sampling the ground-state harmonic Wigner distribution at 300 K. A total of 5000 ICs were randomly sampled for each molecule, and vertical transition energies and oscillator strengths were calculated. The resulting stick spectra were convolved with a Gaussian line shape (FWHM = 0.2 eV) and uniformly red-shifted to match the first experimental absorption maximum^22,79^ (Figure S8) for each system. To approximately mimic a typical pump pulse width, ICs for the AIMS simulations were selected from a narrow window of 0.05 eV around a 200 nm (6.20 eV) wavelength. The pump wavelength matches the (pump: 200 nm, probe: 267 nm) TRPES measurements by Lee et al.^22^ For each molecule, we randomly selected 50 (60 for CR) ICs from which to start AIMS dynamics within the independent-first-generation approximation (section S2 for IC convergence analysis).^80^ Note that ten additional ICs were sampled for CR to ensure that the observed stalling of population decay around 600–900 fs (see below) was not due to undersampling. The simulations were initiated on S_2_ and propagated for 2 ps (4 ps for MA to capture its slower ground-state recovery; see below). The equations of motion were integrated using an adaptive time step of 20 au (∼0.48 fs), which was reduced upon encountering nonadiabatic coupling regions. A coupling threshold of 0.005*E*_*h*_/ℏ (scalar product between derivative coupling and nuclear velocity vectors at a given time step) was employed for a trajectory basis function (TBF) to enter and exit a spawning region. TBFs were removed from the simulations when their population fell below 0.01. Furthermore, TBFs in the ground state that did not couple with other TBFs for at least 5 fs were decoupled and terminated. Afterward, the population of such TBFs was considered to contribute to the S_0_ population. Bootstrapping with 1500 samples was used to provide error bars (standard deviations) for the simulated decay time constants. All *hh*-TDA simulations were performed using the TeraChem package.^81−84^ Geometry optimizations and minimum-energy conical intersection (MECI) searches were performed using the DL-FIND library^85^ interfaced with TeraChem. The XMS-CASPT2 calculations were performed using BAGEL.^86,87^

## 3 Results and Discussion

Before discussing the dynamic results, we briefly consider the key critical points characterizing the PESs of the four α,β-unsaturated carbonyls. A more detailed discussion is provided in section S5. The dominant coordinates differentiating these points are the terminal torsion (−CH_2_ or −CHCH_3_ in CR), bond-length alternation (BLA), pyramidalization of the terminal (β) or central (α) C atom (PyrT or PyrC, respectively), and changes in the central backbone angle (labeled by C, N, and W to indicate contracted, neutral, and wide angles, respectively). Definitions of geometric parameters are given in Figure S3 and section S4.

**Figure 2:**
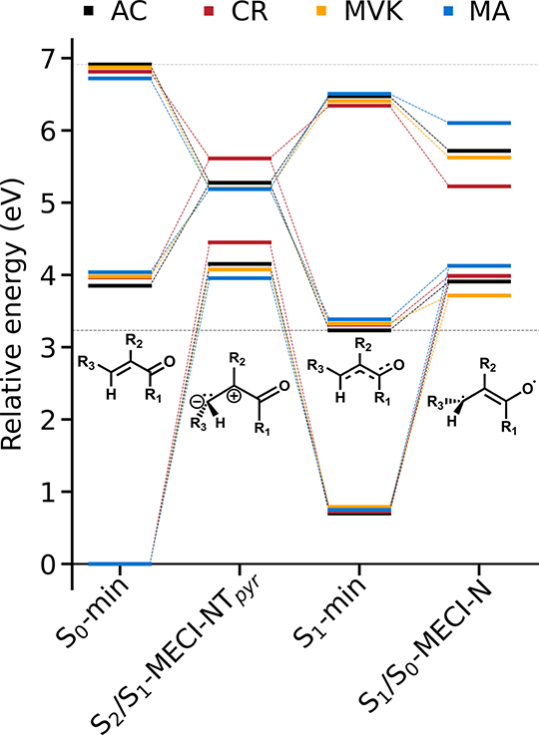
Relative potential energies of important critical points for the four systems relative to their respective S_0_ minimum. Insets indicate the dominant valence structures (AC: R_1,2,3_=H; CR: R_1,2_=H, R_3_=CH_3_; MVK: R_2,3_=H, R_1_=CH_3_; and MA: R_1,3_=H, R_2_=CH_3_). Although energies at the S_0_-min and S_1_-min are similar across all of the molecules, the terminal twist-pyramidalized MECIs (NT_*pyr*_) are destabilized in CR, while S_1_/S_0_-MECI-N is stabilized by the formyl methylation in MVK.

Figure 2 summarizes the energetics of selected critical points obtained at the *hh*-TDA level. At the FC point, all systems exhibit similar planar configurations (ignoring methyl H atoms) and relative energies for the three lowest singlet states. Methylation induces a 0.1–0.2 eV red shift of the S_2_ energies (most pronounced for MA), consistent with, albeit less pronounced than, the trend in the experimental absorption maxima (MA < CR < MVK < AC).^22,79^ The S_2_/S_1_-intersection seam is energetically accessible from the FC point: the lower-energy region of the seam is ∼2 eV below and predominantly features torsion and PyrC (CC_*pyr*_ and/or WC_*pyr*_ for contracted and wide angles, respectively) or torsion with limited pyramidalization (N) in CR (section S5). In addition, we find a higher-energy terminal twist-pyramidalized MECI (NT_*pyr*_). Analogous to the sudden polarization observed for twist-pyramidalized configurations in ethylene and butadiene,^88−91^ the S_2_/S_1_ intersections feature charge-transfer character across the ethylenic unit, with the direction of polarization governed by the dominating pyramidalization center. Due to its weakly electron-donating character through hyperconjugation, we expect methylation at different sites to preferentially stabilize/destabilize certain configurations. In particular, terminal methylation in CR destabilizes S_2_/S_1_-MECI-NT_*pyr*_ by ∼0.4 eV relative to that of the other systems. The S_1_ minimum (S_1_-min) is also planar but has a largely inverted BLA with respect to the FC point. The S_1_/S_0_-intersection seam is located energetically above the minimum, and reaching the lowest-energy S_1_/S_0_-MECI-N requires torsion and further BLA expansion. This MECI exhibits biradicaloid character^92^ with an unpaired electron residing on each of the orthogonal *n*_O_ and terminal C atom 2*p* orbitals. Methylation at the carbonyl C atom in MVK (i.e., methylation of the formyl group) stabilizes this region of the seam, as the weakly electron-donating methyl group effectively compensates for the partial positive charge on the carbonyl C atom. This reduces the energy gap between the S_1_-min and S_1_/S_0_-MECI-N from ∼0.7 (AC/CR/MA) to ∼0.4 eV (MVK). This electronic effect of methylation could affect the ground-state recovery through internal conversion from S_1_. However, our focus on S_2_ photoexcitation implies that the wavepacket is vibrationally hot upon reaching S_1_-min (gaining >3 eV of additional kinetic energy), which could render the lower-energy regions of the seam less important. These aspects will be discussed further below.

**Figure 3:**
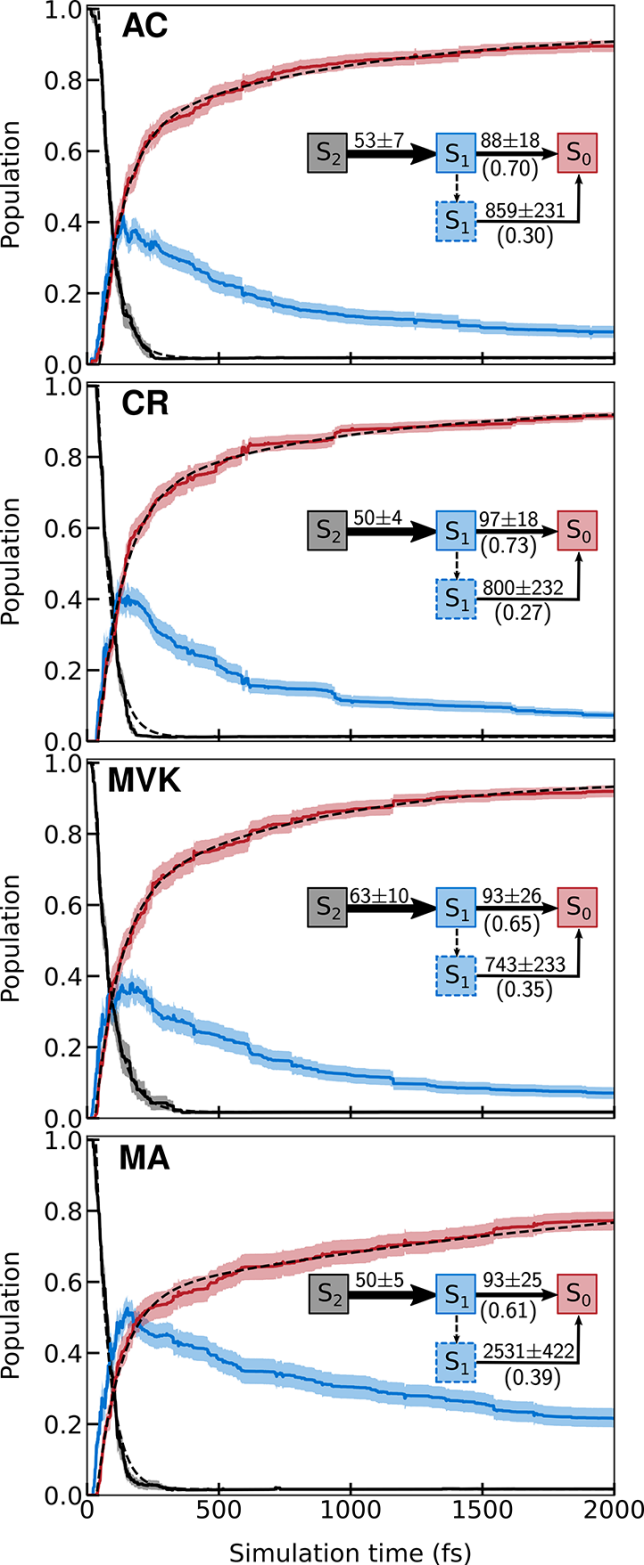
Population dynamics following excitation to the S_2_(ππ*) state, as obtained from the *hh*-TDA-ωPBEh/6-31G(d,p) AIMS simulations. Shaded regions mark one bootstrap standard deviation obtained from 1500 samples. Black dashed lines show fits (section S6). The insets summarize the associated time constants (fs) and amplitudes obtained from the fits to the S_2_ and S_0_ profiles. The dashed arrow and dashed-outlined S_1_ box indicate the portion of the wavepacket that escapes ballistic decay and proceeds to a more statistical, yet IVR-limited regime (see text). The larger S_1_ buildup in MA results from a combination of three factors: (i) its fast S_2_ → S_1_ decay; (ii) the slightly delayed onset of its S_1_ → S_0_ decay; and (iii) a comparatively smaller fraction of the S_1_ wavepacket that undergoes decay in the ballistic regime.

Figure 3 presents the adiabatic population decay profiles obtained from the *hh*-TDA AIMS simulations. Following a short lag time (∼25 fs), the population on S_2_ decays on an ultrafast time scale to S_1_, whose growth is accompanied by a comparably fast ground-state recovery. The maximum in the S_1_ population (0.4–0.5) is reached at ∼200 fs, after which it decreases slowly. The fast-growth/slow-decay S_1_ profile (or two distinct components of S_0_ growth) combined with an intermediate max-population is not reminiscent of a sequential kinetic model (X → A → I → P) that was used in the TRPES study (pump: 200 nm, probe: 267 nm) by Lee et al.^22^ Rather, it signifies that the ground-state recovery occurs on two different time scales. We fitted the S_2_ and S_0_ population profiles to delayed mono- and biexponential decays, respectively (see insets in Figure 3 and section S6). For the S_2_ profiles, this yields time constants of ∼50 fs for AC/CR/MA and ∼60 fs, slightly longer, for MVK. Fitting the S_0_ repopulation yields an ultrafast time constant (∼90 fs) as well as a longer picosecond component. In CR, the ultrafast component dominates with ∼70% amplitude, whereas it drops to ∼60% in MA (AC/MVK are intermediate). The time constants for the long S_1_ decay are comparable across AC/CR/MVK (∼1 ps) but a factor-of three larger in MA. Based on their TRPES-fitted sequential model, Lee et al. reported a similarly longer time constant for MA but also a distinctly shorter time constant for CR (∼500 fs) compared to those for AC and MVK.^22^ Below, we investigate the dynamical features underlying the population traces from our simulations and examine the role of inertial and electronic methylation effects at different positions in AC. Finally, we compare our findings with the original rationalization of the TRPES differences.^22^

### 3.1 S_2_ → S_1_ Deactivation

**Figure 4:**
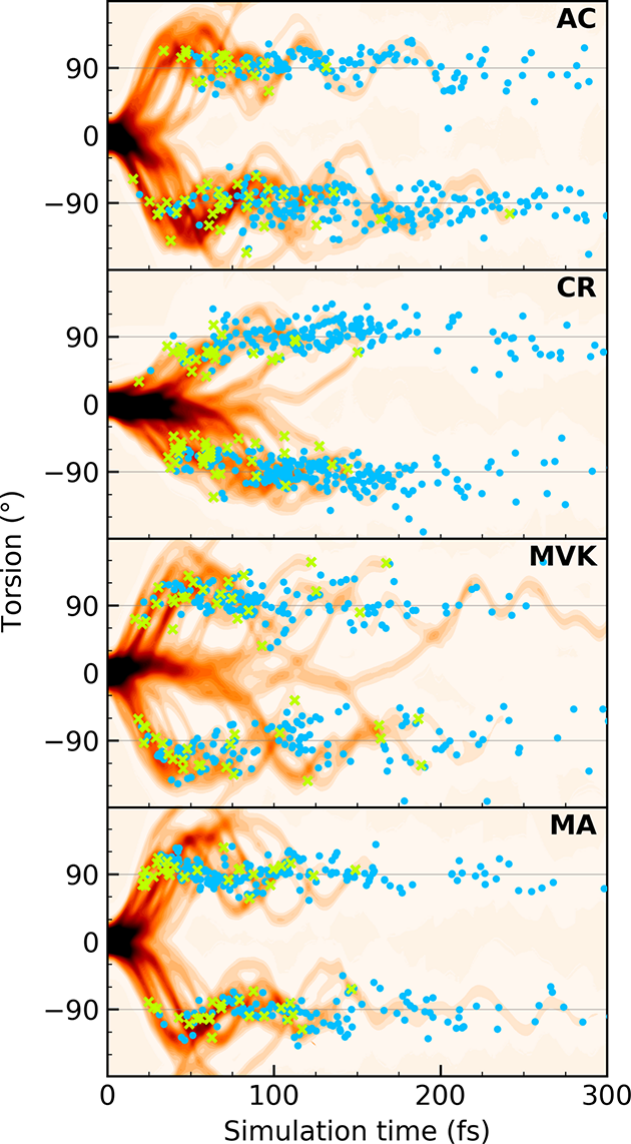
Effect of methylation on the approach to the S_2_/S_1_-intersection seam. The time evolution of the S_2_ density along the torsional mode occurred within the first 300 fs following photoexcitation. The green crosses indicate the first nonadiabatic transfer event for each IC while the blue filled circles indicate subsequent transfer events following photoexcitation. In CR, nonadiabatic transfer events are encountered earlier along the torsion.

Figure 4 shows the initial 300 fs time evolution of the S_2_ wavepacket density along the torsional and BLA modes, respectively. The green crosses indicate the first nonadiabatic transfer event for each IC while subsequent events are marked by blue filled circles. Consistent with previous studies on AC,^22,36,37,71,92^ the departure from the FC region is initiated by ultrafast BLA expansion and then proceeds along the torsion. This brings the systems into the vicinity of the S_2_/S_1_-intersection seam, marking the onset of population decay. Figure 4 already demonstrates effects of methylation: (i) The torsional motion is slowed in CR. This is expected from the higher torsional inertia imposed by the heavier methyl group that, in turn, slightly delays the onset of population decay. (ii) In AC/MVK/MA, the wavepacket significantly overshoots 90° torsional configurations following the initial approach to the intersection seam. Such large-amplitude torsional motion is absent in CR. (iii) For MVK, a small portion of the wavepacket is transiently trapped around planar configurations. This gives rise to the observed tail in the S_2_ population decay and a slightly longer S_2_ decay time (Figure 3). To understand these differences, we consider the additional modes activated during the early dynamics.

**Figure 5:**
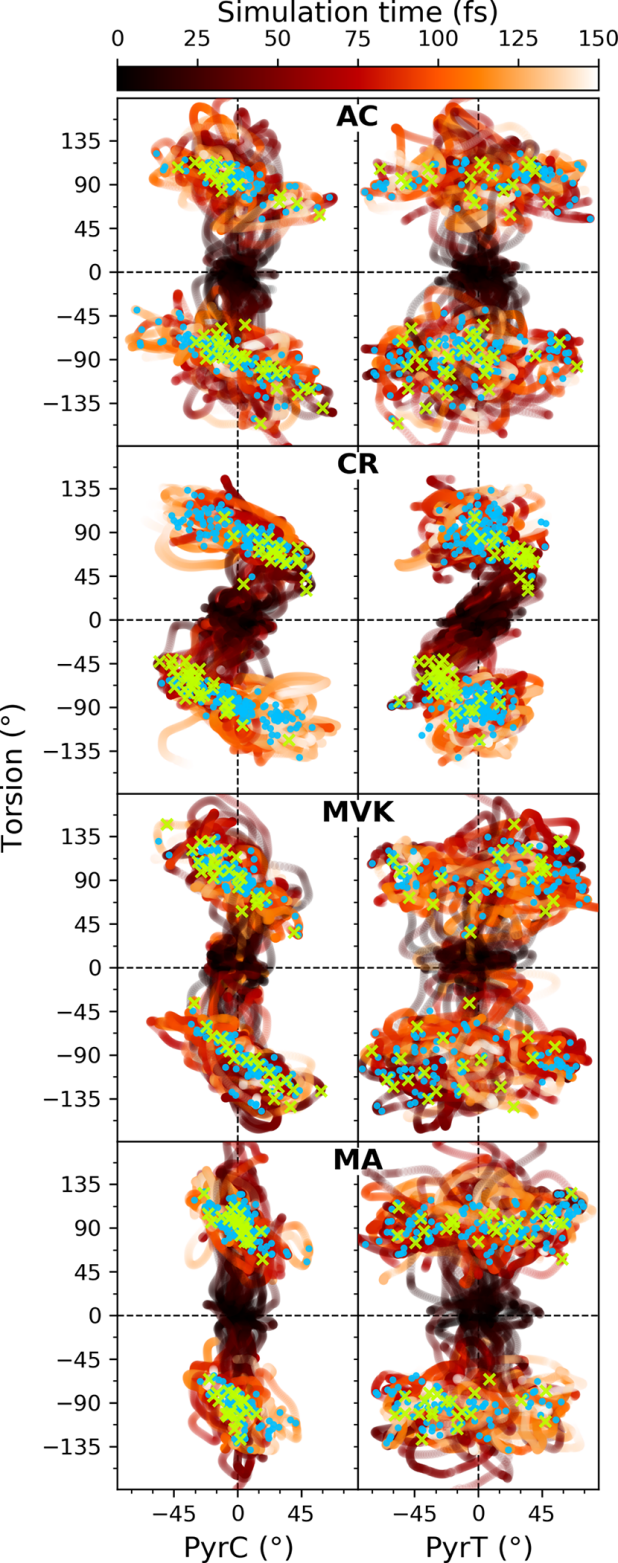
Departure from the FC point. Early time evolution (see color bar) of the centroids of the TBFs on S_2_ in the subspace spanned by PyrC and torsional modes (left panel) and PyrT and torsional modes (right panel). The green crosses indicate the first nonadiabatic transfer event for each IC while the blue filled circles indicate subsequent transfer events within the first 150 fs following photoexcitation.

Figure 5 illustrates the early time evolution (<150 fs) of the centroids of the TBFs on S_2_ in the subspace spanned by the torsional and the PyrT or PyrC modes. There is no clear correlation between PyrT and torsion in AC/MVK/MA, where substantial pyramidalization mostly ensues near ∼90°-twisted configurations. On the other hand, the extent and direction of PyrC are clearly coupled to the torsional mode at twisted configurations. In CR, such a correlation exists for both PyrT and PyrC, and it emerges already as the wavepacket leaves the FC region. The directed PyrT motion results from the asymmetric mass distribution at the terminal C atom, implying retarded torsion of the methyl group relative to the H atom. Consequently, the torsion direction dictates the PyrT direction, and the PyrC motion necessarily counterrotates to conserve total angular momentum. This correlated motion is manifested in a somewhat different decay behavior for CR, as will be discussed further below.

**Figure 6:**
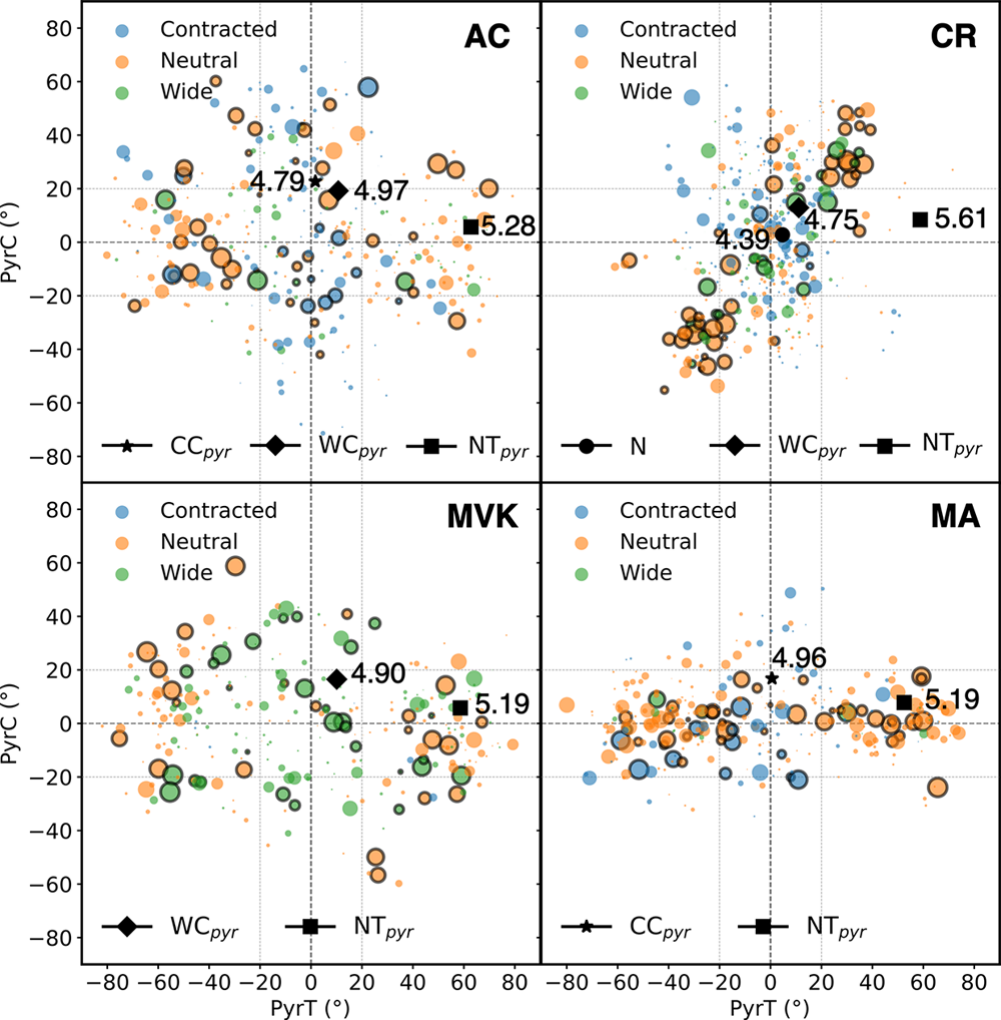
(PyrT, PyrC) distribution for all S_2_/S_1_ transition events. The area of each circle represents the absolute population transfer. Identified S_2_/S_1_-MECIs are indicated by black markers, and their energies relative to the respective S_0_-min are reported in eV. The first S_2_/S_1_ transition events for each IC are distinguished by black edge colors. The classification of the central angle into contracted (C), neutral (N), and wide (W) are indicated by the coloring. Vertical and horizontal dashed lines serve as guides for the eye.

As seen from the location of the first and subsequent nonadiabatic transitions in Figure 5 (green crosses and blue filled circles, respectively), the (un)correlated PyrT/PyrC and torsion trends also characterize the accessed part of the intersection seam. When we consider the (PyrC, PyrT) distributions of all S_2_/S_1_ nonadiabatic transfer events (Figure 6), additional differences become clear. The black markers indicate the position of the optimized MECIs in the positive quadrant. Nearly degenerate MECIs also exist (not shown) in other quadrants with all possible positive and negative combinations of PyrT and PyrC values. For MA, the nonadiabatic transfer occurs primarily for highly terminally pyramidalized geometries (|PyrT| > 20°) and neutral central angles (see coloring), i.e., mostly near MECI-NT_*pyr*_. A similar picture emerges for MVK, but with a larger spread in PyrC, as expected due to the lack of the central methyl group to restrict the motion. However, by comparison to AC, it is clear that methylation at the carbonyl C atom does impose additional constraints, influencing the accessed intersection seam. As such, AC explores both highly terminal and centrally pyramidalized geometries. It should be noted that the tighter PyrC behavior in MA is not only a result of increased inertia of the central methyl group but also due to electronic effects, as seen from a comparison between the S_2_ PES PyrC-torsion cuts around S_2_/S_1_-MECI-NT_*pyr*_ for AC and MA (Figure S14). For CR, this region of the intersection seam is dynamically inaccessible. Rather, the correlated PyrT/PyrC and torsional motion initially guides the wavepacket toward a part of the intersection seam characterized by lower torsion (<75°) and simultaneous PyrT and PyrC (green crosses and black-outlined circles in Figures 5 and 6, respectively). MECI searches starting from the associated nonadiabatic transitions end in S_2_/S_1_-MECI-NT_*pyr*_, indicating that CR accesses a higher-lying, nonstationary part of the intersection seam earlier in the dynamics. As the system approaches 90°-twisted structures, the extent of PyrT is reduced. As will be discussed later, this coordinated motion upon reaching S_1_ also promotes the initial ground-state recovery.

Next, we turn to point (ii), pertaining to the torsional overshooting in AC/MVK/MA. In line with the identified MECIs and the dynamics discussed above, reaching the intersection seam requires pyramidalization motion that is primarily activated near 90°-twisted configurations in these three systems. Together with the initial ballistic torsional behavior, this results in a first passage through methylene 90°-twisted configurations that does not efficiently induce population transfer (Figure S17). On the other hand, the combination of the correlated pyramidalization and torsional motion in CR steers the wavepacket toward the intersection seam already at partially twisted geometries. This leads to substantial population decay earlier along the torsional mode.

While the time scale for S_2_ relaxation is ultrafast across all systems, MVK exhibits a slightly slower decay. As alluded to in (iii) above, this results from a transient delay of the wavepacket around planar configurations. At the FC point, the wavepacket is directed downhill toward lower-energy regions (∼0.7 eV below the FC point), characterized by an expanded BLA, thereby generating BLA oscillations. The search for a planar S_2_ minimum from the FC point was unsuccessful in all molecules, leading to an imaginary frequency along the torsion. However, we located a shallow minimum (torsional barrier <0.05 eV as estimated by a nudged-elastic-band^93^ calculation connecting S_2_-min* and S_2_/S_1_-MECI-WC_*pyr*_) in MVK upon rotating the methyl group to align one of its H atoms with a methylene H atom (see S_2_-min* in Figures S6 and S15). Note that we could not confirm a similar structure to be a true minimum at the XMS-CASPT2 level (see Section S1 for additional details). While this could be a potential cause of transient trapping, inspection of the associated ICs in MVK indicates an alternative explanation. In particular, it suggests that the delay is induced by either an initial in-phase motion along the PyrT and PyrC modes or an overall swinging of the central C atom. Importantly, these modes both hinder progress of methylene torsion and are imprinted already in the IC sampling. Indeed, by comparing the normal modes across systems, we find two (instead of one) low-frequency in-phase modes in MVK (Figure S18). This suggests that the delay is of inertial origin rather than a consequence of a tiny potential barrier at the *hh*-TDA level.

### 3.2 S_1_ → S_0_ Deactivation

The ultrafast (∼90 fs) and longer (∼1 ps) time scale components from our S_0_ population fitting indicate that the ground-state recovery proceeds in two different regimes: (i) The first is a ballistic regime in which the wavepacket is brought to the S_1_/S_0_-intersection seam before significant intramolecular vibrational redistribution (IVR) has occurred. In this limit, the direction and velocity of the wavepacket approaching the intersection seam become key variables dictating the nonadiabatic dynamics.^94−97^ (ii) The second is an IVR-limited regime, where the wavepacket exists on S_1_ for long enough to allow for some degree of IVR. Here, the access to the intersection seam is expected to be increasingly governed by relative energetics,^97−99^ although a high excess kinetic energy can distort this picture. According to the fit amplitudes, the ultrafast decay accounts for 60–70% (AC, 70%; CR, 73%; MVK, 65%; and MA, 61%) of the ground-state recovery, with the remaining population transferring in the IVR-limited regime. Importantly, while the ultrafast S_0_ population growth is largely comparable across systems, the longer time constant is about thrice as long in MA. Below, we divide the decay into early and late time bins (fast, 0–120; slow, >120 fs) to facilitate further analysis of the dynamical behavior in these two regimes. This 120 fs threshold corresponds approximately to the time at which ∼60% of the ultrafast repopulation has taken place.

**Figure 7:**
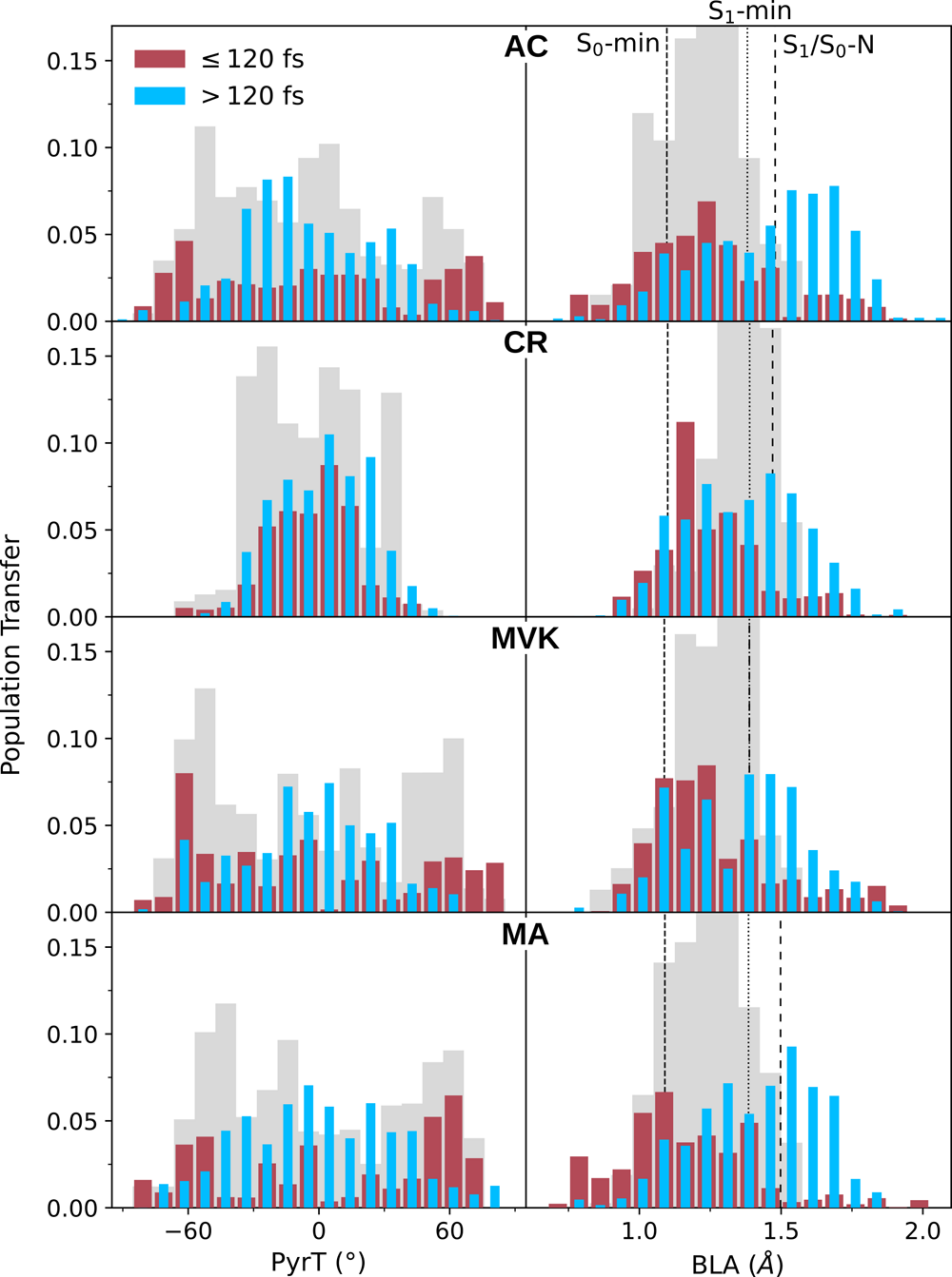
Population-weighted distribution of PyrT (left) and BLA (right) at the early (red, ≤120 fs) and late (blue, >120 fs) S_1_/S_0_ nonadiabatic transitions together with the S_2_/S_1_ counterpart (gray). Vertical lines indicate the BLA for the pertinent critical points. The early S_1_ → S_0_ decay is characterized by reduced BLA values, whereas at later times the BLA is increased toward a value characteristic of S_1_/S_0_-MECI-N. With the exception of CR, the PyrT distribution goes from a bimodal toward a broad but increasingly unimodal distribution.

To elucidate the mechanistic details underlying the ultrafast time constant of S_0_ repopulation, we examined the geometric displacements necessary to bring the wavepacket from the S_2_/S_1_-intersection seam to the regions of the S_1_/S_0_ seam accessed at early times. Figure 7 displays the population-transfer-weighted PyrT and BLA distributions of the S_1_ → S_0_ nonadiabatic transfer events together with the S_2_/S_1_ counterparts. Additional geometric parameters are provided in Figures S20 and S21. The corresponding ΔBLA and Δ|PyrT| distributions representing the changes in going from the parent S_2_/S_1_ to the pertinent S_1_/S_0_ transfer event are shown in Figure S22. For the methylated derivatives, the early S_1_ decay (red bars) is characterized by a reduction in the fast BLA mode (time period of ∼25 fs). For MA and MVK, this change represents the main displacement required to reach the S_1_/S_0_ counterparts of MECI-NT_*pyr*_, dominating the S_2_ → S_1_ decay. Although CR does not access the terminal twist-pyramidalized part of the seam, the BLA-mediated picture remains the same. This behavior is less distinct for AC, as expected from its larger variation in the S_2_/S_1_ nonadiabatic transition characteristics (Figure 6). In other words, the early decay is facilitated by the geometric and energetic proximity of a higher-lying S_1_/S_0_-intersection seam, which can be reached quickly along the fast BLA mode prior to any substantial planarization of the comparatively slower torsional mode (period, ∼100 fs (160 fs for CR)).

**Figure 8:**
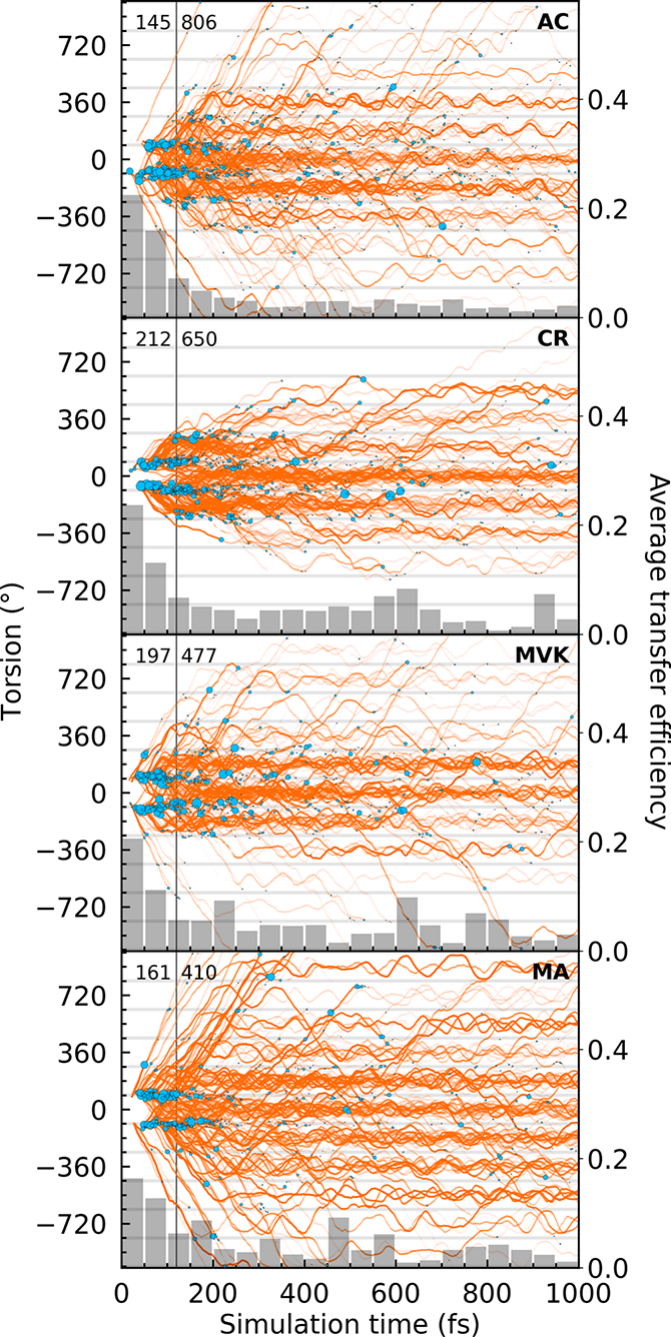
Torsional evolution (left axis) at the centroids of the TBFs on S_1_ during the first 1 ps. The line transparency is proportional to the squared amplitudes (i.e., an incoherent approximation for the TBF population). Nonadiabatic transitions to S_0_ are indicated by blue filled circles with area scaled according to the absolute population transfer. The number of nonadiabatic transfer events in the early and late time bins are shown next to the vertical 120 fs threshold line. The bar charts at the bottom report the average transfer efficiency over time (right axis). The gray-shaded horizontal regions indicate dihedral spans (2*n* + 1)90° ± 10° for integer *n*.

Beyond these similarities, there are differences in the relative importance of the ballistic component (∼10% more prominent for CR than for MA) that can all be linked to distinct dynamical behaviors. Terminal methylation promotes ballistic decay in three ways: (i) the coordinated PyrC and torsional motion directs the S_1_ wavepacket along the higher-lying part of the S_1_/S_0_-intersection seam immediately upon reaching S_1_ (see early time evolution in SI movie); (ii) the slower torsional motion extends the time window in which ballistic transfer occurs (Figure S19); and (iii) the decay to S_1_ at lower torsion and larger PyrC angles expands the accessed part of the seam. Altogether, these effects increase the population transfer (and efficiency) of the ballistic decay in the CR (gray bar charts in Figure 8). Conversely, the central methylation in MA leads to a faster and more direct torsional motion toward the S_2_/S_1_-intersection seam (Figure S19) and hence upon reaching S_1_. While this fast torsional motion accelerates the S_2_ → S_1_ decay, it impedes the ultrafast ground-state recovery by rapidly driving the wavepacket away from the S_1_/S_0_-intersection seam, instead causing initial torsional spinning (Figure 8 and SI movie). Together with the slightly delayed onset of the S_1_ → S_0_ decay, this accounts for the larger buildup of S_1_ population for MA in Figure 3. It should further be noted that the larger ballistic component in CR can explain the more pronounced biexponential decay behavior of its S_1_ population profile relative to the monoexponential decay behavior in MA (not fitted).

The portion of the wavepacket escaping nonadiabatic transfer in the ballistic regime (missing the seam or only partially transferring) proceeds primarily toward the planar S_1_ minimum. At that point, several factors can influence the subsequent decay to the ground state: (i) any remaining nonstatistical behavior; (ii) the energetic location of the S_1_/S_0_-intersection seams and their topographies; and (iii) the number of degrees of freedom and hence the available energy per vibrational degree of freedom (assuming equipartitioning). Each of these factors will be sensitive to the methylation pattern: two immediate effects are the fewer degrees of freedom in AC and the ∼0.3 eV stabilization of the lowest-energy region of the S_1_/S_0_-intersection seam by the formyl methylation in MVK discussed earlier (Figure 2). Below, we explore the origins of the similarity in long-time-scale components for ground-state recovery in AC/CR/MVK and the factor-of-three slowing in MA.

As shown in Figure 7 (blue bars), the geometries mediating the late S_1_ decay display increased BLA and reduced PyrT values, i.e., changing the largely bimodal early PyrT distributions of AC/MVK/MA toward broad unimodal distributions centered around zero. These changes are characteristic of the lowest-energy S_1_/S_0_-MECI-N, which is indeed expected to be increasingly important in the IVR-limited regime. However, the excess kinetic energy (sum of the average kinetic energy and the energy gap between the FC point and S_1_/S_0_-MECI-N) causes vigorous motion and hence a broader distribution of configurations mediating population transfer. This will inevitably complicate any analysis based on lowest-energy MECIs, particularly for AC with its fewer vibrational degrees of freedom (implying an additional 0.05–0.07 eV excess kinetic energy per degree of freedom compared to the other systems). A comparison between the temporal distributions of the S_1_/S_0_ nonadiabatic transitions and their average transfer efficiency (Figures 8 and S23) shows that AC features up to twice as many events in the late time bin (120–1000 fs) as well as the lowest transfer efficiency among all systems. Thus, it is clear that rather different behaviors underlie the comparable long time constants in AC/CR/MVK: while it is easier to reach the S_1_/S_0_-intersection seam in AC, the approach to the seam renders population transfer less effective than in CR/MVK. In passing, we note that a plateau in the S_1_/S_0_ population profiles (between 600 and 900 fs) in CR (Figure S26) contributes to a slight slowing of its longer time constant relative to MVK. In MA, the combination of fewer nonadiabatic transition events and relatively lower transfer efficiency (slightly higher than for AC) is the source of the factor-of-three longer time constant.

**Figure 9:**
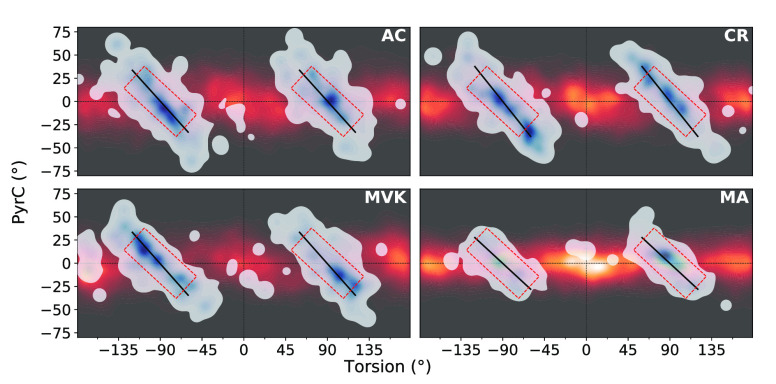
Accumulated S_1_ wavepacket density (orange contours) along the PyrC and torsional (wrapped) modes together with the distribution of S_1_/S_0_ nonadiabatic transition events (blue contours, normalized for each molecule). While these two coordinates are largely uncorrelated on S_1_, this is not the case at the nonadiabatic transitions. The accessed PyrC span is significantly smaller in MA than in the other systems. The red square indicates the angle distribution in which ∼75% of the MA population is transferred. The black lines indicate the intersection seam, as obtained by Deming regression of the nonadiabatic transfer events centered around 90° torsion.

**Figure 10:**
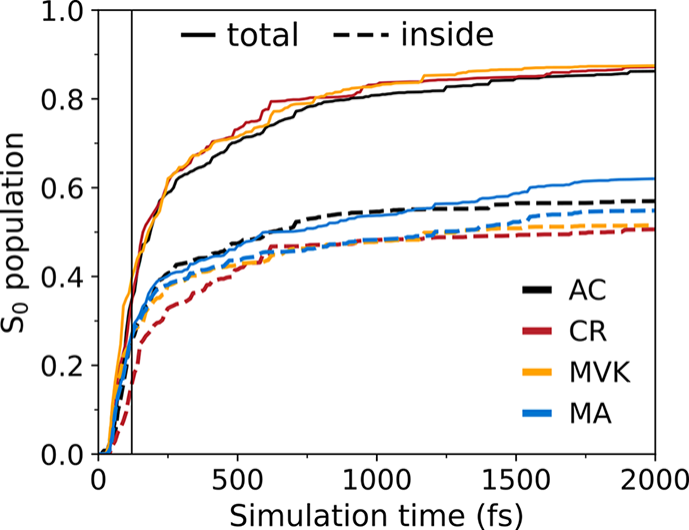
Total S_0_ population profile (solid lines) together with the contribution transferring inside (dashed) the torsion-pyramidalization range defined by the red outlined rectangles in Figure 9. As evident, the "inside" population traces are largely comparable across systems as opposed to the total. This suggests that the main factor limiting the efficiency of the late, more statistical ground-state recovery in MA is restricted access to the intersection seam.

Figure 9 shows the accumulated S_1_ wavepacket density along the PyrC and torsional modes (orange contours) together with the distribution of S_1_/S_0_ nonadiabatic transitions (blue contours). The motion along PyrC on S_1_ is much more restricted in MA. A similar trend is observed for the accessed intersection seam. Across systems, the seam is associated with correlated PyrC and torsional motion, particularly in MA. This is highlighted by the black lines in Figure 9, which was obtained from Deming regression of the nonadiabatic transitions located around 90° torsion. Together with the tighter PyrC span, this means that the population transfer occurs over a narrower effective torsional range in MA than in the other systems (torsion span around 90° encompassing 75% of the transfer events is for AC, ±30°; CR, ±29°; MVK, ±33°; and MA, ±25°). Accordingly, this suggests that central pyramidalization limits the dynamical access to the S_1_/S_0_-MECI-N part of the intersection seam, governing the late decay. Indeed, quantifying the population transfer within a similarly restricted PyrC-torsional span in AC/CR/MVK yields values comparable to those in MA (Figure 10), supporting this picture. This analysis was done by defining a rectangle enclosing 75% of the >120 fs population transfer in MA, corresponding to ∼87% along and orthogonal to the fitted black line in Figure 9, and then considering the population transfer in AC/CR/MVK within this restricted region.

To understand the origin of this central methylation effect, we compared two different S_1_ PES cuts along the PyrC and torsional modes for AC and MA: relaxed and unrelaxed scans. In the latter, all geometric parameters, except PyrC and torsion, were fixed to their S_1_/S_0_-MECI-N values (Figure S27). Two effects emerge from these results: (i) a tightening of the S_1_ potential along PyrC and (ii) a larger opening of the S_1_/S_0_-energy gap upon relaxation, effectively bringing the system farther away from the intersection seam region. The main difference between S_1_/S_0_-MECI-N and the relaxed (PyrC, torsion) counterpart is an ∼10° widening of the ∠C_2_C_1_O angle. Together with the lower kinetic energy per degree of freedom (further contributing to smaller vibrational amplitudes), these effects appear to explain the more restricted access to the intersection seam in MA with respect to AC in spite of their comparable uphill S_1_/S_0_-MECI-N energetics (Figure 2).

### 3.3 Overview of Decay Pathways and Methylation Effects

**Figure 11:**
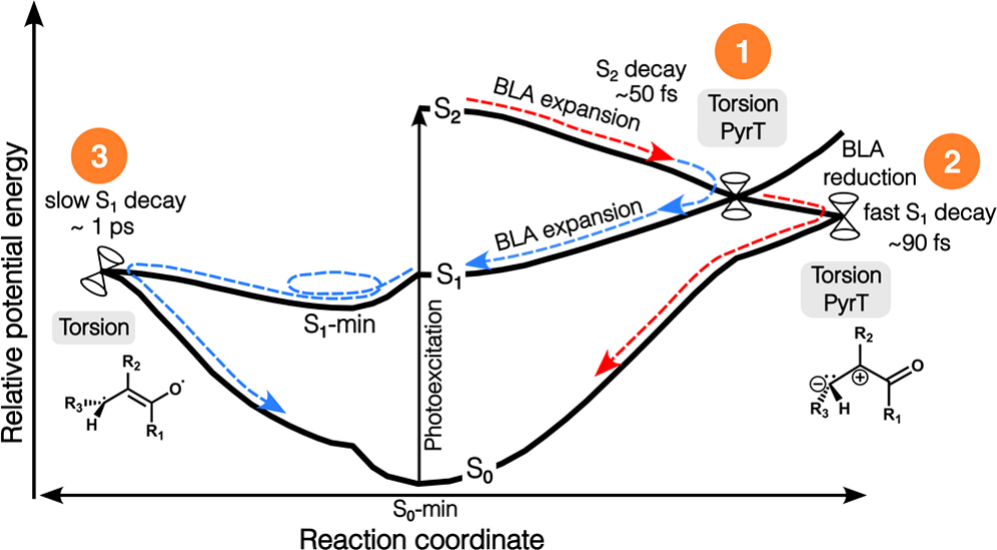
Schematic overview of the excited-state decay mechanisms as obtained from our AIMS simulations. (1), (2), and (3) denote the departure from the FC region and approach to the S_2_/S_1_-intersection seam, ballistic S_1_ → S_0_ decay, and IVR-limited S_1_ → S_0_ decay, respectively. (1) Following S_2_ photoexcitation, the wavepacket proceeds toward a terminal twist-pyramidalized intersection seam that mediates ultrafast decay on an ∼50 fs time scale. Once on S_1_, the wavepacket bifurcates: (2) more than 50% relaxes to S_0_ in a ballistic regime through a geometrically proximate S_1_/S_0_-intersection seam reached by ultrafast BLA contraction (red arrows); (3) the remainder wavepacket relaxes toward the BLA-inverted, planar S_1_ minimum (blue arrows). Ground-state recovery becomes increasingly statistical on the picosecond time scale primarily via twisted and nonpyramidalized configurations, as characteristic of the lower-energy S_1_/S_0_-intersection seam.

Figure 11 summarizes the three main features of the nonadiabatic mechanisms emerging from our simulations: (1) Following photoexcitation, the wavepacket proceeds predominantly toward a higher-lying terminal twist-pyramidalized S_2_/S_1_-intersection seam, characterized by charge-transfer across the ethylenic unit. This facilitates decay to S_1_ on the ∼50 fs time scale from which ground-state recovery proceeds along two almost equally important pathways. (2) Early ballistics is followed by (3) a picosecond time scale IVR-limited channel. Initially, prior to any significant planarization, the wavepacket reaches a higher-energy S_1_/S_0_-intersection seam by fast BLA contraction. The charge-transfer character is preserved along this pathway. The portion of the wavepacket escaping ballistic decay evolves toward the S_1_ minimum, where deactivation becomes increasingly statistical. It is dominated by torsional motion and BLA expansion (i.e., limited pyramidalization), as required to access the lowest-energy S_1_/S_0_-intersection seam. This pathway exhibits covalent character corresponding to biradicaloid species. However, it is important to note that the high excess kinetic energy upon reaching S_1_ from S_2_ leads to vigorous motion and hence a broader distribution of structures mediating population transfer. It should also be noted that the twist-pyramidalized MECIs of AC associated with charge-transfer character resemble those reported to mediate S_1_ → S_0_ decay in ethylene and butadiene.^19,90,91,100−102^ Conversely, the lowest-energy S_1_/S_0_-MECI-N exhibits different covalent character (section S5) from that reported to facilitate S_1_ → S_0_ transfer via the covalent pathway in butadiene.^19,36,90,91^ Reference (43) provides an instructive overview.

The above general picture is affected by the methylation site, as summarized below for each of the three main processes.

(1) **Departure from the FC region and approach to the S_2_/S_1_-intersection seam.** The departure of the wavepacket from the FC region is notably different for the terminally methylated CR, leading to a correlated motion among torsion, PyrT, and PyrC. This correlated motion brings the wavepacket near a higher-lying part of the S_2_/S_1_-intersection seam that is unexplored in the other systems. On the contrary, the fast and uncorrelated torsional motion in AC/MVK/MA causes inefficient population transfer during the first passage near the S_2_/S_1_-intersection seam.
(2) **BallisticS**_**1**_**→S**_**0**_**decay.** Terminal methylation also exerts distinct effects on the fast S_1_ decay. Specifically, the correlated torsional and pyramidalization motion due to terminal methylation (in CR) guides the wavepacket along the S_1_/S_0_-intersection seam in the ballistic regime. Such guided motion along the seam increases both the time window of nonadiabatic transfer and the accessed part of the seam, giving rise to the largest amplitude of ballistic decay. Conversely, central methylation in MA causes the wavepacket to quickly leave the seam through a fast torsional motion (SI movie).
(3) **IVR-limited S**_**1**_**→S**_**0**_**decay.** Central methylation in MA also slows down the decay in the IVR-limited regime by restricting the access of the wavepacket to the S_1_/S_0_-intersection seam. This is attributable to (i) an electronic component causing a tightening of the PES along PyrC and (ii) a thermal (as opposed to an inertial) component contributing to a smaller PyrC amplitude in MA due to the lower gain in excess kinetic energy per degree of freedom upon reaching S_1_-min relative to AC. Moreover, the lowest-energy S_1_/S_0_-MECI is also stabilized by ∼0.3 eV upon formyl methylation (in MVK). However, such an electronic effect is not manifested critically in the S_1_ → S_0_ deactivation following S_2_(ππ*) photoexcitation due to the large gain in excess kinetic energy upon reaching S_1_.

### 3.4 Comparison to the TRPES Experiment

Finally, we attempt to interpret the TRPES results by Lee et al.^22^ in light of the mechanistic details uncovered by our simulations. It should be noted that a strict theory–experiment comparison would require the explicit calculation of TRPES observables: the kinetic energy release in TRPES can shift due to energy stabilization/destabilization and changing state characters along the neutral and cationic pathways, implying that population traces and TRPES signals do not present a one-to-one mapping.^103−107^ Lee et al. employed both one- and two-photon ionization from the excited state (pump, 200 nm; probe, 267 nm) in order to capture the TRPES signal covering a larger range of photoelectron kinetic energies. They fitted the measured TRPES signal using a four-component sequential kinetic model (X → A → I → P) with the first component X accounting for the instrument response function (IRF ≈ 160 fs) and the remaining three corresponding to a sequential decay: an immediately excited species A decays in an ultrafast manner to an intermediate I, which then decays to a product P on the picosecond time scale. The ultrafast time constant in this model was interpreted as the departure of the wavepacket from the FC region, and the slower component was interpreted as the ground-state recovery from S_1_, possibly with some degree of intersystem crossing. While the ultrafast TPRES component is largely comparable across systems (50–190 fs), terminal and central methylation shortens and extends, respectively, the S_1_ lifetime (AC, ∼620; CR, ∼500; MVK, ∼1040; and MA, ∼1800 fs). Lee et al. rationalized these differences based on inertial effects and first-order branching space analyses of the lowest-energy MECI (labeled S_1_/S_0_-MECI-N in this work). The faster S_1_ internal conversion in the CR was proposed to originate from a slowing of the torsional motion (a seam coordinate in the linear approximation). This allows the wavepacket to stay near the intersection seam that is reached by BLA and PyrC displacements (dominating the gradient difference and nonadiabatic coupling vectors, respectively; Figure S29). Conversely, the central methyl group was conjectured to slow down motion along the PyrC-dominated nonadiabatic coupling vector, thereby reducing the effective coupling strength and in turn retarding the S_1_ → S_0_ decay.

The picture emerging from our simulations differs in central ways yet supports parts of the proposed explanations. First, our results suggest a nonsequential model involving S_1_ → S_0_ decay in both ballistic and IVR-limited regimes, with the former accounting for half or more of the decay. Second, while the simulations reproduce the slower S_1_ decay in MA, the time constants are comparable across AC/CR/MVK (in contrast to the faster CR decay found experimentally). Besides the inherent limitations in the theoretical–experimental comparison mentioned above, this difference may be explained by a combination of the sequential model assumed in the experimental fitting and the larger ultrafast ballistic component in CR (responsible for ∼70% of the decay) suggested in this work. In other words, our results indicate that the main imprint of terminal methylation on the S_1_ deactivation is to be found in the ballistic and not the IVR-limited regime. However, the mechanism underlying the more pronounced ballistic decay aligns with the original rationalization. We find that terminal methylation induces correlated torsional-PyrC motion that upon reaching S_1_ initially guides the wavepacket along a higher-lying part of the S_1_/S_0_-intersection seam. This increases the interaction time and, in turn, population transfer in the ballistic regime. Our dynamical picture for MA indicates that the slower ground-state recovery is the combined result of (i) an initial fast torsional motion that drives the system away from the higher-lying intersection seam accessed in the ballistic regime and (ii) a more limited access to the lower-lying intersection seam accessed at later times, thereby extending the longer decay constant. In other words, central methylation effects are manifested mainly through restricted seam accessibility due to a smaller PyrC amplitude rather than through a reduced transfer efficiency at the seam. Specifically, although the velocity component along the nonadiabatic coupling vector is smallest in MA, we see no clear correlation between the average population transfer and the velocity component along the nonadiabatic coupling vector in the IVR-limited regime (comparing Figures S23 and S28). Pertaining to the time constants for S_2_ relaxation, we find overall good agreement with the ultrafast TRPES components. Although the differences are small in our simulations and within the IRF in the experiment, we reproduce the slower behavior in MVK and ascribe it to a small fraction of in-phase PyrC/PyrT motion out of the FC region, delaying the torsional approach to the S_2_/S_1_-intersection seam.

An aspect not covered in the present work is the possibility of an intersystem crossing from S_1_ to the triplet manifold. Previous theoretical works indicate that triplet states are involved in the dynamics following S_1_(nπ*) photoexcitation due to their energetic proximity at the planar S_1_-min and the energetically uphill approach to the S_1_/S_0_-intersection seam.^36,37,108−110^ Upon S_2_(ππ*) photoexcitation, intersystem crossing is, however, expected to be less prominent due to both the ballistic and hot IVR-limited channels, which promote internal conversion to the ground state. However, some degree of intersystem crossing may be relevant for the longer-lived S_1_ behavior. This was conjectured based on the HCO ground-state fragments detected during the photolysis of AC and CR at 193 nm.^64,65^ However, singlet-to-triplet branching ratios remain elusive. The longer S_1_ lifetime of MA indicates that central methylation could make the system more prone to undergo intersystem crossing following S_2_(ππ*) photoexcitation. Future work considering the triplet manifold is required to resolve these questions.

## 4 Conclusions

We investigated the impact of methylation on the internal conversion dynamics of AC following photoexcitation to S_2_(ππ*). The main features of our simulations can be summarized as follows. Following photoexcitation, the wavepacket undergoes ultrafast decay (∼50 fs) to S_1_ from which ground-state recovery occurs in two different regimes: (i) ∼60–70% of the population decays in a ballistic regime mediated by ultrafast BLA motion and (ii) the part escaping ballistic decay proceeds toward the planar S_1_-min from which the IVR-limited decay takes place on the picosecond time scale. Although this general picture is largely preserved, methylation affects both time scales and underlying dynamical features.

Although methyl substitution is commonly assumed to exert inertial effects, its weak electron-donating capacity can alter the energies and topographies of the PESs. Our simulations indicate that terminal methylation (β C atom) is mainly manifested through inertial effects: it leads to correlated torsional and pyramidalization motion that guides the wavepacket along the S_1_/S_0_-intersection seam, promoting decay in the ballistic regime for CR. On the other hand, central methylation (α C atom) impedes ballistic decay by inducing fast torsional motion that brings the system away from the intersection seam. It further slows down deactivation in the IVR-limited regime consistent with previous TRPES time constants.^22^ However, according to our simulations, this is a result of restricted access to the intersection seam more so than an inefficient population transfer at the seam, as originally proposed. We attribute this to two factors: (i) an electronic component that tightens the PES along central pyramidalization and (ii) a colder "thermal" component, which reduces the amplitude of the central pyramidalization. Electronic effects of methylation are also pronounced upon changing the system to an enone (MVK), leading to an ∼0.3 eV stabilization of the lower-energy S_1_/S_0_-intersection seam. However, the dynamical implications of this change are limited in the present case of S_2_(ππ*) photoexcitation due to the large excess kinetic energy available upon reaching S_1_.

The present study lays the foundation for future work focused on elucidating new and remaining mechanistic aspects. First, our simulations suggest a nonsequential S_1_ decay as opposed to the sequential model assumed in the previous TRPES study by Lee et al.^22^ The time scale of the long S_0_-repopulation trace for CR simulations does not follow the accelerated TRPES trend obtained under this assumption. We find terminal methylation to promote decay in the ballistic regime rather than in the IVR-limited regime. Addressing this aspect will require an explicit calculation of TRPES observables and a reconsideration of the fitting model in the experiment. Second, the longer S_1_ time constant in MA suggests a potentially larger involvement of triplet states also in the dynamics initiated on S_2_, requiring an account of intersystem crossing in the simulations. Third, a different but related aspect pertains to the predicted electronic effects of formyl methylation. While the improved energetic access to the lowest-energy S_1_/S_0_-intersection seam in MVK is not significantly manifested in the present case, it could potentially have a pronounced effect on the singlet/triplet branching ratio following S_1_ photoexcitation. We hope this work will stimulate further investigations to shed light on these questions.

## Data Availability

10.5281/zenodo.7780176: xyz files for critical points at the *hh*-TDA and XMS-CASPT2 levels of theory, input files for geometry optimization and MECI calculations, and initial conditions (positions and momenta) used in the AIMS dynamics.

## Supporting Information Available

Accumulated S_1_ population along the PyrC or PyrT and torsional modes over time (red contours), together with the accumulated distribution of S_1_/S_0_ nonadiabatic transfer events (blue contours) (MP4)
Validation of *hh*-TDA against XMS-CASPT2, MECI nomenclature, geometric parameters and relative energies at critical-point geometries, critical-point analysis, adiabatic population fitting, and additional supporting analyses (PDF)
