# Supplementary material for: Deciphering Methylation Effects on S2(ππ*) Internal Conversion in the Simplest Linear α,β-Unsaturated Carbonyl
Source: J Phys Chem A. 2023 Jun 18;127(25):5360–73. doi: 10.1021/acs.jpca.3c02582 (PMC10316407; doi:10.1021/acs.jpca.3c02582)
Supplement: Supplementary file 2 — jp3c02582_si_002.pdf [file jp3c02582_si_002.pdf]

Supporting Information: Deciphering Methylation Effects on S$_2(\pi\pi^*)$ Internal Conversion in the Simplest Linear α,β-Unsaturated Carbonyl

Pratip Chakraborty, Rafael C. Couto, and Nanna H. List*

Department of Chemistry, KTH Royal Institute of Technology, SE-10044 Stockholm, Sweden

E-mail: nalist@kth.se

Contents

S1 Validation of *hh*-TDA against XMS-CASPT2 **3**

S2 IC convergence analysis **9**

S3 Geometric parameters **10**

S4 MECI nomenclature **17**

S5 Critical point analysis **17**

S6 Adiabatic population fitting **21**

S7 Additional Analyses **29**

References **48**

S1 Validation of *hh*-TDA against XMS-CASPT2

To validate the *hh*-TDA-ωPBEh/6-31G(d,p) results used in the non-adiabatic dynamics simulations, we compared selected critical points to counterparts computed using extended-multistate complete active space second-order perturbation theory (XMS-CASPT2).[1–3] Extensive analysis of the active space, imaginary level shift and basis set was performed to obtain the best combination in terms of accuracy and efficiency. As reported by previous calculations on acrolein (AC) and briefly summarized below, the electronic structure of the valence-excited-states of the α,β-unsaturated carbonyls present a few challenges. At the Franck–Condon (FC) point, the S_1 and S_2 states have $n\pi^*$ and $\pi\pi^*$ character, respectively. Capturing the S_1 state does not pose a challenge, as little interaction with other states is observed. However, complications arise for S_2 due to possible mixing with doubly-excited and Rydberg states. This leads to inconsistencies across different levels of theory.

The most extensive study so far was presented by Aquilante *et al.*,[4] in which a larger number of excited-states were characterized for both *s-trans*-AC and *s-cis*-AC. To this end, they performed MS-CASPT2 calculations with carefully selected active spaces to capture specific excited-states, different numbers of roots, C_s symmetry and a specially designed basis set to capture Rydberg states. Their analysis showed that S_1-S_4 are valence states, with the higher-energy states being of Rydberg character. Due to strong valence-Rydberg mixing at the CASSCF level, $S_2(\pi\pi^*)$ is located at higher energy than S_3, giving a wrong energetic ordering. This is only corrected when the reference states interact at the MS-CASPT2 level, restoring the correct energy order and bringing it closer to experimental values. This behavior was also observed and briefly discussed by Loos *et al.*[5] While their focus was on the doubly-excited ($\pi\pi \rightarrow \pi^*\pi^*$) state, the $S_2(\pi\pi^*)$ state was also characterized by a wide range of second-order perturbation theory flavors (MS-CASPT2, XMS-CASPT2, PC-NEVPT2, SC-NEVPT2), complete active spaces (CAS) and basis sets. The excited-states of AC have also been computed using symmetry adapted cluster configuration interaction (SAC-CI) level,[6] different coupled-cluster methods (CC3 and CCSDT) and full-CI by Loos *et*

al.[7] In the latter, attempts were made at the CCSDTQ level, but due to strong state mixing, convergence was not reached. These two studies focused on benchmarking of AC's vertical excitation energies. Regarding the geometric parameters of AC, extensive work has been done considering CC, CASPT2 and TD-DFT methods.[8,9] However, due to the inaccuracy in the experimental data considered[10] a final call on the most adequate level of theory could not be done.

The studies mentioned above involving CASSCF references,[4,5,8,9] encompass a wide variety of active spaces. Aquilante *et al.* considered five different active spaces, targeting different electronic states.[4] Loos *et al.* considered two active spaces: CAS(4e,4o) and CAS(10e,10o).[5] The first contained the π_{CC}, π_{CO}, π^*_{CC} and π^*_{CO} orbitals, while the later additionally included the σ_{CC}, σ_{CO}, σ^*_{CC} and σ^*_{CO} orbitals (two $\sigma_{\mathrm{CC}}/\sigma^*_{\mathrm{CC}}$ sets). Loos *et al* also reported the use of a CAS(12e,12o) space, containing all orbitals from CAS(10e,10o) with the addition of n and Rydberg 3s orbitals, and a CAS(12e,13o), adding the Rydberg 3p orbital to the previous.[7] A few other studies on AC also considered different active spaces. Bouabça *et al.* considered the smallest active space for modeling the $n\pi^*$ state: CAS(6e,5o), including π_{CC}, π_{CO}, n_{CO}, π^*_{CC} and π^*_{CO} orbitals.[11] Fang considered a CAS(8e,7o) space which additionally included the σ_{CC} and σ^*_{CC} orbitals.[12] Bokareva *et al.* employed three different active spaces: CAS(6e,5o) as before, CAS(8e,7o) but with σ_{CO} and σ^*_{CO}, and CAS(12e,11o) which includes the previous CAS(8e,7o) and two $\sigma_{\mathrm{CC}}/\sigma^*_{\mathrm{CC}}$ sets.[13]

Given our focus on S_0 through S_2, we performed a series of single point calculations at the FC point to determine the best combination of basis set and active space size. Thus, we considered the cc-pVDZ,[14] cc-pVTZ and aug-cc-pVDZ basis sets together with CAS(6e,5o), CAS(8e,7o), CAS(10e,9o), CAS(10e,10o), CAS(12e,11o) and CAS(12e,12o). Figure S1 shows the orbitals for the CAS(10e,9o) space, which encapsulates the CAS(8e,7o) (without the $\sigma_{\mathrm{C-C}}/\sigma^*_{\mathrm{C-C}}$ pair) and CAS(6e,5o) (without the $\sigma_{\mathrm{C-C}}/\sigma^*_{\mathrm{C-C}}$ and $\sigma_{\mathrm{C=O}}/\sigma^*_{\mathrm{C=O}}$ pairs). The bigger active spaces further included: a Rydberg 3s for the CAS(10e,10o) space, the $\sigma_{\mathrm{C=C}}/\sigma^*_{\mathrm{C=C}}$ pair for CAS(12e,11o), and the CAS(12e,12o) would include the previous space along with

the Rydberg 3s. Furthermore, a state-averaging over five roots (SA5) is required because, as mentioned above, CASSCF predicts the wrong energetic ordering, predicting the lowest $\pi\pi^*$ state to be the fifth root, which is later corrected by XMS-CASPT2. No IPEA shift was applied. We systematically tested the influence of the imaginary level shift on the excitation energies and reference weights and found that a value of 0.3 is required to avoid intruder states issues. Based on these analyses, we found that SA5-XMS(Im=0.3)-CASPT2(10e,9o)/cc-pVDZ provides the best compromise between accuracy, computational time and stability. The smaller CAS(6e,5o) and CAS(8e,7o) spaces lead to big differences in reference weights for the S_0-S_2 states in the perturbation step and display high sensitivity to the choice of imaginary shift. The chosen CAS(10e,9o) performed comparably to the bigger CAS(12e,11o) and CAS(12e,12o). Accordingly, geometry optimizations and minimum energy conical intersection (MECI) calculations for all four α,β-unsaturated carbonyls were performed at the SA5-XMS(Im=0.3)-CASPT2(10,9)/cc-pVDZ level along with the cc-pVDZ-jkfit density-fitting basis and the multi-state multi-reference contraction scheme.[15] Specifically, we considered the S_0 and S_1 minima as well as S_0/S_1-MECI-N (Sections S4 and S5). Several unsuccessful attempts were made to optimize a planar S_2 minimum of MVK at the XMS-CASPT2 level. Hence, we cannot confirm the S_2-minimum (labeled S_2-min*) found at the *hh*-TDA/6-31G(d,p) level of theory (Section S5). Geometry optimizations and frequency analyses were performed with the BAGEL program[16,17] and initial test calculations with OpenMolcas.[18,19]

Table S1 compares the excitation energies obtained at the XMS-CASPT2 and *hh*-TDA-ωPBEh levels. At the FC point (S_0-min), we find *hh*-TDA to overestimate the excitation energies for both $S_0 \rightarrow S_1$ and $S_0 \rightarrow S_2$ transitions by $\sim$0.2-0.4 eV, leading to comparatively smaller methylation effects. However, we find the same red-shifting trends for both levels of theory. For the $S_0 \rightarrow S_1$ energies, the trend is AC<CR$\sim$MVK<MA and MA<CR<MVK<AC for $S_0 \rightarrow S_2$. We also find good agreement in terms of relative energies across systems at S_1-min. When comparing the S_1-S_2 energy gaps at S_0-min and S_1-min, the differences between

the two methods are <0.1 eV. Furthermore, the methods provide similar energies for S_1/S_0-MECI-N, with differences <0.1 eV. In particular, the ~0.3 eV stabilizing effect of formyl methylation (MVK) is consistent across the two levels. In addition to the energy analysis, we compared geometric parameters for the critical points at the XMS-CASPT2 and *hh*-TDA levels (Table S2). In general, both methods agree quite well: the differences in bond lengths throughout all minima and MECI structures are smaller than 0.05 Å. The differences in torsion and angles at the minima amounts to <4°, while somewhat bigger differences appear at S_1/S_0-MECI-N: ~8° for $\angle C_2C_1O$ and up to 4° for $\angle C_1C_2C_3$. A small difference in PyrT is also seen for the MECI structures, with the biggest being of 11° for MA.

Hence, we conclude that the critical point energies and geometries obtained at the *hh*-TDA/6-31G(d,p) level are in good agreement with the XMS(Im=0.3)-CASPT2(10,9)/cc-pVDZ reference level.

Table S1: Energies for critical points of AC, CR, MA and MVK, obtained at the SA5-XMS(Im=0.3)-CASPT2(10,9)/cc-pVDZ and *hh*-TDA-ωPBEh/6-31G(d,p) levels of theory. Energies (eV) are reported relative to the ground-state energy at S_0-min for each system.

		hh-TDA			XMS-CASPT2		
	Type	S_0	S_1	S_2	S_0	S_1	S_2
AC	S_0-min	0.000	3.850	6.913	0.000	3.636	6.706
	S_1-min	0.696	3.232	6.473	0.568	3.177	6.408
	S_1/S_0-MECI-N‡	3.908	3.908	5.718	4.023	4.023	6.210
CR	S_0-min	0.000	3.966	6.813	0.000	3.715	6.435
	S_1-min	0.723	3.311	6.341	0.561	3.253	6.356
	S_1/S_0-MECI-N‡	3.986	3.986	5.226	4.037	4.038	5.622
MVK	S_0-min	0.000	3.978	6.873	0.000	3.680	6.609
	S_1-min	0.790	3.315	6.408	0.636	3.204	6.358
	S_1/S_0-MECI-N‡	3.715	3.715	5.624	3.641	3.641	5.986
MA	S_0-min	0.000	4.037	6.720	0.000	3.724	6.407
	S_1-min	0.749	3.385	6.507	0.559	3.282	6.405
	S_1/S_0-MECI-N‡	4.125	4.125	6.102	4.066	4.066	7.631

‡Not confirmed to be minima on the intersection seam.

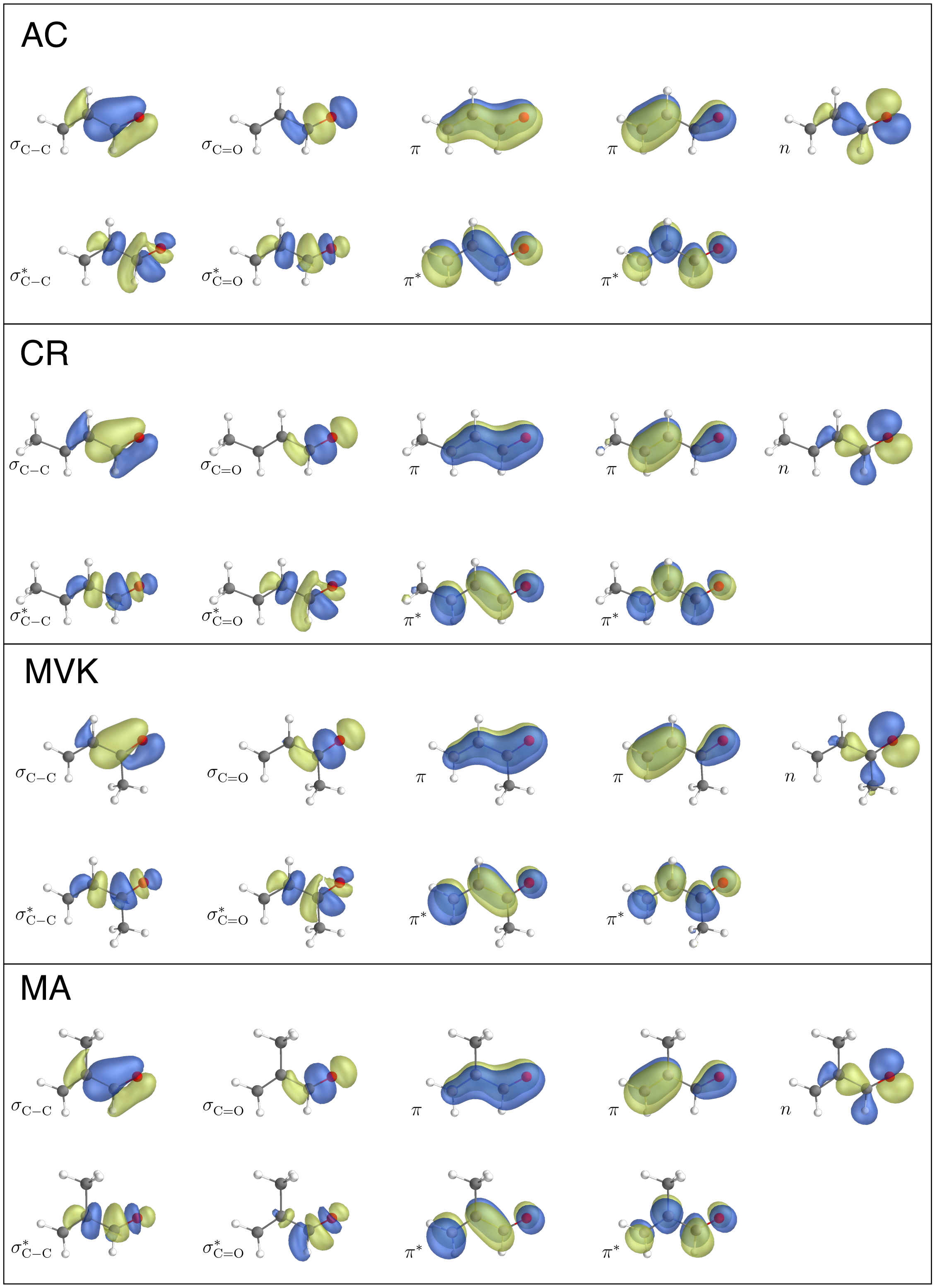

Figure S1: Natural orbitals used in the reference SA5-XMS-CASPT2(10,9)/cc-pVDZ calculations, including the $\sigma_{\text{C-C}}$, $\sigma^*_{\text{C-C}}$, $\sigma_{\text{C=O}}$, $\sigma^*_{\text{C=O}}$, π and π^* orbitals associated with the C=C and C=O bonds and the lone pair (n) on the O atom.

Table S2: Selected geometric parameters for critical points for AC, CR, MA and MVK, optimized at the SA5-XMS(Im=0.3)-CASPT2(10,9)/cc-pVDZ and *hh*-TDA-ωPBEh/6-31G(d,p) levels of theory. Distances are given in Ångström and angles and pyramidalization (PyrX) in degrees with definitions provided in Figure S3.

	**AC**					
	**S_0-min**		**S_1-min**		**S_1/S_0-MECI-N**‡	
Type	*hh*-TDA	XMS-CASPT2	*hh*-TDA	XMS-CASPT2	*hh*-TDA	XMS-CASPT2
$R(C_1{=}O)$	1.215	1.225	1.320	1.342	1.324	1.318
$R(C_1{-}C_2)$	1.452	1.488	1.348	1.399	1.315	1.334
$R(C_2{=}C_3)$	1.334	1.355	1.409	1.403	1.470	1.474
BLA	1.097	1.092	1.381	1.346	1.479	1.458
torsion	0.0	0.0	0.0	0.0	-89.9	-90.0
$\angle C_1C_2C_3$	120.5	120.8	124.4	123.2	126.1	127.2
$\angle C_2C_1O$	124.0	124.0	126.5	125.0	134.9	143.1
PyrC=O	0.0	0.0	0.0	0.0	0.0	0.0
PyrC	0.0	0.0	0.0	0.0	-0.1	0.0
PyrT	0.0	0.0	0.0	0.0	3.8	5.8
	**CR**					
	**S_0-min**		**S_1-min**		**S_1/S_0-MECI-N**‡	
Type	*hh*-TDA	XMS-CASPT2	*hh*-TDA	XMS-CASPT2	*hh*-TDA	XMS-CASPT2
$R(C_1{=}O)$	1.218	1.225	1.326	1.344	1.321	1.314
$R(C_1{-}C_2)$	1.452	1.485	1.347	1.399	1.320	1.336
$R(C_2{=}C_3)$	1.334	1.357	1.411	1.405	1.469	1.476
BLA	1.100	1.097	1.389	1.350	1.470	1.454
torsion	0.0	0.0	0.0	0.9	-89.8	-88.8
$\angle C_1C_2C_3$	121.5	120.9	125.6	123.6	126.6	126.4
$\angle C_2C_1O$	123.9	124.2	126.3	124.8	134.7	143.5
PyrC=O	0.0	0.0	0.0	0.1	0.0	0.4
PyrC	0.0	0.	0.0	-0.1	0.6	-0.4
PyrT	0.0	0.0	0.0	0.2	-0.5	9.4
	**MVK**					
	**S_0-min**		**S_1-min**		**S_1/S_0-MECI-N**‡	
Type	*hh*-TDA	XMS-CASPT2	*hh*-TDA	XMS-CASPT2	*hh*-TDA	XMS-CASPT2
$R(C_1{=}O)$	1.219	1.231	1.327	1.346	1.291	1.279
$R(C_1{-}C_2)$	1.466	1.497	1.352	1.400	1.353	1.375
$R(C_2{=}C_3)$	1.334	1.355	1.413	1.403	1.451	1.456
BLA	1.088	1.088	1.388	1.350	1.388	1.359
torsion	0.0	0.0	0.0	2.8	90.1	89.9
$\angle C_1C_2C_3$	125.2	124.7	125.8	125.2	123.3	120.6
$\angle C_2C_1O$	119.5	119.6	123.1	121.3	129.8	137.6
PyrC=O	0.0	0.0	0.8	-1.9	0.0	0.0
PyrC	0.0	0.0	-0.1	0.0	-0.1	-0.1
PyrT	0.0	0.0	-0.4	0.8	-3.3	-5.5
	**MA**					
	**S_0-min**		**S_1-min**		**S_1/S_0-MECI-N**‡	
Type	*hh*-TDA	XMS-CASPT2	*hh*-TDA	XMS-CASPT2	*hh*-TDA	XMS-CASPT2
$R(C_1{=}O)$	1.216	1.225	1.323	1.344	1.335	1.332
$R(C_1{-}C_2)$	1.462	1.493	1.351	1.407	1.308	1.326
$R(C_2{=}C_3)$	1.337	1.356	1.413	1.401	1.472	1.497
BLA	1.090	1.089	1.385	1.339	1.498	1.503
torsion	0.0	0.0	0.0	0.0	-90.0	-90.0
$\angle C_1C_2C_3$	117.9	118.2	121.6	120.8	127.1	122.6
$\angle C_2C_1O$	124.1	123.5	127.5	123.5	136.0	145.6
PyrC=O	0.0	0.0	0.0	0.0	0.0	0.0
PyrC	0.0	0.0	0.0	0.2	0.0	0.0
PyrT	0.0	0.0	0.0	-0.2	4.3	15.6

‡Not confirmed to be minima on the intersection seam.

S2 IC convergence analysis

To test the convergence of the AIMS simulations with respect to the number of initial conditions (ICs), we examined the effects of adding ICs (in increments of ten) on the population traces of CR. As seen in Figure S2, the population profiles are largely converged at 50 ICs, although error bars (not shown) will naturally be reduced.

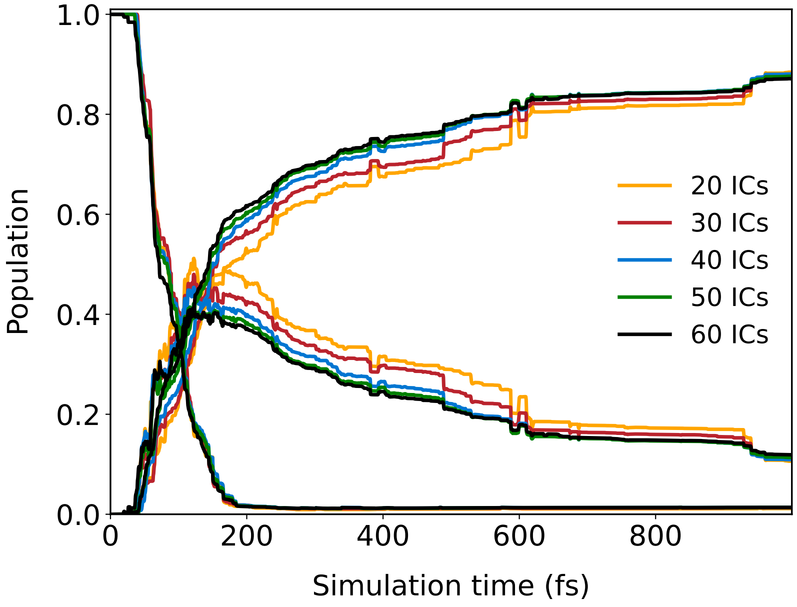

Figure S2: Convergence of the population traces with respect to the number of ICs for CR.

S3 Geometric parameters

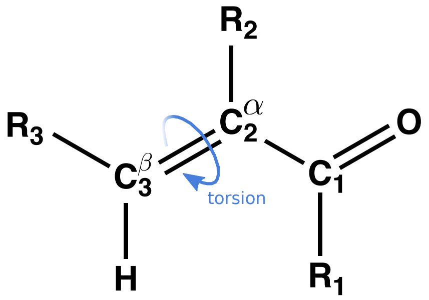

Definition of key geometric parameters

$$\mathrm{PyrC} = \arccos\left(\left(\mathbf{e}_{\mathrm{C_1\text{-}C_2}} \times \mathbf{e}_{\mathrm{C_3\text{-}C_2}}\right) \cdot \mathbf{e}_{\mathrm{R_2\text{-}C_2}}\right) - \frac{\pi}{2}$$

$$\mathrm{PyrT} = \arccos\left(\left(\mathbf{e}_{\mathrm{C_2\text{-}C_3}} \times \mathbf{e}_{\mathrm{H\text{-}C_3}}\right) \cdot \mathbf{e}_{\mathrm{R_3\text{-}C_3}}\right) - \frac{\pi}{2}$$

$$\mathrm{PyrC{=}O} = \arccos\left(\left(\mathbf{e}_{\mathrm{O\text{-}C_1}} \times \mathbf{e}_{\mathrm{R_1\text{-}C_1}}\right) \cdot \mathbf{e}_{\mathrm{C_2\text{-}C_1}}\right) - \frac{\pi}{2}$$

$$\mathrm{torsion} = \mathrm{sgn}_{\mathrm{torsion}} \cdot \arccos\left(\left(\mathbf{e}_{\mathrm{C_1\text{-}C_2}} \times \mathbf{e}_{\mathrm{C_3\text{-}C_2}}\right) \cdot \left(\mathbf{e}_{\mathrm{C_3\text{-}C_2}} \times \mathbf{e}_{\mathrm{R_3\text{-}H}}\right)\right)$$

$$\mathrm{sgn}_{\mathrm{torsion}} = \mathrm{sgn}\left(\left(\left(\mathbf{e}_{\mathrm{C_1\text{-}C_2}} \times \mathbf{e}_{\mathrm{C_3\text{-}C_2}}\right) \cdot \left(\mathbf{e}_{\mathrm{C_3\text{-}C_2}} \times \mathbf{e}_{\mathrm{R_3\text{-}H}}\right)\right) \cdot \mathbf{e}_{\mathrm{C_3\text{-}C_2}}\right)$$

$$\mathrm{BLA} = r_{\mathrm{O\text{-}C_1}} - r_{\mathrm{C_1\text{-}C_2}} + r_{\mathrm{C_2\text{-}C_3}}$$

Figure S3: Atom numbering and definitions of key geometric parameters for the α,β-unsaturated carbonyls (AC: $R_{1,2,3}$=H; CR: $R_{1,2}$=H, R_3=CH_3; MVK: $R_{2,3}$=H, R_1=CH_3; MA: $R_{1,3}$=H, R_2=CH_3). Here, $\mathbf{e}_{\text{Y-X}}$ denotes a unit vector pointing along the bond from atom X to atom Y. Sign factors are given by the projection of the cross product of the normal vectors along the central bond. The pyramidalization angles PyrX are presented for: X=C representing the central carbon C_2 (α), X=T the terminal carbon C_3 (β), and X=(C=O) the carbonyl carbon C_1. With this definition of pyramidalization, an idealized sp^2 C-atom would give a 0° while an idealized sp^3 C-atom, as in methane, corresponds to 55°.

Table S3: Selected geometric parameters for critical points for AC optimized at the *hh*-TDA-ωPBEh/6-31G(d,p) level of theory. Distances and BLA are given in Ångström and angles, dihedrals and pyramidalization in degrees with definitions provided in Figure S3. (180°) refers to geometries in which the formyl group has rotated.

Type	$R(C_1{=}O)$	$R(C_1{-}C_2)$	$R(C_2{=}C_3)$	BLA	torsion	$\angle C_1C_2C_3$	$\angle C_2C_1O$	PyrC=O	PyrC	PyrT
S_0-min	1.215	1.452	1.334	1.097	0.0	120.5	124.0	0.0	0.0	0.0
S_1-min	1.320	1.348	1.409	1.381	0.0	124.4	126.5	0.0	0.0	0.0
S_1-min(180°)	1.305	1.356	1.399	1.348	-0.1	129.0	131.8	0.0	0.0	0.0
S_2-min(tw-py)	1.235	1.436	1.399	1.198	-90.0	121.7	122.2	0.0	0.0	-61.5
				S_1/S_0-MECIs‡						
N	1.324	1.315	1.470	1.479	-89.9	126.1	134.9	0.0	-0.1	3.8
WT_{pyr}(180°)	1.283	1.341	1.456	1.398	-90.0	130.4	140.9	0.0	0.0	15.9
NC_{pyr}(180°)	1.209	1.451	1.388	1.146	-72.1	116.3	135.4	-3.9	-28.2	8.0
NC_{pyr}	1.211	1.446	1.385	1.150	-124.4	126.7	130.1	-7.0	41.0	0.8
WT_{pyr}	1.364	1.292	1.414	1.486	-90.0	152.5	107.7	0.0	0.0	-17.3
NT_{pyr}	1.221	1.455	1.392	1.157	-104.4	121.3	121.5	0.0	4.8	-61.9
				S_2/S_1-MECIs‡						
CC_{pyr}	1.229	1.384	1.415	1.260	-79.7	104.6	130.6	1.2	-22.7	1.7
WC_{pyr}	1.268	1.335	1.391	1.323	-118.6	148.9	119.6	-1.9	19.2	-10.7
NT_{pyr}	1.249	1.411	1.410	1.248	-79.2	120.4	119.7	-8.5	-5.7	-62.8

‡Not confirmed to be minima on the intersection seam.

Table S4: Selected geometric parameters for critical points for CR optimized at the *hh*-TDA-ωPBEh/6-31G(d,p) level of theory. Distances and BLA are given in Ångström and angles, dihedrals and pyramidalization in degrees with definitions provided in Figure S3.

Type	$R(C_1{=}O)$	$R(C_1{-}C_2)$	$R(C_2{=}C_3)$	BLA	torsion	$\angle C_1C_2C_3$	$\angle C_2C_1O$	PyrC=O	PyrC	PyrT
S_0-min	1.218	1.452	1.334	1.100	0.0	121.5	123.9	0.0	0.0	0.0
S_1-min	1.326	1.347	1.411	1.389	0.0	125.6	126.3	0.0	0.0	0.0
S_1-min(180°)	1.310	1.356	1.402	1.356	0.0	130.1	131.8	0.0	0.0	0.0
S_2-min(tw-py)	1.240	1.430	1.406	1.216	-75.0	122.5	122.2	-0.5	-3.9	-57.3
				S_1/S_0-MECIs‡						
NT_{pyr}(180°)	1.239	1.405	1.439	1.273	-97.9	119.8	126.7	-1.4	4.6	21.6
N(180°)	1.256	1.378	1.451	1.329	-89.4	113.9	120.9	-0.2	0.6	1.4
NC_{pyrL}	1.224	1.422	1.403	1.206	-106.5	119.6	127.3	0.3	19.1	14.3
NT_{pyrL}	1.311	1.327	1.465	1.449	-94.1	127.0	134.9	0.4	1.3	15.9
N	1.321	1.320	1.469	1.470	-89.8	126.6	134.7	0.0	0.6	-0.5
NC_{pyrH}	1.218	1.437	1.394	1.175	-66.3	123.8	127.0	4.7	-29.9	0.4
WT_{pyr}	1.351	1.298	1.413	1.466	-89.8	151.5	109.5	0.0	0.9	-14.2
NT_{pyrH}	1.227	1.445	1.401	1.182	-67.2	122.4	121.6	0.2	-5.6	-50.6
				S_2/S_1-MECIs‡						
N	1.247	1.355	1.445	1.337	86.6	112.8	132.2	-0.3	2.8	4.7
WC_{pyr}	1.275	1.329	1.405	1.351	72.3	145.1	118.2	-1.1	12.9	11.1
NT_{pyr}	1.254	1.404	1.423	1.273	-65.7	121.4	120.3	-6.5	-8.4	-58.8

‡Not confirmed to be minima on the intersection seam.

Table S5: Selected geometric parameters for critical points for MVK optimized at the *hh*-TDA-ωPBEh/6-31G(d,p) level of theory. Distances and BLA are given in Ångström and angles, dihedrals and pyramidalization in degrees with definitions provided in Figure S3.

Type	$R(C_1{=}O)$	$R(C_1{-}C_2)$	$R(C_2{=}C_3)$	BLA	torsion	$\angle C_1C_2C_3$	$\angle C_2C_1O$	PyrC=O	PyrC	PyrT
S_0-min	1.219	1.466	1.334	1.088	0.0	125.2	119.5	0.0	0.0	0.0
S_1-min	1.327	1.352	1.413	1.388	0.0	125.8	123.1	0.8	-0.1	-0.4
S_1-min(180°)	1.312	1.360	1.401	1.353	-0.1	129.7	127.7	0.0	0.0	0.0
S_2-min(tw-py)	1.236	1.453	1.397	1.180	-90.0	123.4	119.1	0.0	0.0	-56.2
S_2-min*†	1.400	1.361	1.425	1.464	0.0	125.6	123.3	0.0	0.0	0.0
				S_1/S_0-MECIs‡						
N	1.291	1.353	1.451	1.388	90.1	123.3	129.8	0.0	-0.1	-3.3
WT_{pyr}	1.371	1.295	1.423	1.498	-90.2	151.7	107.4	0.4	0.2	-18.1
NT_{pyr}	1.221	1.476	1.389	1.134	-98.5	123.7	117.8	-0.5	2.8	-67.8
				S_2/S_1-MECIs‡						
WC_{pyr}	1.270	1.344	1.396	1.322	114.2	148.8	118.4	1.8	-16.5	10.1
NT_{pyr}	1.257	1.423	1.411	1.246	-100.5	122.7	115.3	6.4	5.8	-58.6

‡Not confirmed to be minima on the intersection seam.

†A similar planar geometry could not be confirmed a true minimum at the SA5-XMS(Im=0.3)-CASPT2(10,9)/cc-pVDZ level.

Table S6: Selected geometric parameters for critical points for MA optimized at the *hh*-TDA-ωPBEh/6-31G(d,p) level of theory. Distances and BLA are given in Ångström and angles, dihedrals and pyramidalization in degrees with definitions provided in Figure S3.

Type	$R(C_1{=}O)$	$R(C_1{-}C_2)$	$R(C_2{=}C_3)$	BLA	torsion	$\angle C_1C_2C_3$	$\angle C_2C_1O$	PyrC=O	PyrC	PyrT
S_0-min	1.216	1.462	1.337	1.090	0.0	117.9	124.1	0.0	0.0	0.0
S_1-min	1.323	1.351	1.413	1.385	0.0	121.6	127.5	0.0	0.0	0.0
S_1-min(180°)	1.309	1.362	1.397	1.344	0.0	125.2	131.6	0.0	0.0	0.0
S_2-min(tw-py)	1.234	1.454	1.408	1.188	-90.1	118.4	123.7	0.0	0.4	-52.6
				S_1/S_0-MECIs‡						
N	1.335	1.308	1.472	1.498	-90.0	127.1	136.0	0.0	0.0	4.3
W	1.274	1.355	1.403	1.323	90.0	143.2	124.1	0.0	0.0	3.9
C	1.262	1.325	1.494	1.431	90.1	105.1	142.6	0.0	0.0	7.3
NT_{pyr}	1.211	1.491	1.400	1.120	-85.4	120.8	123.5	-1.6	0.0	72.9
				S_2/S_1-MECIs‡						
CC_{pyr}	1.235	1.379	1.419	1.275	-81.5	104.2	129.9	1.0	-16.8	0.6
NT_{pyr}	1.252	1.419	1.442	1.275	-103.1	119.0	119.2	10.6	7.8	52.5

‡Not confirmed to be minima on the intersection seam.

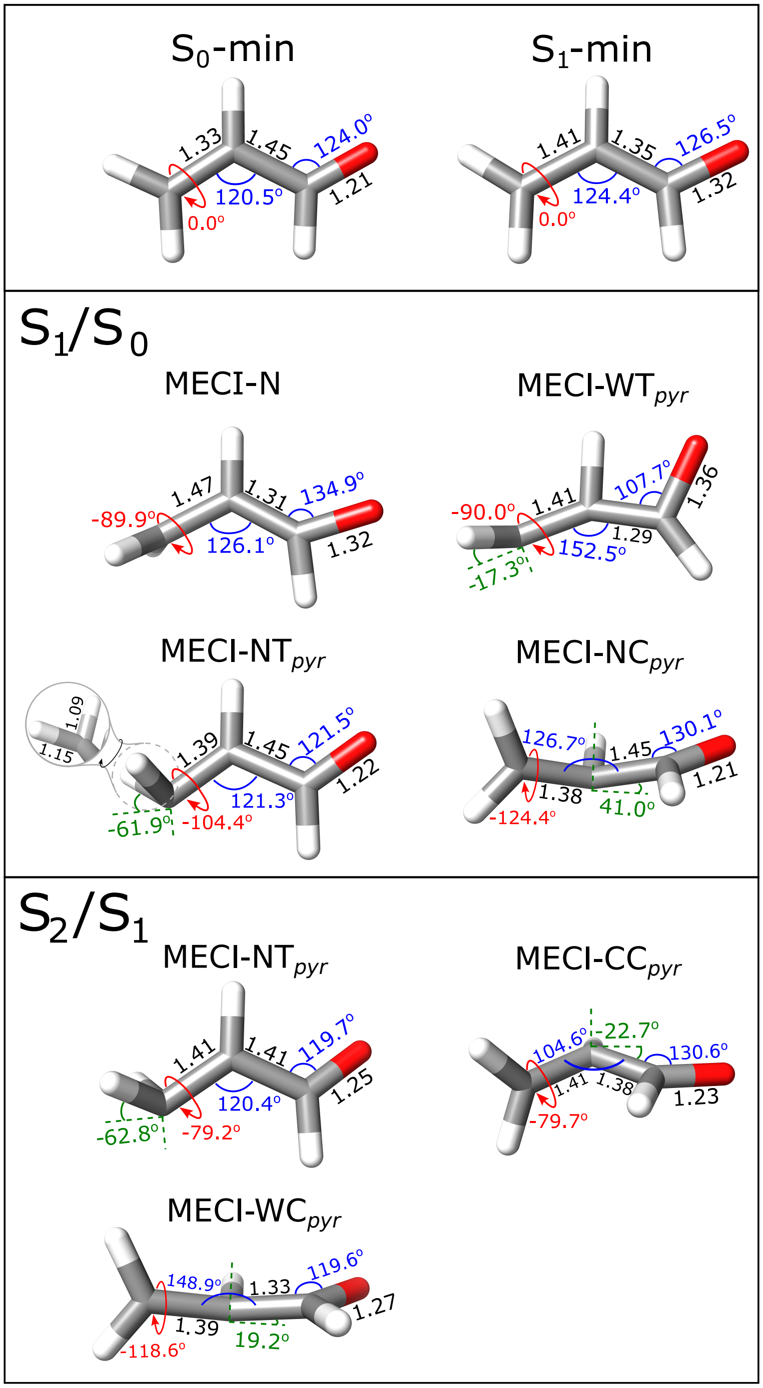

Figure S4: S_0- and S_1-minima and S_1/S_0- and S_2/S_1-MECI geometries for AC. The geometric labels and respective parameters are presented in Table S3. The indicated numbers represent: the bond lengths (Å) in black, angles in blue, torsion in red and pyramidalization in green.

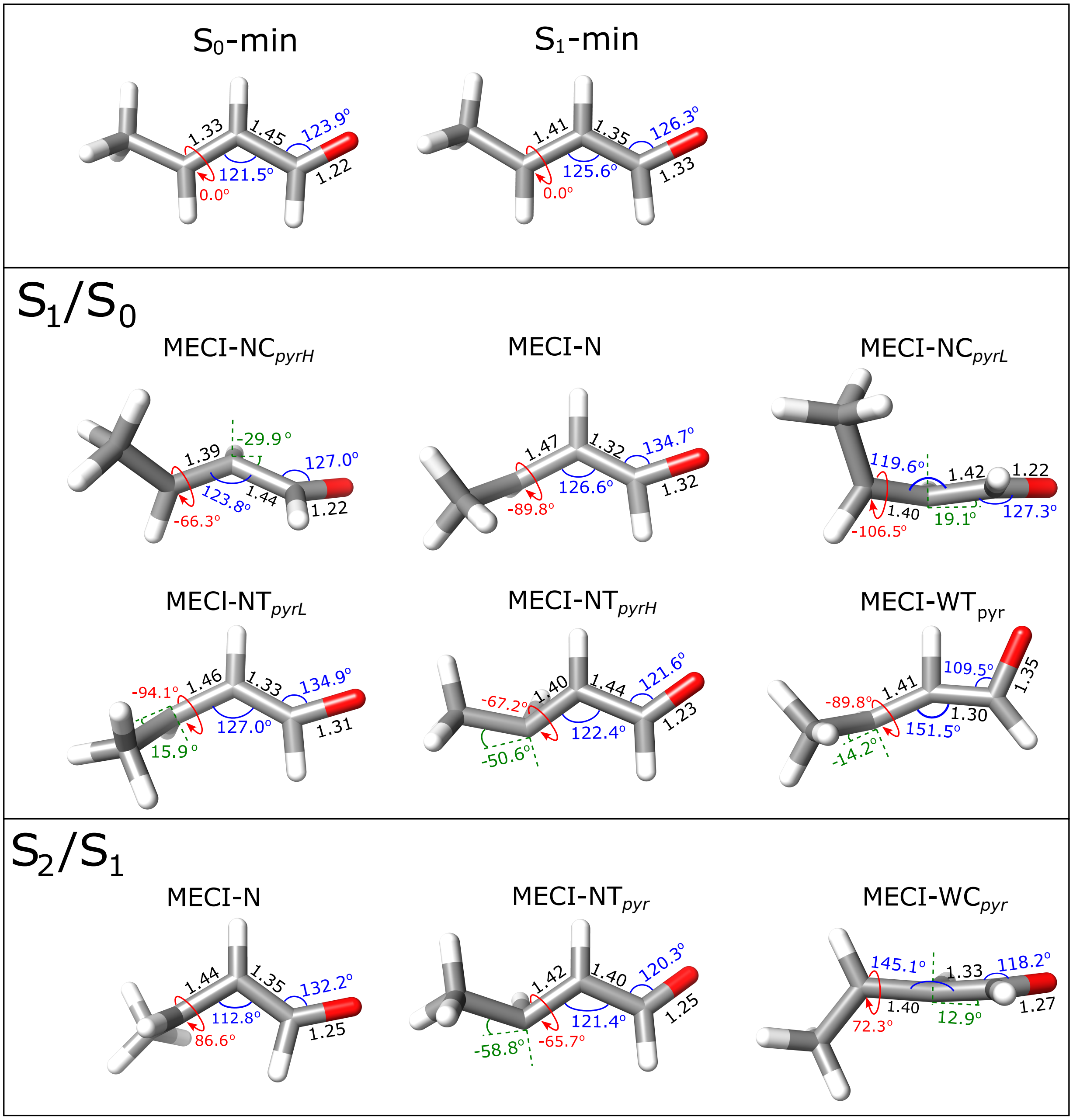

Figure S5: S_0- and S_1-minima and S_1/S_0- and S_2/S_1-MECI geometries for CR. The geometric labels and respective parameters are presented in Table S4. The indicated numbers represent: the bond lengths (Å) in black, angles in blue, torsion in red and pyramidalization in green.

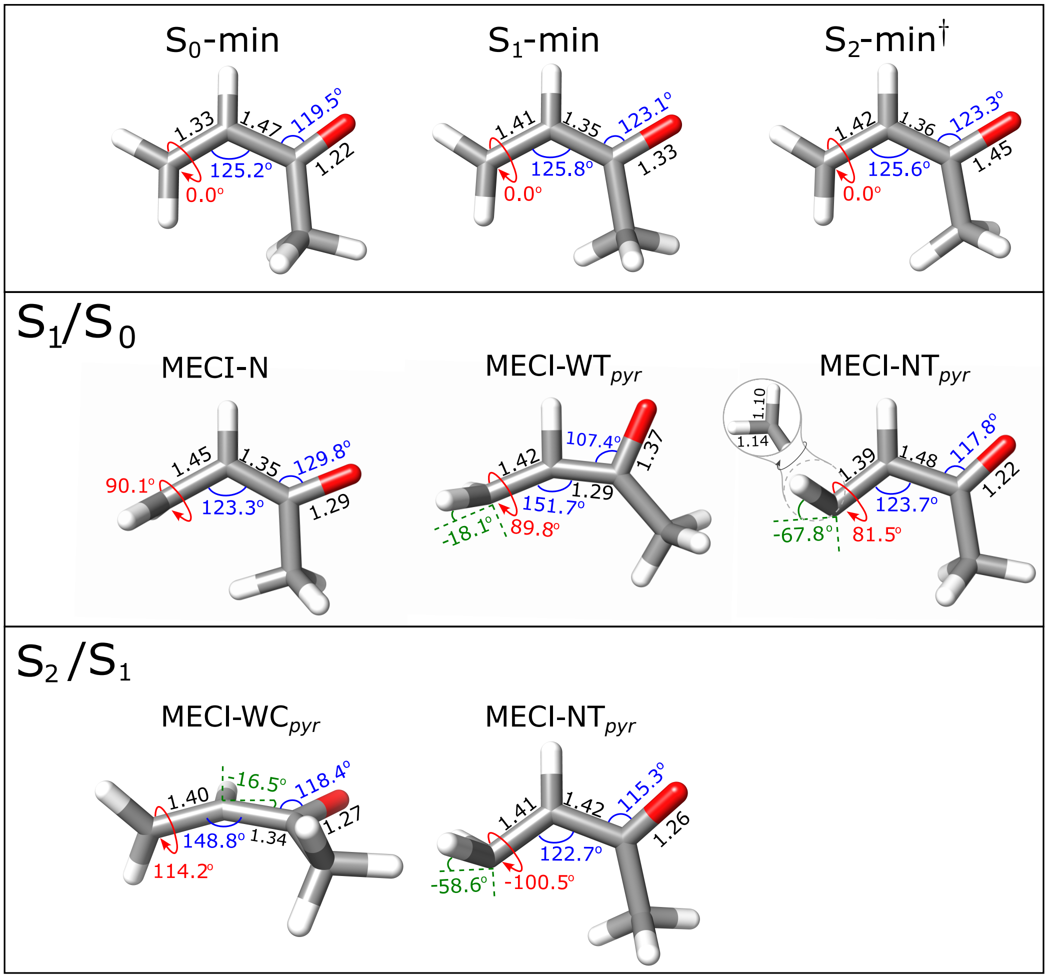

Figure S6: S_0- and S_1-minima and S_1/S_0- and S_2/S_1-MECI geometries for MVK. The geometric labels and respective parameters are presented in Table S5. The indicated numbers represent: the bond lengths (Å) in black, angles in blue, torsion in red and pyramidalization in green.

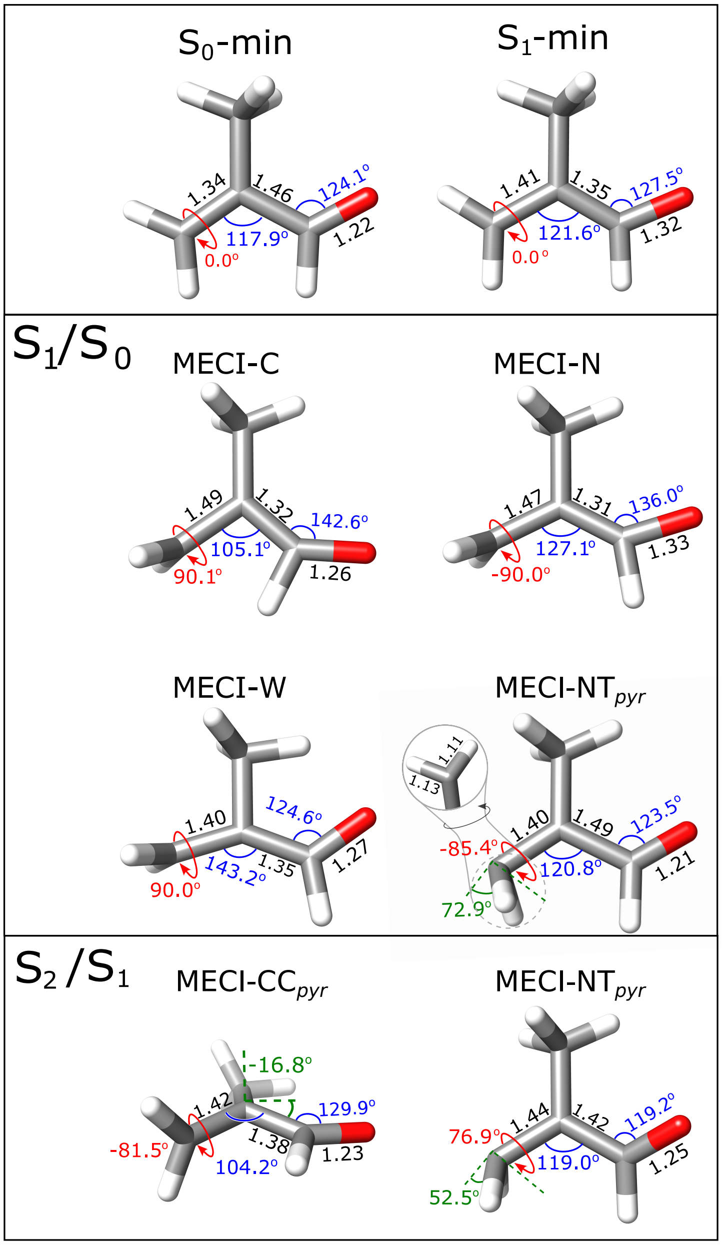

Figure S7: S_0- and S_1-minima and S_1/S_0- and S_2/S_1-MECI geometries for MA. The geometric labels and respective parameters are presented in Table S6. The indicated numbers represent: the bond lengths (Å) in black, angles in blue, torsion in red and pyramidalization in green.

S4 MECI nomenclature

Here, we outline the naming scheme used to label the identified MECIs. They are all characterized by the twist of the terminal methylene ($-CH_2$ or $-CHCH_3$ in CR). Besides BLA, the optimized MECIs differ mainly by the pyramidalization of the terminal and central C-atoms, and the angle extent at the central C-atom ($\angle C_1C_2C_3$). Definitions of geometric parameters are given in Figure S3. Thus, we adapt a nomenclature emphasizing those two aspects:

1. A MECI is labeled C, N, or W (contracted, neutral or wide) if the central $\angle C_1C_2C_3$ is $<110°$, within 110−130°, or $>130°$.

2. C_{pyr} or T_{pyr} is added to the name if the geometry displays substantial pyramidalization ($>10°$) at the central or terminal C-atom. Only the C-atom with the higher pyramidalization is considered in the name. However, it should be noted that in certain cases of central C-atom pyramidalization (especially, for wide angle structures), the terminal C-atom is also pyramidalized above 10°.

For example, a MECI with neutral central angle and terminal C-atom pyramidalization is named NT_{pyr}. When more than one MECI with the pyramidalization at the same C-atom exist on the same intersection seam, L or H is added as a subscript along with the pyramidalization to indicate a lower or higher degree of pyramidalization. According to our definition (Figure S3), the pyramidalization direction can have positive or negative sign. Such MECIs are energetically close (in the case of T_{pyr} and C_{pyr}) and have been grouped into the same type of MECI.

S5 Critical point analysis

In this section, we characterize and compare critical points on the potential energy surfaces (PESs) of the four linear α,β-carbonyls. The resulting structures, their energies and geometric parameters are provided in Figures S4 to S7, Tables S8 to S11 and Tables S3

to S6, respectively. Furthermore, Figure S9 provides a visual overview of the relative ground and valence excited-state energies. Compared to previous studies,[20–22] we have located several additional MECIs by using the non-adiabatic transitions as starting points for MECI searches. The naming scheme used to label the different MECIs is given in Section S4.

Minima. The S_0-minimum is planar (ignoring methyl H-atoms) with a BLA coordinate corresponding to the C=C−C=O resonance structure. Methylation red-shifts the excitation energy of the S_2 state and the *hh*-TDA level shows the same energy trend of the experimental absorption maxima[21,23] and XMS-CASPT2 results. At the S_0-minimum, the trend in S_2 excitation energy is MA<CR<MVK<AC. However, as mentioned in Section S1, the red-shifts predicted by *hh*-TDA are smaller than the experimental and XMS-CASPT2 counterparts. We have located a S_2 minimum for all the molecules, characterized by the twist of the methylene group and pyramidalization of the terminal C-atom. They are located significantly below the FC point (1.7, 1.3, 1.8, 1.6 eV for AC, CR, MVK, MA respectively) with S_2–S_1 energy gaps $<$0.04 eV. Thus, such minima are not really acting to trap population, but rather as avoided crossing points between S_2 and S_1 states. In addition, for MVK, we have also found a shallow planar minimum on S_2 (S_2-min*) located 0.7 eV below the FC point, but only at the *hh*-TDA level. The barrier out of this minimum is $<$0.05 eV, making it insignificant in the S_2 deactivation (see discussion in the main text). Structurally, the S_1-minimum (S_1-min) is also planar but with an almost inverted BLA relative to the FC point. The energies at the S_1-min are very similar to the experimental[24] and XMS-CASPT2 values with methylation inducing blue-shifts. However, the trend in blue-shifts of the energies of S_1-min with respect to methylation is different in *hh*-TDA level relative to the experimental (and XMS-CASPT2) values. Another local S_1 minimum (S_1-min(180°) indicating a rotation of the formyl group) is located ∼0.2–0.3 eV above the aforementioned S_1-min. This indicates another possible pathway for the S_1 deactivation accessed provided 180° formyl rotation occurs. However, this rarely happened in our simulations. It should

also be noted that for MVK, the optimized minima on the different electronic states have varying orientations of the methyl group.

S_2/S_1-MECIs. The S_2/S_1-MECIs are all characterized by ∼90° torsion and located below the FC point. The lowest-energy S_2/S_1-MECIs are characterized by central pyramidalization and contracted/wide central angles in all but CR, where there is almost no pyramidalization. Starting from the non-adiabatic transitions, we further located higher-energy S_2/S_1-MECIs with high PyrT and neutral central angle (NT_{pyr}). All S_2/S_1-MECIs display an expanded BLA relative to the FC point and feature charge-transfer across the twisted C=C bond with the pyramidalized C-atom acquiring negative charge (Figures S10 and S11). This charge-transfer behavior induced by twist-pyramidalization (termed "sudden polarization") is a common feature in unsaturated hydrocarbons such as ethylene[25–27] and butadiene.[28–30] Even the lowest energy S_2/S_1-MECI-N for CR, featuring only slight pyramidalization, display such charge-transfer behavior (Figure S13).

S_1/S_0-MECIs. For AC/MVK/MA, the lowest-energy region on the seam is S_1/S_0-MECI-N. For CR, several low energy S_1/S_0-MECIs are isoenergetic, including MECI-N, suggesting that the PES at that region of the seam space is rather flat. The S_1/S_0-MECI-N for all the systems is characterized by a sloped topography with predominant tilt along the gradient-difference vector.(Table S12) In addition, the S_1/S_0-MECI-N is located at ∼0.7 eV above the S_1-min in AC/CR/MA, while only at ∼0.4 eV in MVK. This stabilizing effect of formyl methylation is likely a consequence of the weakly electron-donating methyl group that stabilizes the partial positive charge on the carbonyl C-atom. While this is expected to have significant impact on internal conversion following photoexcitation to S_1, such effects are less pronounced in our case where the kinetic energy available upon reaching S_1 from S_2 is high (further discussion in Section 3 of the main text). This S_1/S_0-MECI-N is characterized by BLA expansion and an alter-

ation of the electronic character relative to the S_2/S_1-MECIs. It features biradicaloid character[31] with an electron residing at each of the two non-degenerate n_O and terminal C p orbitals. No significant charge separation occurs along the pathway from S_1-min to S_1/S_0-MECI-N (Figure S12A). For comparison, we note that although covalent in nature, this character does not resemble the MECI mediating S_1/S_0 decay via the covalent pathway in butadiene. In butadiene, the MECI corresponding to covalent pathway displays a quasi-tetraradical[20,29,30,32] behavior as a consequence of the intersection between the doubly-excited S_1 state with the ground-state. There are additional higher-energy S_1/S_0-MECIs. We have located a terminal twist-pyramidalized analog of the S_2/S_1-MECI-NT_{pyr} (NT_{pyrH} for CR). These two MECIs have the same electronic character, i.e. ionic character across both seams, illustrated by the same charge-separated character on the S_1 state between MECI-NT_{pyr} of both S_2/S_1-and S_1/S_0-intersections (Figure S10B). However, such charge-transfer character could be oppositely directed depending on the direction of approach along the MECI gradient. The main difference at the S_1/S_0-MECI-NT_{pyr} is a contraction of BLA relative to the analog at the S_2/S_1 seam. However, at the S_1/S_0 seam, pyramidalization becomes asymmetric and one of the C-H bonds of the methylene group is almost in the same plane of the main chromophore. This leads to different bond distances for the two C-H bonds of the methylene group, as also observed in ethylene[26] and butadiene.[28–30] In CR, the S_2/S_1- and S_1/S_0-MECI-NT_{pyr} are located at higher energies relative to the other molecules. Since the terminally pyramidalized C-atom takes on carbanion character, the weakly electron-donating terminal methyl group in CR destabilizes this type of MECI. Both AC and CR also feature lower-energy MECIs characterized by 180°-twisted formyl group. Although earlier studies on S_1 internal conversion and intersystem-crossing dynamics in AC reported S_1/S_0 non-adiabatic transitions featuring such geometries,[33,34] they are extremely rare in our simulations. In addition, other higher-energy MECIs characterized by contracted, neutral and wide angles, with or

without pyramidalization also exist depending on the molecule.

S6 Adiabatic population fitting

The S_2 population profiles of the systems were fitted to a delayed mono-exponential decay

$$p_2(t) = \begin{cases} 1 & t < t_0 \\ ce^{-(t-t_0)/\tau_1} + (1-c) & t \geq t_0 \end{cases} \tag{S1}$$

and the S_0 populations to a delayed bi-exponential growth

$$p_0(t) = \begin{cases} 0 & t < t_0 \\ 1 - \left(ce^{-(t-t_0)/\tau_1} + (1-c)e^{-(t-t_0)/\tau_2}\right) & t \geq t_0 \end{cases} \tag{S2}$$

Here, t_0 is the lagtime indicating the delay in population decay/growth, while $\tau_{1/2}$ are the decay/growth lifetimes. To compensate for any residual population remaining on S_2 due to the finite population threshold deciding whether a TBF is allowed to spawn (population >0.01), a constant term was added to the delayed mono-exponential function. Similar adjustment was done for the S_0 population fit. We refrained from fitting the S_1 population profiles given that it would require a more complex, non-sequential model (and different lagtimes) to account for both ballistic and IVR-limited regimes. The fitted time constants are summarized in Table S7.

Table S7: Fitted lagtimes and decay constants (in fs) for the adiabatic populations of acrolein and methylated derivatives together with their associated uncertainties and amplitudes (parenthesis). Uncertainties were obtained from 1500 bootstrapped fitted samples. Decay onsets correspond to the time of initial population transfer (bootstrapped) and therefore differ from the fitted τ_0 lagtimes.

system	state	decay onset	t_0	τ_1	τ_2
	S_2	27 ± 8	44 ± 5	(0.98) 53 ± 7	
AC	S_0	39 ± 9	40 ± 7	(0.70) 88 ± 18	(0.30) 859 ± 231
	S_2	37 ± 2	41 ± 4	(0.98) 50 ± 4	
CR	S_0	43 ± 2	35 ± 6	(0.73) 97 ± 18	(0.27) 800 ± 232
	S_2	21 ± 3	24 ± 4	(0.98) 63 ± 10	
MVK	S_0	37 ± 3	26 ± 6	(0.65) 93 ± 26	(0.35) 743 ± 233
	S_2	25 ± 3	34 ± 5	(0.98) 50 ± 5	
MA	S_0	43 ± 3	27 ± 9	(0.61) 93 ± 25	(0.39) 2531 ± 422

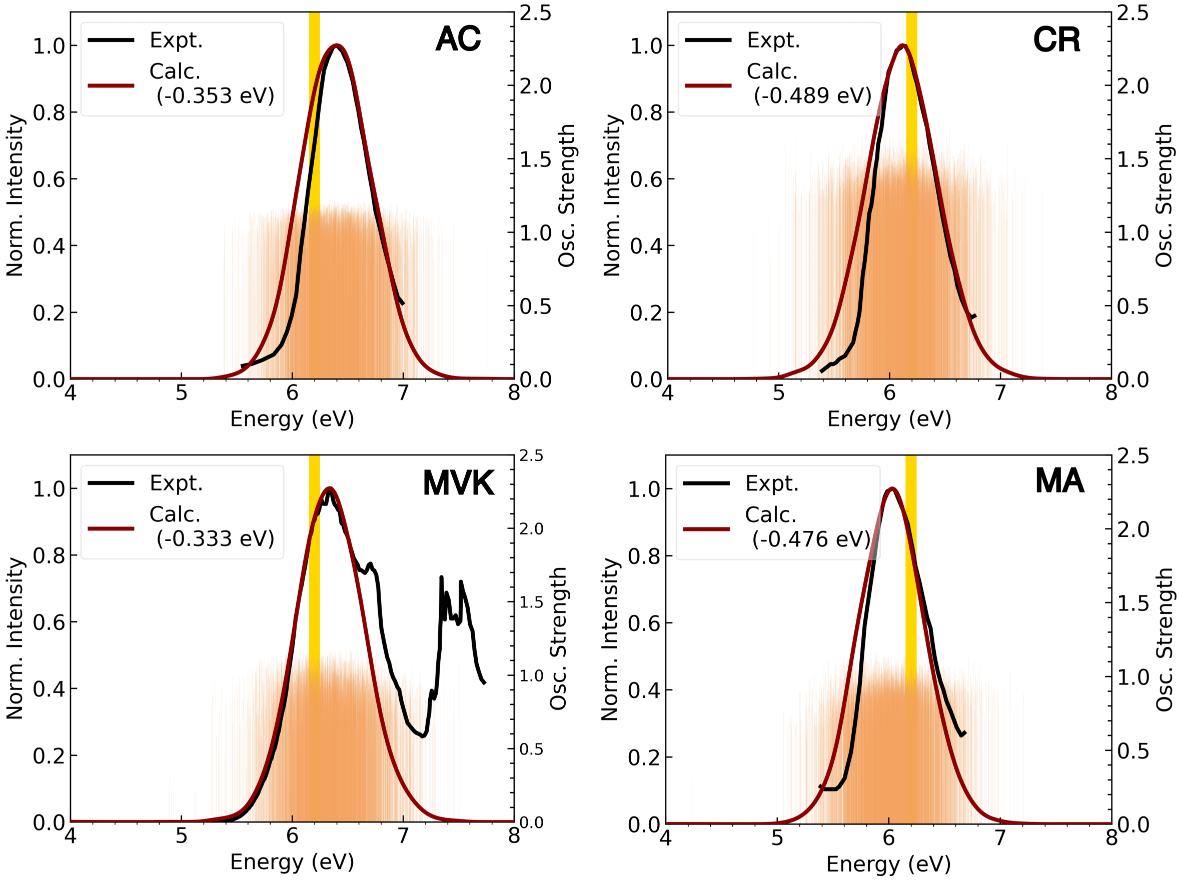

Figure S8: Comparison of simulated and experimental electronic absorption spectra computed based on 5000 configurations sampled from a finite-temperature (300 K) ground-state harmonic Wigner distribution computed at the *hh*-TDA-ωPBEh/6-31G(d,p) level of theory. Individual sticks were convolved with a Gaussian envelope (FWHM=0.2 eV) and shifted to align with the experimental absorption maxima. The yellow area marks the energetic window (pump photon energy 6.200±0.025 eV, corresponding to 200 nm) within which the initial conditions for the AIMS dynamics were selected. Stick spectra show contributions from $S_2(\pi\pi^*)$. Weak contributions from $S_1(n\pi^*)$ are outside the plot range (<4 eV). Experimental spectra were digitized from Refs. 21,23.

Table S8: Energies for critical points (eV) of AC obtained at the *hh*-TDA/6-31G(d,p) level of theory. Energies are reported relative to the ground-state energy at the S_0-min point.

Type	S_0	S_1	S_2
S_0-min	0.000	3.850	6.913
S_1-min	0.696	3.232	6.473
S_1-min(180°)	0.922	3.504	6.378
S_2-min(tw-py)	4.101	5.170	5.198
S_1/S_0-MECIs‡			
N	3.908	3.908	5.718
WT_{pyr}(180°)	4.125	4.125	5.582
NC_{pyr}(180°)	4.236	4.236	5.734
NC_{pyr}	4.339	4.339	5.815
WT_{pyr}	4.668	4.668	6.746
NT_{pyr}	4.923	4.923	6.438
S_2/S_1-MECIs‡			
CC_{pyr}	4.029	4.792	4.792
WC_{pyr}	4.254	4.967	4.967
NT_{pyr}	4.152	5.276	5.276

‡Not been confirmed to be minima on the intersection seam.

Table S9: Energies for critical points (eV) of CR obtained at the *hh*-TDA/6-31G(d,p) level of theory. Energies are reported relative to the ground-state energy at the S_0-min point.

Type	S_0	S_1	S_2
S_0-min	0.000	3.966	6.813
S_1-min	0.723	3.311	6.341
S_1-min(180°)	0.917	3.564	6.258
S_2-min(tw-py)	4.411	5.530	5.561
S_1/S_0-MECIs‡			
NT_{pyr}(180°)	3.762	3.762	5.109
N(180)	3.892	3.892	4.912
NC_{pyrL}	3.921	3.921	5.328
NT_{pyrL}	3.942	3.942	5.269
N	3.986	3.986	5.226
NC_{pyrH}	4.062	4.062	5.349
WT_{pyr}	4.668	4.668	6.253
NT_{pyrH}	5.174	5.174	6.800
S_2/S_1-MECIs‡			
N	3.852	4.385	4.385
WC_{pyr}	4.100	4.752	4.752
NT_{pyr}	4.450	5.612	5.612

‡Not confirmed to be minima on the intersection seam.

Table S10: Energies for critical points (eV) of MVK obtained at the *hh*-TDA/6-31G(d,p) level of theory. Energies are reported relative to the ground-state energy at the S_0-min point.

Type	S_0	S_1	S_2
S_0-min	0.000	3.978	6.873
S_1-min	0.790	3.315	6.408
S_1-min(180°)	0.954	3.685	6.306
S_2-min(tw-py)	3.968	5.053	5.088
S_2-min*	1.322	3.978	6.137
S_1/S_0-MECIs‡			
N	3.715	3.715	5.624
WT_{pyr}	4.592	4.592	6.640
NT_{pyr}	4.794	4.794	6.313
S_2/S_1-MECIs‡			
WC_{pyr}	4.116	4.896	4.896
NT_{pyr}	4.075	5.192	5.192

‡Not confirmed to be minima on the intersection seam.

Table S11: Energies for critical points (eV) of MA obtained at the *hh*-TDA/6-31G(d,p) level of theory. Energies are reported relative to the ground-state energy at the S_0-min point.

Type	S_0	S_1	S_2
S_0-min	0.000	4.037	6.720
S_1-min	0.749	3.385	6.507
S_1-min(180°)	0.971	3.694	6.353
S_2-min(tw-py)	3.830	5.054	5.086
S_1/S_0-MECIs‡			
N	4.125	4.125	6.102
W	4.154	4.154	6.033
C	4.288	4.288	5.930
NT_{pyr}	4.741	4.741	6.400
S_2/S_1-MECIs‡			
CC_{pyr}	3.984	4.955	4.955
NT_{pyr}	3.955	5.187	5.187

‡Not confirmed to be minima on the intersection seam.

Table S12: Conical intersection parameters for S_1/S_0-MECI-N across the systems. Parameters are defined in Ref. 35.

molecule	ΔE (eV)	δ_{gh} (a.u.)	Δ_{gh} (a.u.)	σ	θ (°)	s_x	s_y
AC	0.676	0.0320	0.9365	2.9837	3.6755	2.9776	0.1913
CR	0.675	0.0336	0.8456	2.6290	0.3907	-2.6290	-0.0179
MVK	0.400	0.0364	0.2604	1.4186	1.9824	1.4178	0.0491
MA	0.740	0.0382	0.9013	3.3295	0.0798	-3.3295	-0.0046

ΔE: energy with respect to the S_1 energy at S_1-min; δ_{gh}: pitch; Δ_{gh}: asymmetry; σ, θ: relative tilt and tilt direction; s_x and s_y: projections of the gradient sum vector onto the branching plane along the rotated g and h vectors.

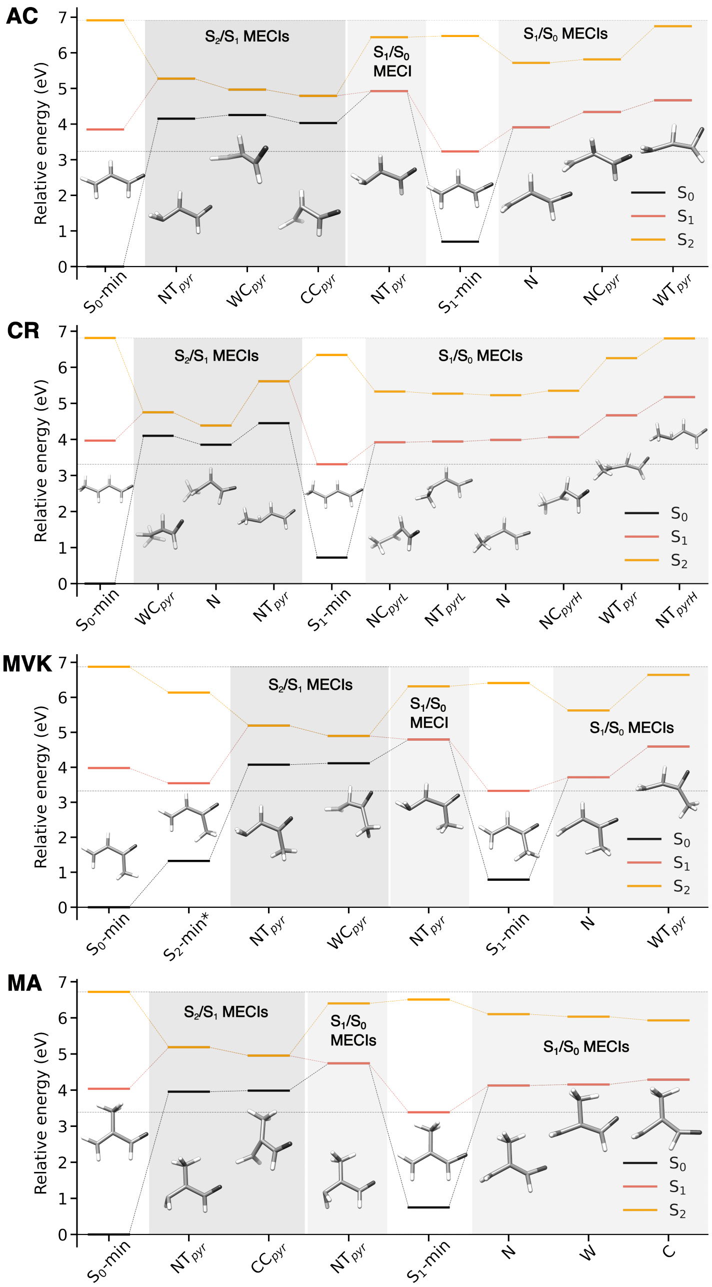

Figure S9: Relative energy of the critical points of AC, CR, MVK and MA, at the *hh*-TDA/ω-PBEh/6-31G(d,p) level relative to their respective S_0-minimum. The S_2/S_1-and the S_1/S_0-MECIs are separated by different shades of gray boxes. The S_0- and S_1- minima are both planar and differ by their BLA. It should be noted that the planar S_2-minimum (S_2-min*) located for MVK (differs by BLA compared to the S_0 and S_1 minima) is a local minima on the PES (not a minimum at the XMS-CASPT2 level). In addition, there are several near-degenerate MECIs depending on the pyramidalization direction. These are been grouped together and only the lowest energy variants are shown. MECIs of each type are confirmed to be within 0.2 eV of the lowest energy MECI of the same type. The accessibility of MECI-NT$_{pyr}$ in the dynamics of CR is negligible and hence these MECIs are located as the final point in their respective intersection seam for CR.

S7 Additional Analyses

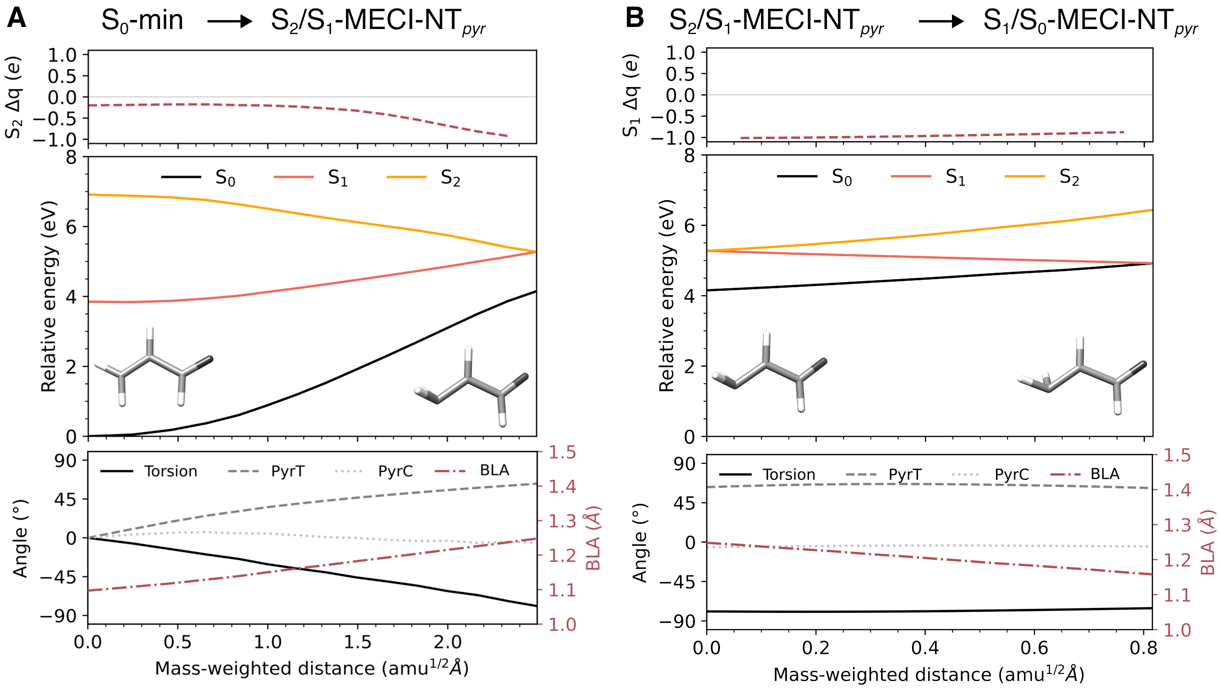

Figure S10: Evolution of difference Mulliken charges on the terminal and central C-atoms (upper panel), electronic state energies (middle panel), and key geometric parameters (lower panel) in AC along geodesically interpolated pathway from (A) S_0-min to S_2/S_1-MECI-NT$_{pyr}$, and from (B) S_2/S_1-MECI-NT$_{pyr}$ to S_1/S_0-MECI-NT$_{pyr}$. As PyrT increases from the FC region toward S_2/S_1-MECI-NT$_{pyr}$, the charge difference on S_2 (S_2 Δq (e)) also increases to −1.0. This implies charge transfer from the central to the terminal C-atom. Between S_2/S_1-MECI-NT$_{pyr}$ and S_1/S_0-MECI-NT$_{pyr}$, BLA contracts, whereas, torsion, PyrT (becomes asymmetric) and PyrC do not change significantly. The charge-transfer character is, thus, maintained on such a pathway as has been illustrated by tracking the charge difference on S_1 (S_1 Δq (e)). In other words, the electronic character is preserved in going from S_2/S_1-MECI-NT$_{pyr}$ to S_1/S_0-MECI-NT$_{pyr}$, characterizing the ballistic pathway. The energies have been calculated relative to the S_0 energy at the S_0-min. Charge differences at the MECIs have been excluded because of the mixed character at electronic degeneracies.

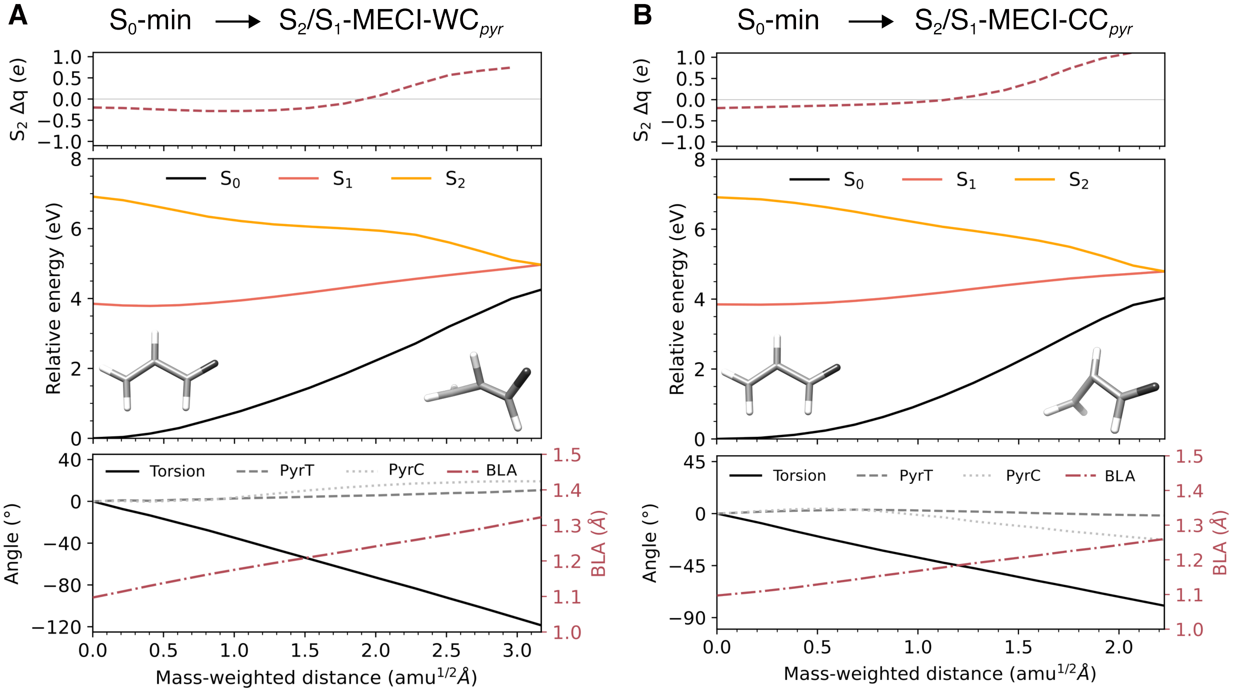

Figure S11: Evolution of difference Mulliken charges on the terminal and central C-atoms (upper panel), electronic state energies (middle panel), and key geometric parameters (lower panel) in AC along geodesically interpolated pathway from S_0-min (A) to the S_2/S_1-MECI-WC_{pyr}, and (B) to the S_2/S_1-MECI-CC_{pyr}. As PyrC increases along both of these paths, the charge difference on S_2 (S_2 Δq (e)) also increases toward unity, indicating charge transfer from the terminal to the central C-atom. The energies have been calculated relative to the S_0 energy at the S_0-min. Charge differences at the MECIs have been excluded because of the mixed character at electronic degeneracies.

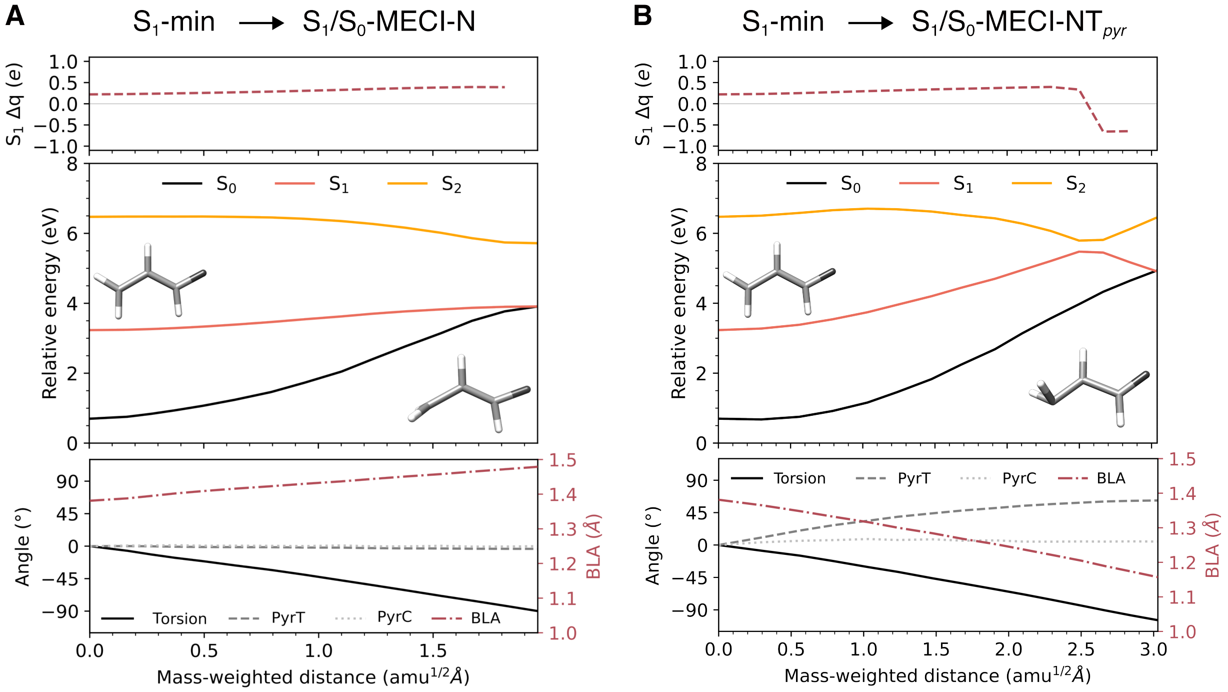

Figure S12: Evolution of difference Mulliken charges on the terminal and central C-atoms (upper panel), electronic state energies (middle panel), and key geometric parameters (lower panel) in AC along geodesically interpolated pathway from S_1-min (A) to the S_1/S_0-MECI-N, and (B) to the S_1/S_0-MECI-NT$_{pyr}$. From S_1-min to S_1/S_0-MECI-N, the torsion increases from 0 to ∼-90° and BLA increases. However, pyramidalization does not change leading to no significant charge-transfer in reaching S_1/S_0-MECI-N. On the other hand, an increase in PyrT is required to reach S_1/S_0-MECI-NT$_{pyr}$ from S_1-min. This leads to a barrier corresponding to an avoided crossing with S_2/S_1-MECI-NT$_{pyr}$. This brings the charge difference close to -1.0 near the S_1/S_0-MECI-NT$_{pyr}$, similarly to Figure S10. The energies have been calculated relative to the S_0 energy at the S_0-min. Charge differences at the MECIs have been excluded because of the mixed character at electronic degeneracies.

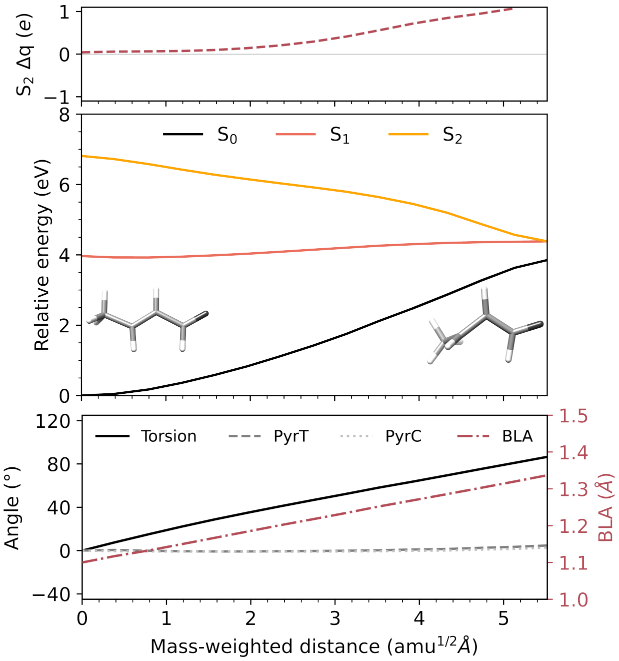

Figure S13: Evolution of difference Mulliken charges on the terminal and central C-atoms (upper panel), electronic state energies (middle panel), and key geometric parameters (lower panel) in CR along geodesically interpolated pathway from S_0-min to the S_2/S_1-MECI-N. Even though PyrC and PyrT minimally increases along both of these paths, the charge difference on S_2 (S_2 Δq (e)) increases toward unity along the pathway. Methyl group at the terminal carbon stabilizes the increase of partial positive charge on terminal carbon, while the central carbon also gains partial negative charge (charge-transfer from terminal to central C-atom). The energies have been calculated relative to the S_0 energy at the S_0-min. Charge differences at the MECIs have been excluded because of the mixed character at electronic degeneracies.

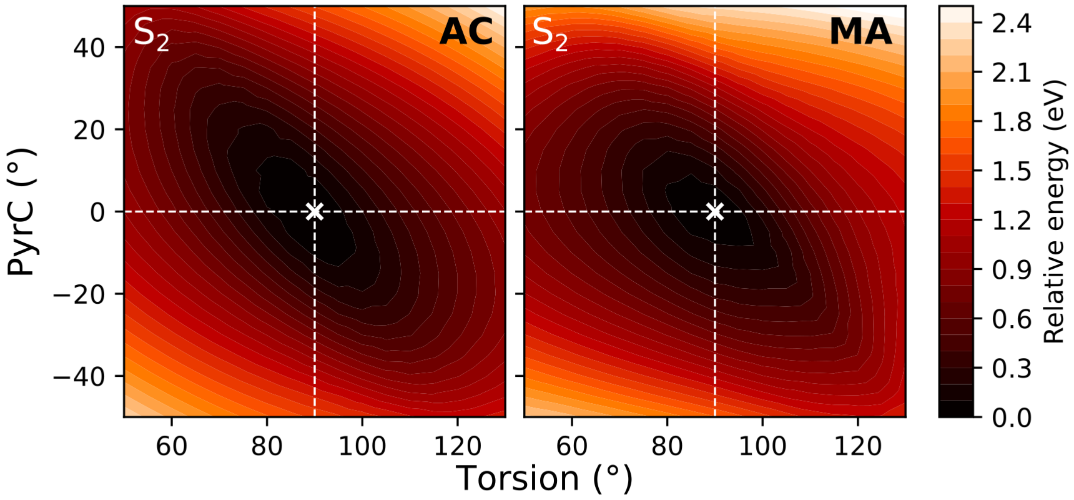

Figure S14: Contour plots of the S_2 PES around S_2/S_1-MECI-NT$_{pyr}$ (white cross) in AC and MA along pyrC and torsional modes (energies relative to the minimum for each system). Both displacements were generated by rigid displacements along the respective coordinates starting from S_2/S_1-MECI-NT$_{pyr}$. The minimum in the PES cut is tighter in MA compared to AC.

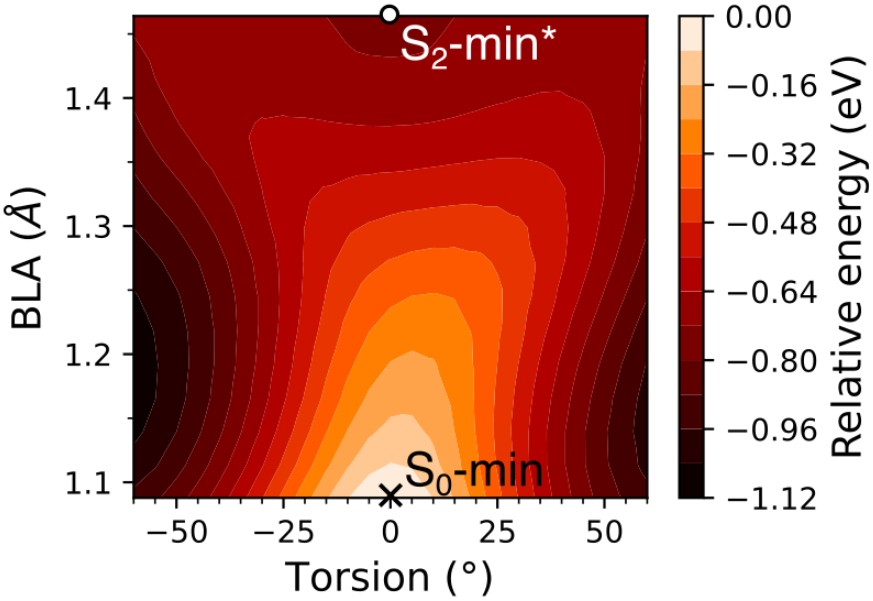

Figure S15: Contour plots of the S_2 PES in MVK along the BLA and torsional modes (energies relative to S_2 energy at the S_0-min). The BLA axis was obtained by geodesic interpolation[36] between the S_0-min and S_2-min*, while the torsional displacement was generated by rigid rotation. This means that barriers are upper bounds to the true barriers. The asymmetry is caused by a slight pyramidalization at the carbonyl C-atom along the interpolated path.

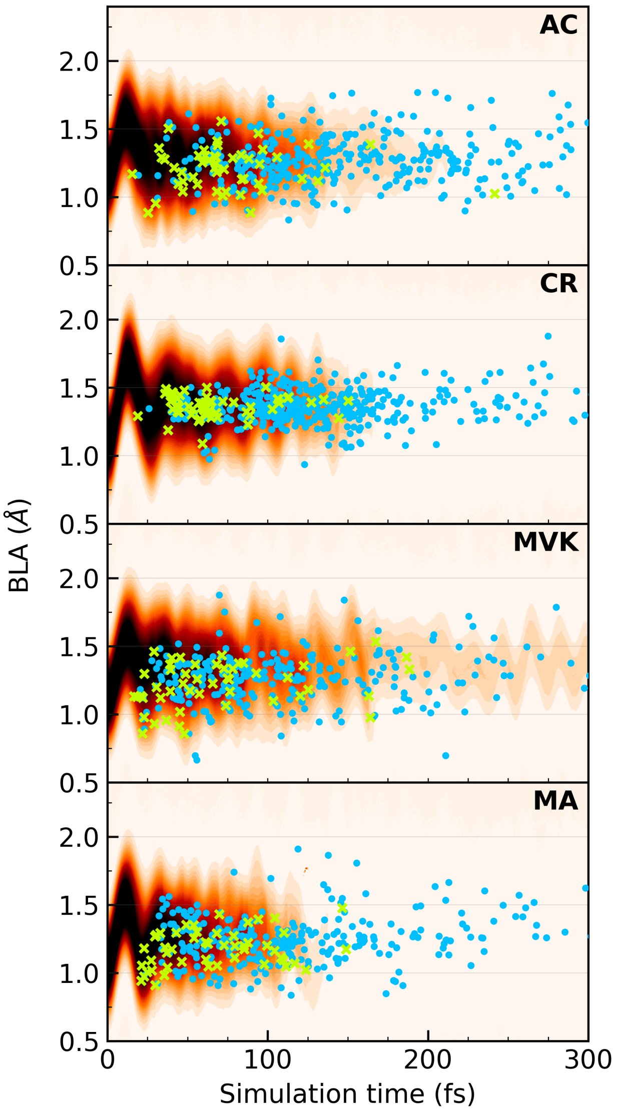

Figure S16: Time evolution of the S_2 wavepacket density along the BLA mode within the first 300 fs following photoexcitation. The green crosses indicate the first non-adiabatic transfer event for each IC while the blue filled circles indicate subsequent transfer events. All systems undergoes BLA-expansion-contraction oscillation before they reach the intersection seam.

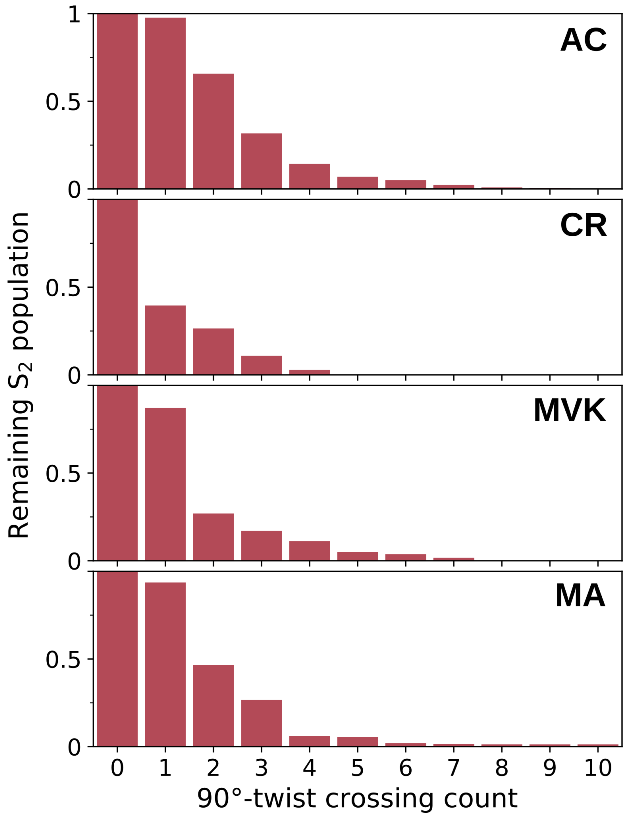

Figure S17: Population associated with the parent TBFs on S_2 following one or more crossings of methylene 90°-twisted configurations. In CR, the first approach to the intersection seam is much more efficient at mediating population transfer relative to the other molecules.

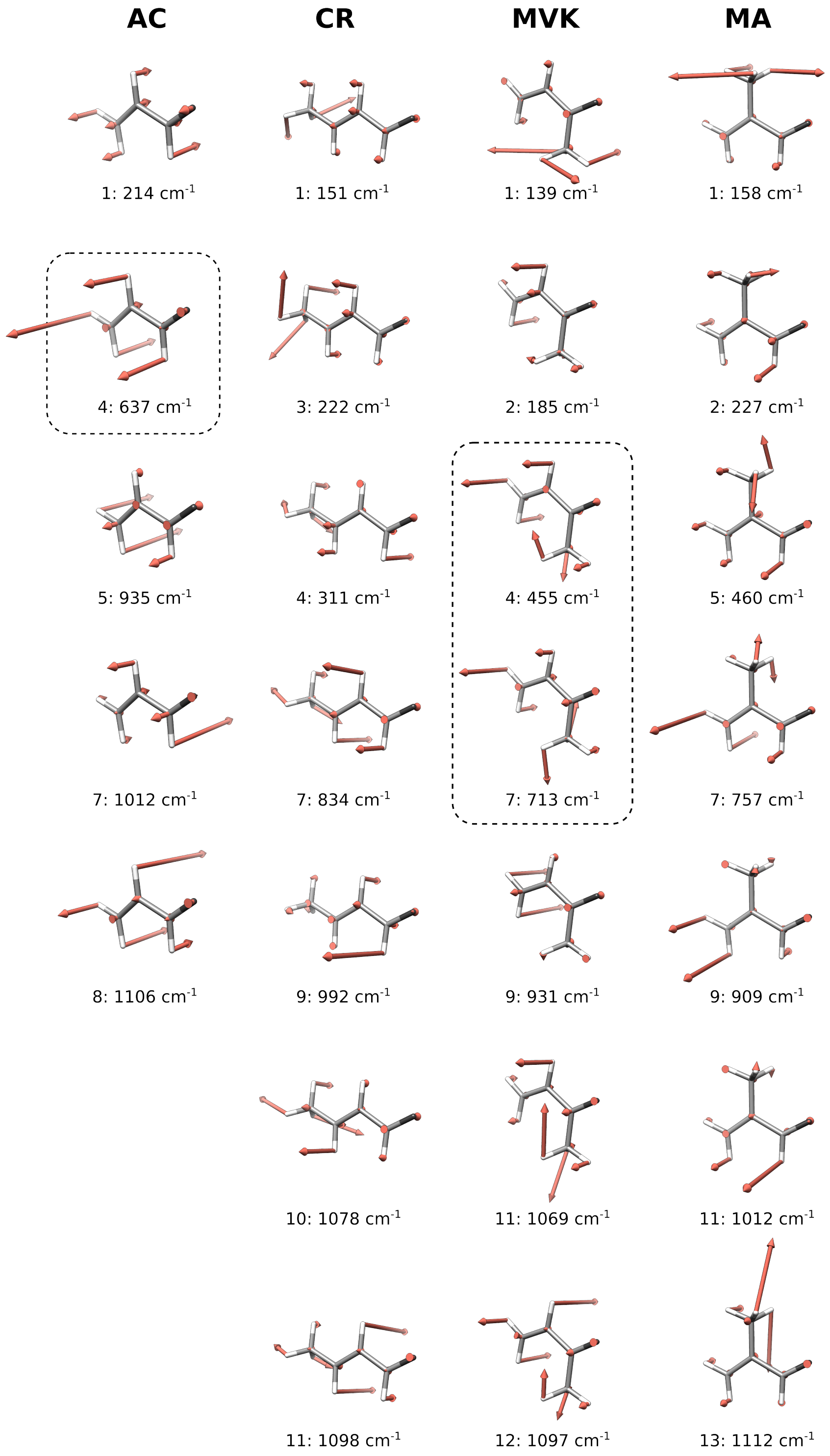

Figure S18: Normal modes dominated by out-of-plane motion and with frequencies <1500 cm^{-1}, obtained with *hh*-TDA. The boxed normal modes correspond to in-phase PyrT and PyrC modes. MVK possesses two such compared to one in AC.

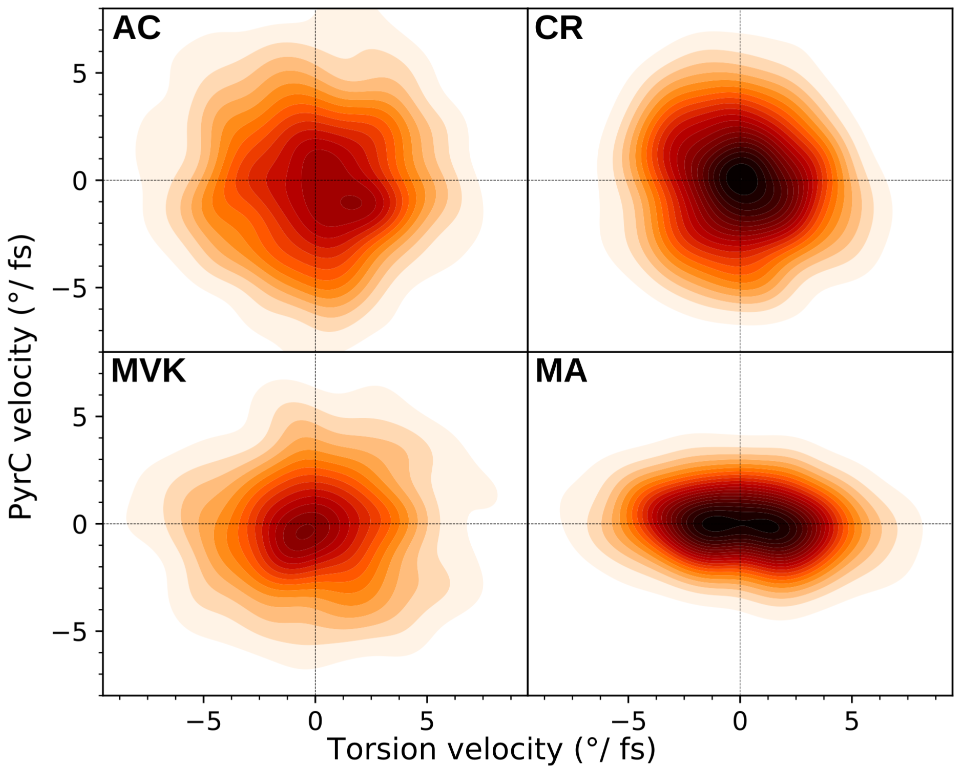

Figure S19: Distribution of velocity components along the PyrC and torsional modes immediately following the S_2/S_1 transition events. Velocities were computed as averages over a 5-fs period following transfer to S_1. Each non-adiabatic transition event (not weighted by their population) was convolved with a two-dimensional Gaussian with standard deviation of 1°/fs along each direction.

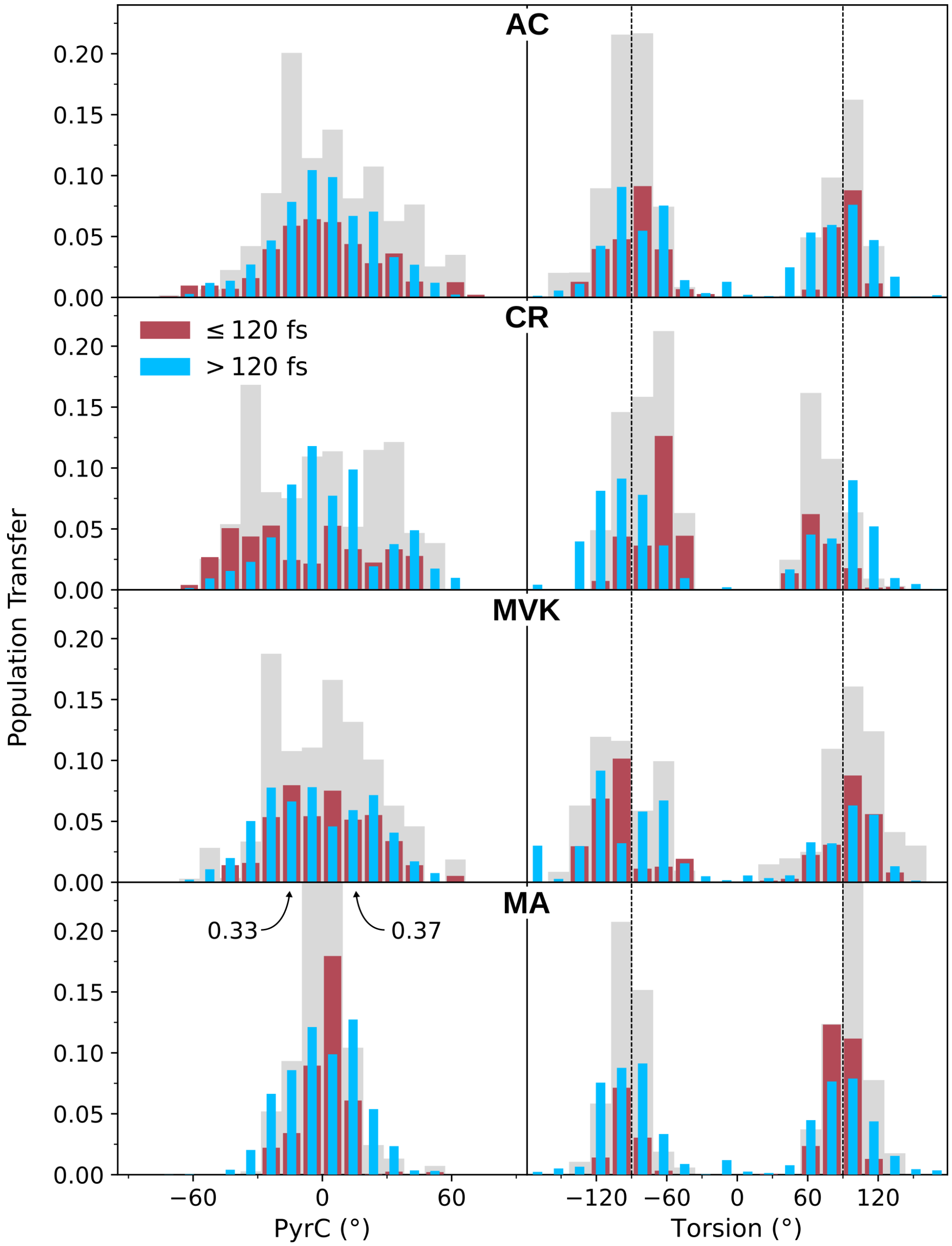

Figure S20: Population-weighted distributions of PyrC and torsional (wrapped) modes for the S_2/S_1 non-adiabatic transitions (gray) as well as the S_1/S_0 counterparts divided into early (red, ≤120 fs) and late (blue, >120 fs) events.

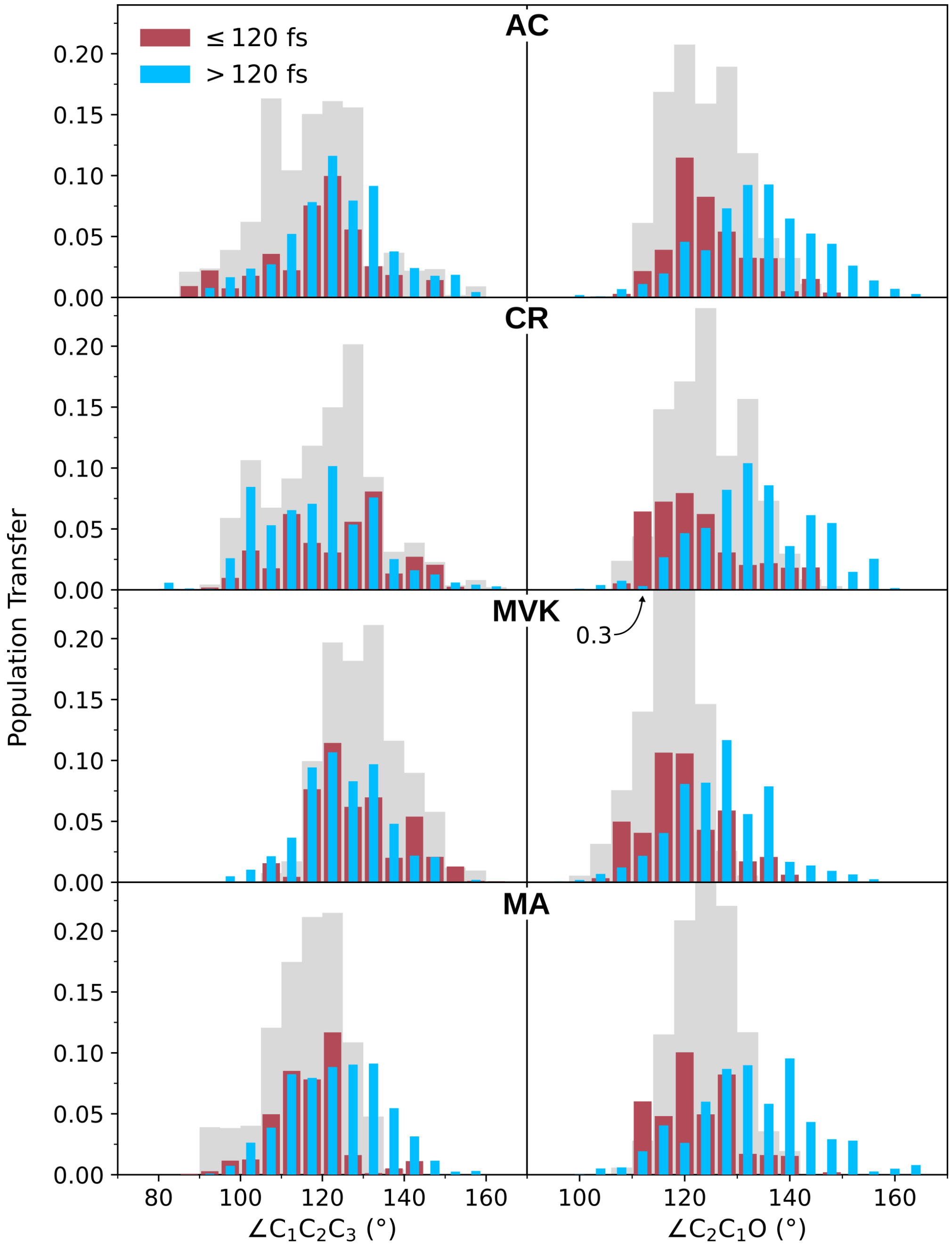

Figure S21: Population-weighted distributions of $\angle C_1C_2C_3$ and $\angle C_2C_1O$ for S_1/S_0 non-adiabatic transitions divided into early (red, $\leq$120 fs) and late (blue, $>$120 fs) events.

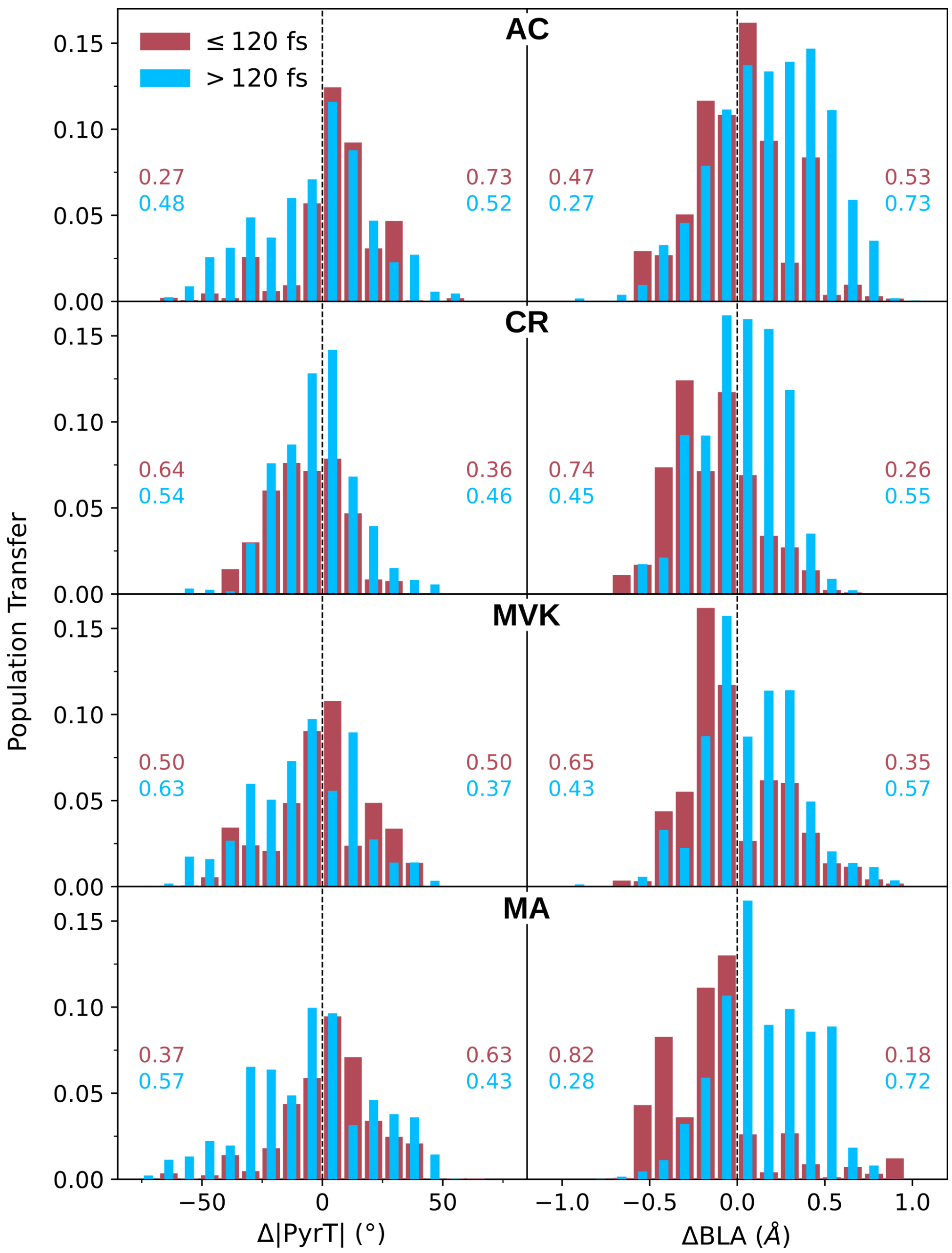

Figure S22: Population-weighted Δ|PyrT| (left) and ΔBLA (right) distributions for the S_1/S_0 non-adiabatic transitions divided into early (red, ≤120 fs) and late (blue, >120 fs) events. Δ|PyrT| has been defined as the difference between the absolute value of PyrT of a S_1/S_0 transfer event and that of its parent S_2/S_1 transfer event (similarly for ΔBLA but without the absolute value). The vertical dashed $X = 0$ lines indicate contraction/expansion relative to the parent S_2/S_1 geometry. The inset values indicate the fraction on either side. While reaching S_1/S_0-MECI-NT$_{pyr}$ from the S_2/S_1-counterpart requires additional displacement along PyrT ∼20°/9° in MA/MVK (Tables S5 and S6), we do not see substantial PyrT increase in these two systems.

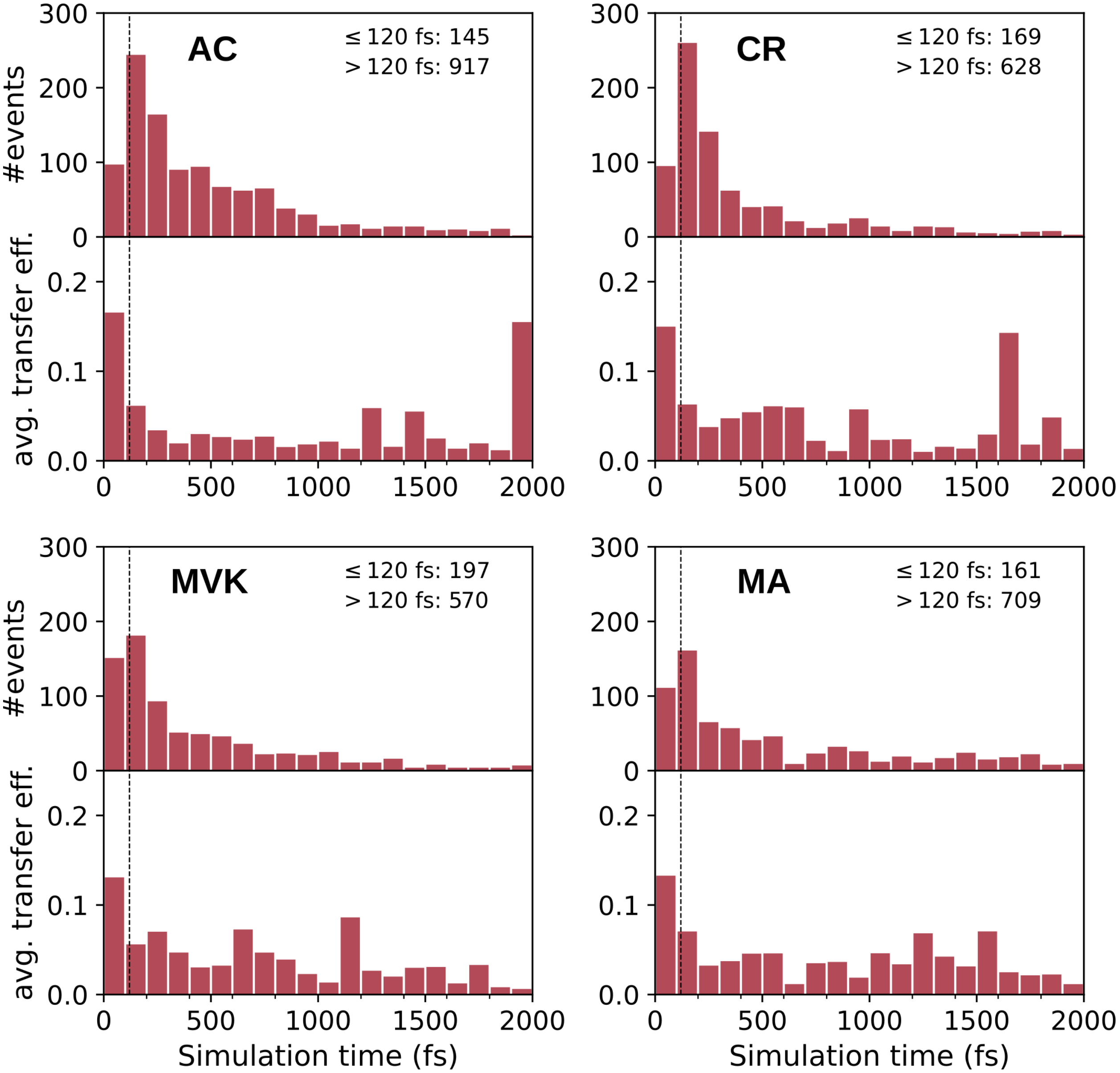

Figure S23: (top panels) Number of non-adiabatic transition events over time. Dashed vertical lines indicate the 120-fs threshold used to distinguish early and late S_1 decay. Accumulated counts for early and late decay are given in the legend. For AC, the number of non-adiabatic transfer events at late times (>120 fs) is about twice that of the other systems; (bottom panels) Average transfer efficiency for the non-adiabatic transition events over time.

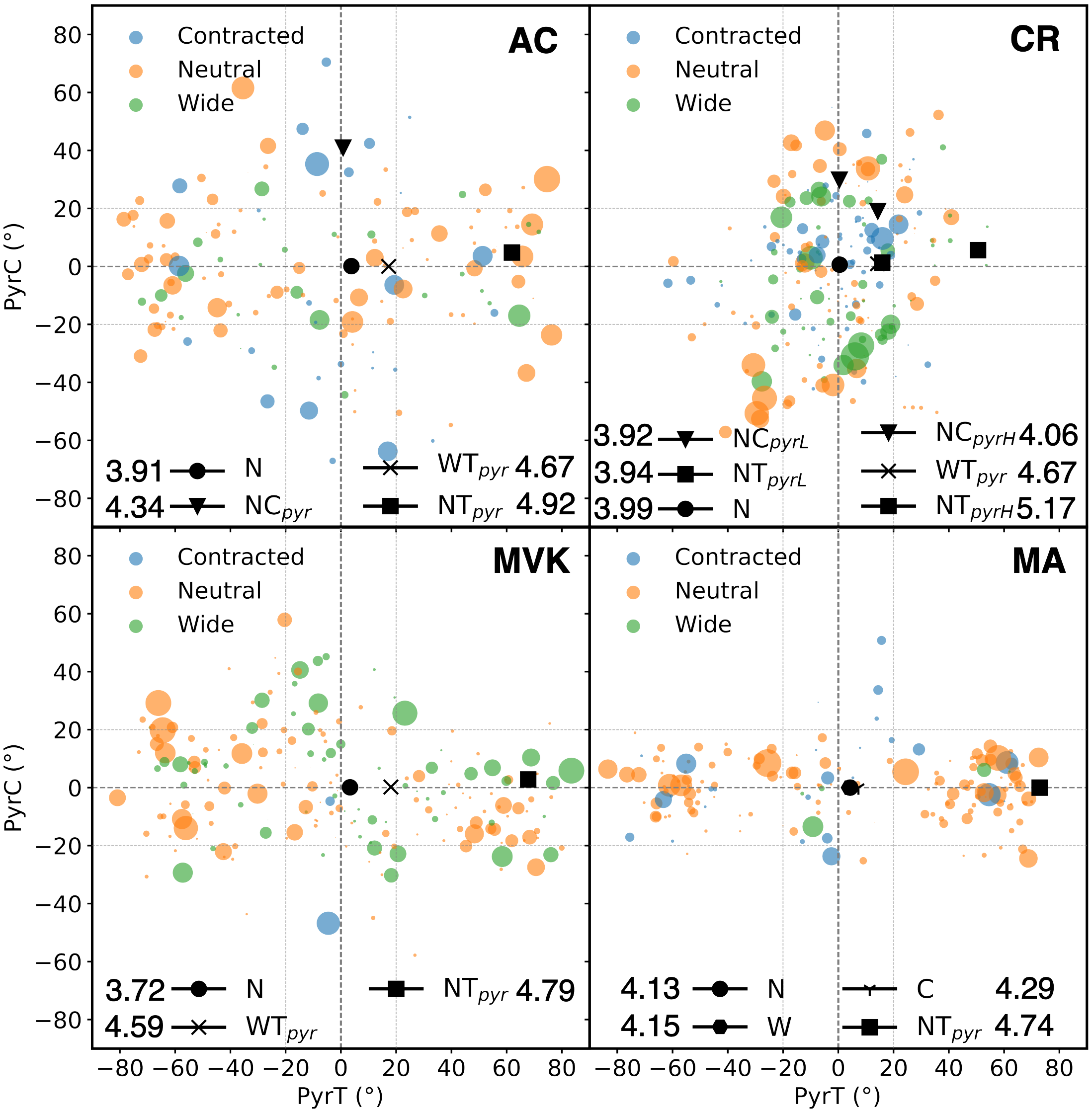

Figure S24: (PyrT, PyrC) distribution for early S_1/S_0 transition events (≤120 fs). The area of each circle represents the absolute population transfer scaled by a factor of two, with respect to the Figure 6, and classified according to their central angle (coloring). Identified S_1/S_0-MECIs are indicated by black markers and their energies relative to S_0-min are given in eV. Vertical and horizontal dashed lines serve as guides for the eye.

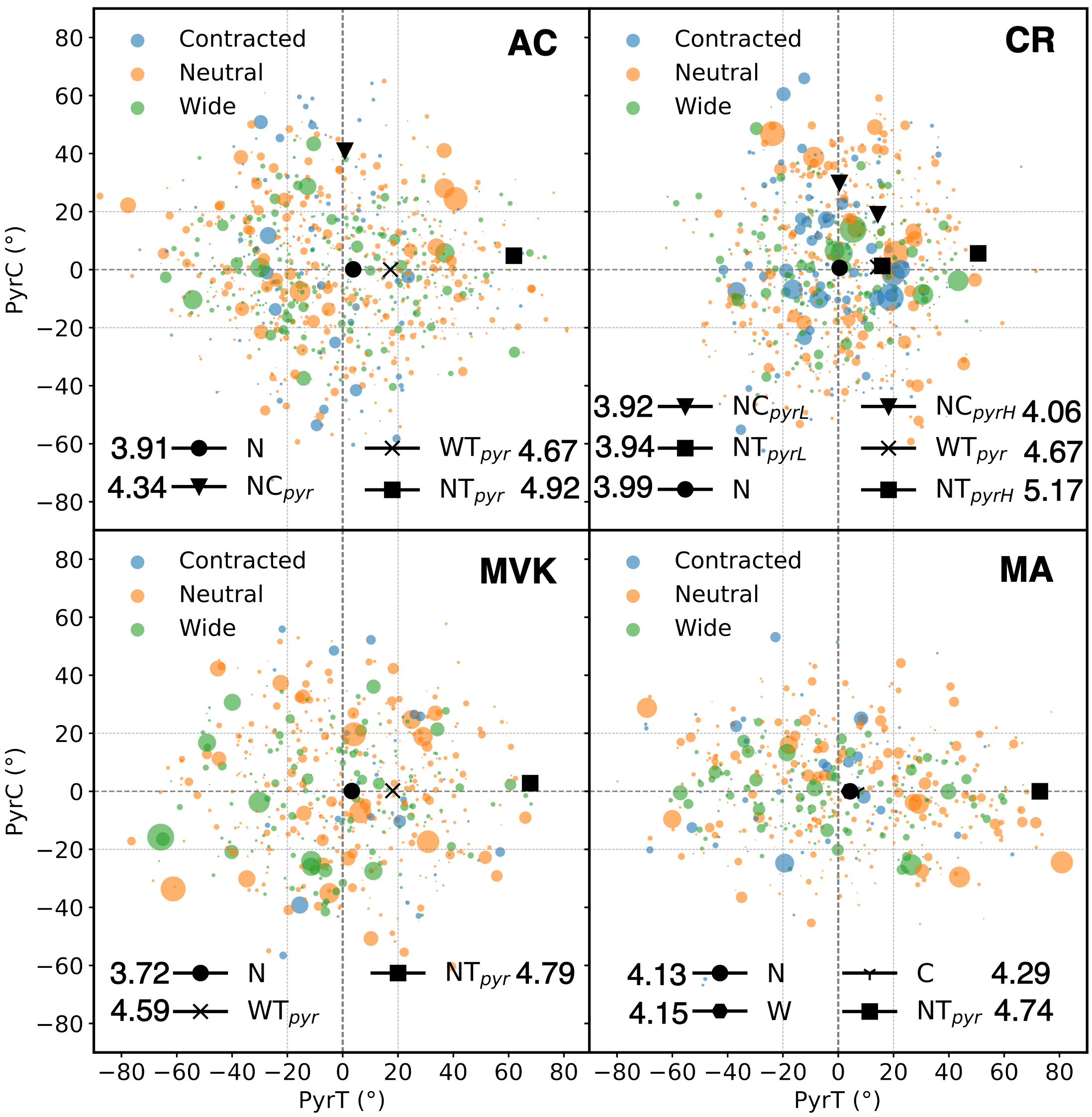

Figure S25: (PyrT, PyrC) distribution for late S_1/S_0 transition events (>120 fs). The area of each circle represents the absolute population transfer scaled by a factor of two with respect to Figure 6, and classified according to their central angle (coloring). Identified S_1/S_0-MECIs are indicated by black markers and their energies relative to S_0-min are provided in eV. Vertical and horizontal dashed lines serve as guides for the eye.

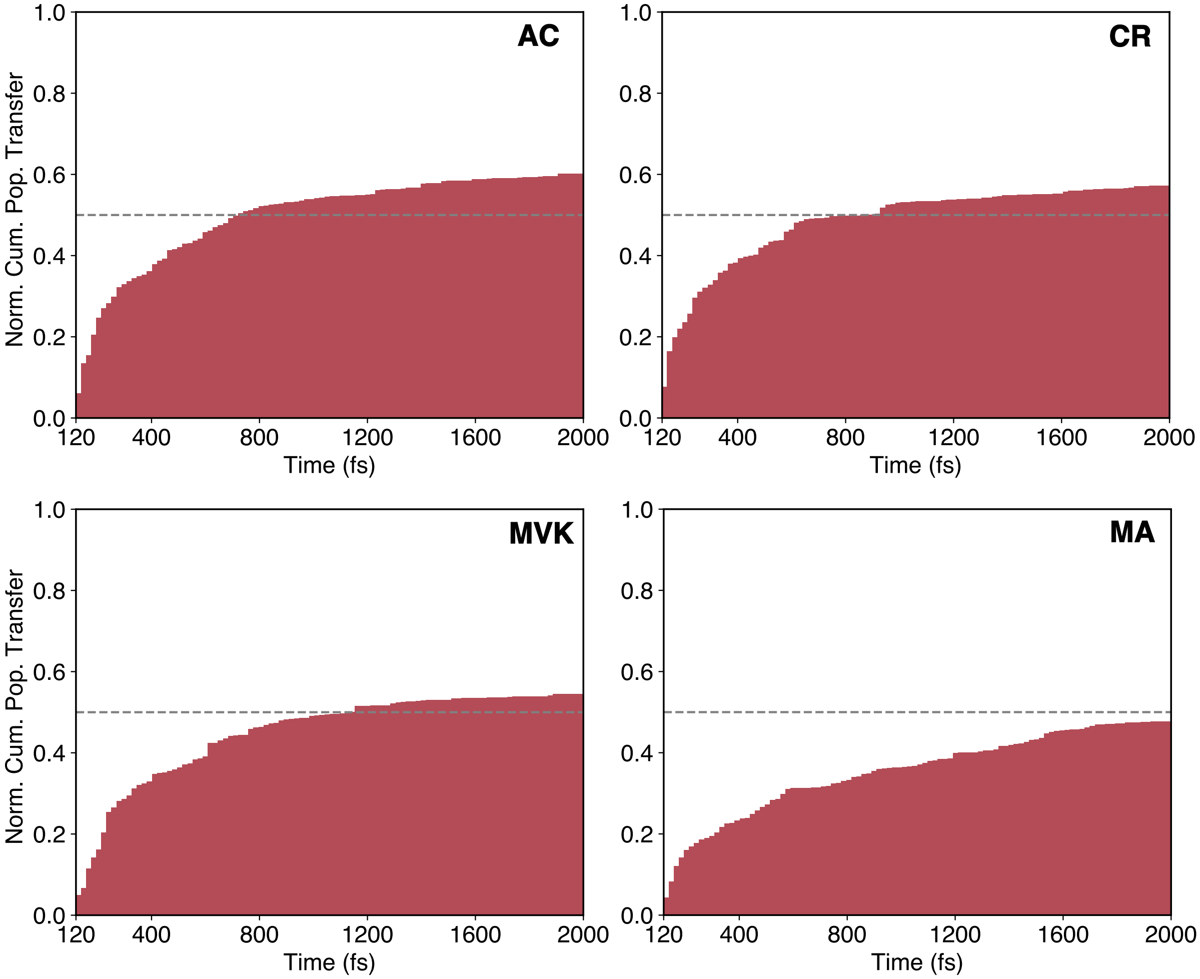

Figure S26: Normalized cumulative population transfer of S_1/S_0 non-adiabatic events for (a) AC, (b) CR, (c) MVK, and (d) MA, for late delay (between 120–2000 fs). The gray-dashed line is guide for the eye. For MA, significantly less population transfer occurs with respect to time, compared to other molecules.

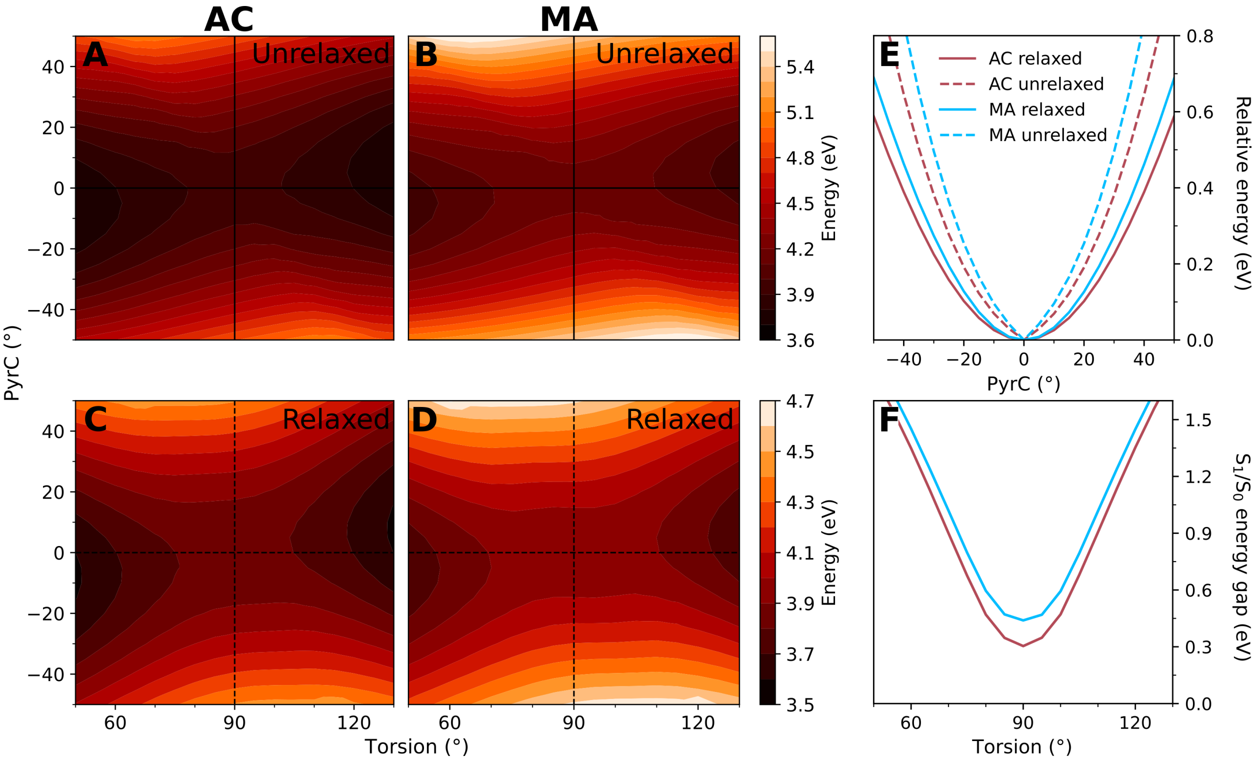

Figure S27: S_1 PES cuts along the PyrC and torsional modes for AC and MA as obtained from (A,B) an unrelaxed scan starting from S_1/S_0-MECI-N, and (C,D) a relaxed scan, relaxing all but the PyrC and torsional coordinates. Energies are given relative to their respective ground-state energy at the S_0-min. (E) One-dimensional cuts along PyrC (90° torsion) for both the relaxed and unrelaxed scans. (F) S_1/S_0 energy gaps along the torsional coordinate (no pyramidalization). Relaxation in MA brings the system further away from the intersection seam relative to AC.

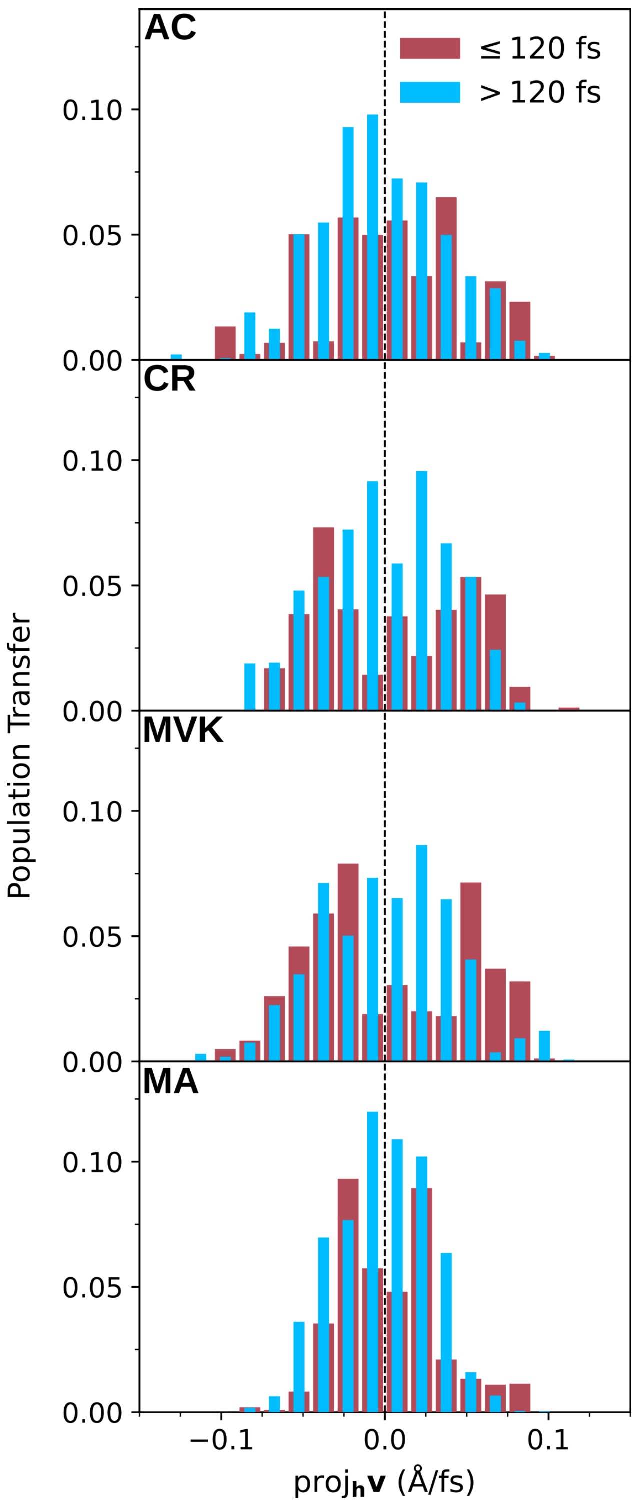

Figure S28: Distributions of the velocity components for the parent TBF along the h-direction (dominated by PyrC, Figure S29) at the S_1/S_0 non-adiabatic transition events divided into early (≤120 fs) and late (>120 fs) time bins.

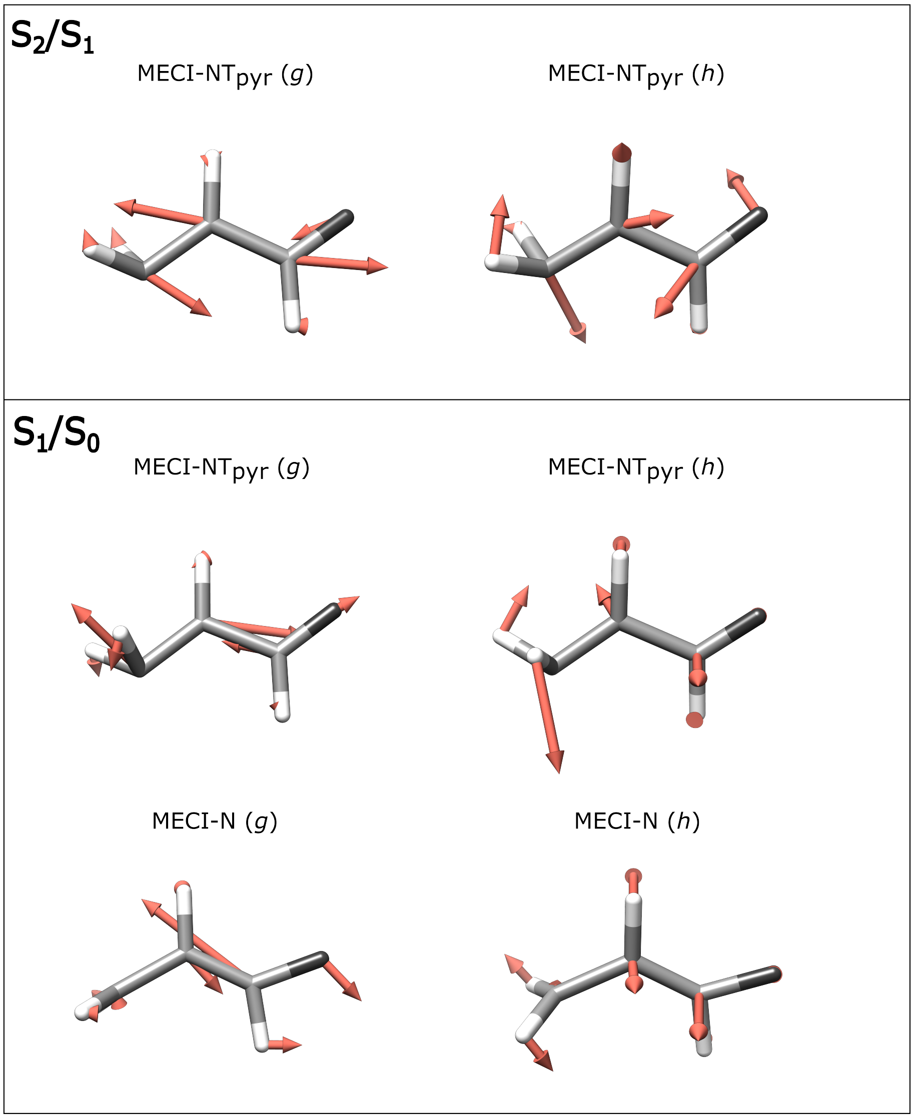

Figure S29: The gradient-difference (g) and non-adiabatic coupling (h) vectors of S_2/S_1-MECI-NT$_{pyr}$, S_1/S_0-MECI-NT$_{pyr}$ and S_1/S_0-MECI-N for AC. In all cases, the g-vector is dominated by C_1-C_2 stretching. The non-adiabatic coupling is dominated by pyramidalization with a small degree of torsion for S_1/S_0-MECI-N.

References

(1) Finley, J.; Malmqvist, P.-Å.; Roos, B. O.; Serrano-Andrés, L. The multi-state CASPT2 method. *Chem. Phys. Lett.* **1998**, *288*, 299–306.

(2) Granovsky, A. A. Extended multi-configuration quasi-degenerate perturbation theory: The new approach to multi-state multi-reference perturbation theory. *J. Chem. Phys.* **2011**, *134*, 214113.

(3) Shiozaki, T.; Győrffy, W.; Celani, P.; Werner, H.-J. Communication: Extended multi-state complete active space second-order perturbation theory: Energy and nuclear gradients. *J. Chem. Phys.* **2011**, *135*, 081106.

(4) Aquilante, F.; Barone, V.; Roos, B. O. A theoretical investigation of valence and Rydberg electronic states of acrolein. *J. Chem. Phys.* **2003**, *119*, 12323–12334.

(5) Loos, P.-F.; Boggio-Pasqua, M.; Scemama, A.; Caffarel, M.; Jacquemin, D. Reference energies for double excitations. *J. Chem. Theory Comput.* **2019**, *15*, 1939–1956.

(6) Saha, B.; Ehara, M.; Nakatsuji, H. Singly and doubly excited states of butadiene, acrolein, and glyoxal: Geometries and electronic spectra. *J. Chem. Phys.* **2006**, *125*, 014316.

(7) Loos, P.-F.; Lipparini, F.; Boggio-Pasqua, M.; Scemama, A.; Jacquemin, D. A mountaineering strategy to excited states: Highly accurate energies and benchmarks for medium sized molecules. *J. Chem. Theory Comput.* **2020**, *16*, 1711–1741.

(8) Budzák, v.; Scalmani, G.; Jacquemin, D. Accurate Excited-State Geometries: A CASPT2 and Coupled-Cluster Reference Database for Small Molecules. *J. Chem. Theory Comput.* **2017**, *13*, 6237–6252.

(9) Brémond, E.; Savarese, M.; Adamo, C.; Jacquemin, D. Accuracy of TD-DFT Geometries: A Fresh Look. *J. Chem. Theory Comput.* **2018**, *14*, 3715–3727.

(10) Hollas, J. The electronic absorption spectrum of acrolein vapour. *Spectrochim. Acta* **1963**, *19*, 1425–1441.

(11) Bouabça, T.; Ben Amor, N.; Maynau, D.; Caffarel, M. A study of the fixed-node error in quantum Monte Carlo calculations of electronic transitions: The case of the singlet $n \rightarrow \pi^*$ (CO) transition of the acrolein. *J. Chem. Phys.* **2009**, *130*, 114107.

(12) Fang, W.-H. A CASSCF study on photodissociation of acrolein in the Gas Phase. *J. Am. Chem. Soc.* **1999**, *121*, 8376–8384.

(13) Bokareva, O. S.; Bataev, V. A.; Pupyshev, V. I.; Godunov, I. A. Structure and dynamics of acrolein in lowest excited 1,3(n,π^*) electronic states: The quantum-chemical study. *Int. J. Quantum Chem.* **2008**, *108*, 2719–2731.

(14) Dunning Jr, T. H. Gaussian basis sets for use in correlated molecular calculations. I. The atoms boron through neon and hydrogen. *J. Chem. Phys.* **1989**, *90*, 1007–1023.

(15) Park, J. W. Single-state single-reference and multistate multireference zeroth-order Hamiltonians in MS-CASPT2 and conical intersections. *J. Chem. Theory and Comput.* **2019**, *15*, 3960–3973.

(16) Shiozaki, T. BAGEL: Brilliantly advanced general electronic-structure library. *WIREs Comput. Mol. Sci.* **2018**, *8*, e1331.

(17) BAGEL, Brilliantly Advanced General Electronic-structure Library. under the GNU General Public License. `http://www.nubakery.org`.

(18) Fdez. Galván, I.; Vacher, M.; Alavi, A.; Angeli, C.; Aquilante, F.; Autschbach, J.; Bao, J. J.; Bokarev, S. I.; Bogdanov, N. A.; Carlson, R. K.; Chibotaru, L. F.; Creutzberg, J.; Dattani, N.; Delcey, M. G.; Dong, S. S.; Dreuw, A.; Freitag, L.; Frutos, L. M.; Gagliardi, L.; Gendron, F.; Giussani, A.; González, L.; Grell, G.; Guo, M.; Hoyer, C. E.; Johansson, M.; Keller, S.; Knecht, S.; Kovačević, G.; Källman, E.;

Li Manni, G.; Lundberg, M.; Ma, Y.; Mai, S.; Malhado, J. a. P.; Malmqvist, P. Å.; Marquetand, P.; Mewes, S. A.; Norell, J.; Olivucci, M.; Oppel, M.; Phung, Q. M.; Pierloot, K.; Plasser, F.; Reiher, M.; Sand, A. M.; Schapiro, I.; Sharma, P.; Stein, C. J.; Sørensen, L. K.; Truhlar, D. G.; Ugandi, M.; Ungur, L.; Valentini, A.; Vancoillie, S.; Veryazov, V.; Weser, O.; Wesołowski, T. A.; Widmark, P.-O.; Wouters, S.; Zech, A.; Zobel, J. P.; Lindh, R. OpenMolcas: From Source Code to Insight. *J. Chem. Theory Comput.* **2019**, *15*, 5925–5964.

(19) Aquilante, F.; Autschbach, J.; Baiardi, A.; Battaglia, S.; Borin, V. A.; Chibotaru, L. F.; Conti, I.; De Vico, L.; Delcey, M.; Fdez. Galván, I.; Ferré, N.; Freitag, L.; Garavelli, M.; Gong, X.; Knecht, S.; Larsson, E. D.; Lindh, R.; Lundberg, M.; Malmqvist, P. Å.; Nenov, A.; Norell, J.; Odelius, M.; Olivucci, M.; Pedersen, T. B.; Pedraza-González, L.; Phung, Q. M.; Pierloot, K.; Reiher, M.; Schapiro, I.; Segarra-Martí, J.; Segatta, F.; Seijo, L.; Sen, S.; Sergentu, D.-C.; Stein, C. J.; Ungur, L.; Vacher, M.; Valentini, A.; Veryazov, V. Modern quantum chemistry with [Open]Molcas. *J. Chem. Phys.* **2020**, *152*, 214117.

(20) Reguero, M.; Olivucci, M.; Bernardi, F.; Robb, M. A. Excited-state potential surface crossings in acrolein: A model for understanding the photochemistry and photophysics of. alpha.,. beta.-enones. *J. Am. Chem. Soc.* **1994**, *116*, 2103–2114.

(21) Lee, A.; Coe, J.; Ullrich, S.; Ho, M.-L.; Lee, S.-J.; Cheng, B.-M.; Zgierski, M.; Chen, I.; Martinez, T.; Stolow, A. Substituent effects on dynamics at conical intersections: α, β-enones. *J. Phys. Chem. A* **2007**, *111*, 11948–11960.

(22) Hohenstein, E. G.; Yu, J. K.; Bannwarth, C.; List, N. H.; Paul, A. C.; Folkestad, S. D.; Koch, H.; Martínez, T. J. Predictions of pre-edge features in time-resolved near-edge X-ray absorption fine structure spectroscopy from hole–hole Tamm–Dancoff-Approximated density functional theory. *J. Chem. Theory Comput.* **2021**, *17*, 7120–7133.

(23) Fahr, A.; Braun, W.; Laufer, A. H. Photolysis of methyl vinyl ketone at 193.3 nm: quantum yield determinations of methyl and vinyl radicals. *J. Phys. Chem.* **1993**, *97*, 1502–1506.

(24) Birge, R. R.; Pringle, W. C.; Leermakers, P. A. Excited-state geometries of the singly substituted methylpropenals. I. Vibrational-electronic analysis of S1 (n,. pi.*). *J. Am. Chem. Soc.* **1971**, *93*, 6715–6726.

(25) Brooks, B. R.; Schaefer III, H. F. Sudden polarization: pyramidalization of twisted ethylene. *J. Am. Chem. Soc.* **1979**, *101*, 307–311.

(26) Barbatti, M.; Paier, J.; Lischka, H. Photochemistry of ethylene: A multireference configuration interaction investigation of the excited-state energy surfaces. *J. Chem. Phys.* **2004**, *121*, 11614–11624.

(27) Barbatti, M.; Ruckenbauer, M.; Lischka, H. The photodynamics of ethylene: A surface-hopping study on structural aspects. *J. Chem. Phys.* **2005**, *122*, 174307.

(28) Tao, H.; Levine, B. G.; Martínez, T. J. Ab initio multiple spawning dynamics using multi-state second-order perturbation theory. *J. Phys. Chem. A* **2009**, *113*, 13656–13662.

(29) Glover, W. J.; Mori, T.; Schuurman, M. S.; Boguslavskiy, A. E.; Schalk, O.; Stolow, A.; Martínez, T. J. Excited state non-adiabatic dynamics of the smallest polyene, trans 1, 3-butadiene. II. Ab initio multiple spawning simulations. *J. Chem. Phys.* **2018**, *148*, 164303.

(30) MacDonell, R. J.; Corrales, M. E.; Boguslavskiy, A. E.; Bañares, L.; Stolow, A.; Schuurman, M. S. Substituent effects on nonadiabatic excited state dynamics: Inertial, steric, and electronic effects in methylated butadienes. *J. Chem. Phys.* **2020**, *152*, 084308.

(31) Michl, J.; Bonacic-Koutecky, V. *Electronic aspects of organic photochemistry*; Wiley, 1990.

(32) Levine, B. G.; Martínez, T. J. Ab initio multiple spawning dynamics of excited butadiene: Role of charge transfer. *J. Phys. Chem. A* **2009**, *113*, 12815–12824.

(33) Cui, G.; Thiel, W. Generalized trajectory surface-hopping method for internal conversion and intersystem crossing. *J. Chem. Phys.* **2014**, *141*, 124101.

(34) Avagliano, D.; Bonfanti, M.; Garavelli, M.; González, L. QM/MM nonadiabatic dynamics: the SHARC/COBRAMM approach. *J. Chem. Theory Comput.* **2021**, *17*, 4639–4647.

(35) Yarkony, D. R. On the adiabatic to diabatic states transformation near intersections of conical intersections. *J. Chem. Phys.* **2000**, *112*, 2111–2120.

(36) Zhu, X.; Thompson, K. C.; Martínez, T. J. Geodesic interpolation for reaction pathways. *J. Chem. Phys.* **2019**, *150*, 164103.
